# Supplementary material for: An artificial metalloenzyme biosensor can detect ethylene gas in fruits and Arabidopsis leaves
Source: Nat Commun. 2019 Dec 17;10:5746. doi: 10.1038/s41467-019-13758-2 (PMC6917813; doi:10.1038/s41467-019-13758-2)
Supplement: Supplementary file 1 — Supplementary Information [file 41467_2019_13758_MOESM1_ESM.pdf]

Supplementary Information

An artificial metalloenzyme biosensor can detect ethylene gas in fruits and Arabidopsis leaves

Kenward Vong,[1,7] Shohei Eda,[1,2,7] Yasuhiro Kadota,[3] Takanori Wakatake,[3] Igor Nasibullin,[1] Satoshi Yokoshima,[4] Ken Shirasu,[3] and Katsunori Tanaka*[1,2,5,6]

[1] Biofunctional Synthetic Chemistry Laboratory, RIKEN Cluster for Pioneering Research, 2-1 Hirosawa, Wako-shi, Saitama, 351-0198, Japan

[2] GlycoTargeting Research Laboratory, RIKEN Baton Zone Program, 2-1 Hirosawa, Wako-shi, Saitama, 351-0198, Japan

[3] Plant Immunity Research Group, RIKEN Center for Sustainable Resource Science, 1-7-22 Suehiro-cho, Tsurumi, Yokohama, Kanagawa, 230-0045, Japan

[4] Graduate School of Pharmaceutical Sciences, Nagoya University, Furo-cho, Chikusa-ku, Nagoya, 464-8601, Japan

[5] Biofunctional Chemistry Laboratory, A. Butlerov Institute of Chemistry, Kazan Federal University, 18 Kremlyovskaya street, Kazan, 420008, Russia

[6] Department of Chemical Science and Engineering, School of Materials and Chemical Technology, Tokyo Institute of Technology, 2-12-1 O-okayama, Meguro-ku, Tokyo, 152-8552, Japan

[7] These authors contributed equally: Kenward Vong, Shohei Eda.

Corresponding Author E-mail: kotzenori@riken.jp

Table of Contents

Supplementary Figures 2
Supplementary Methods 68
Supplementary References.. 69

Supplementary Figures

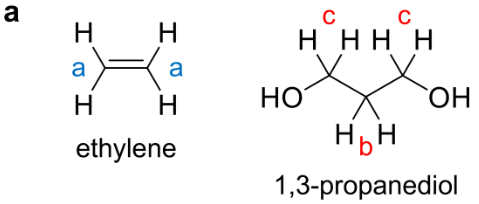

b

Solvent (% THF in H_2O)	Integral of b	Integral of a	Dissolved ethylene conc. (mM)
100%	2.00	0.4268	~1 mM
25%	2.00	-	undetectable
10%	2.00	-	undetectable

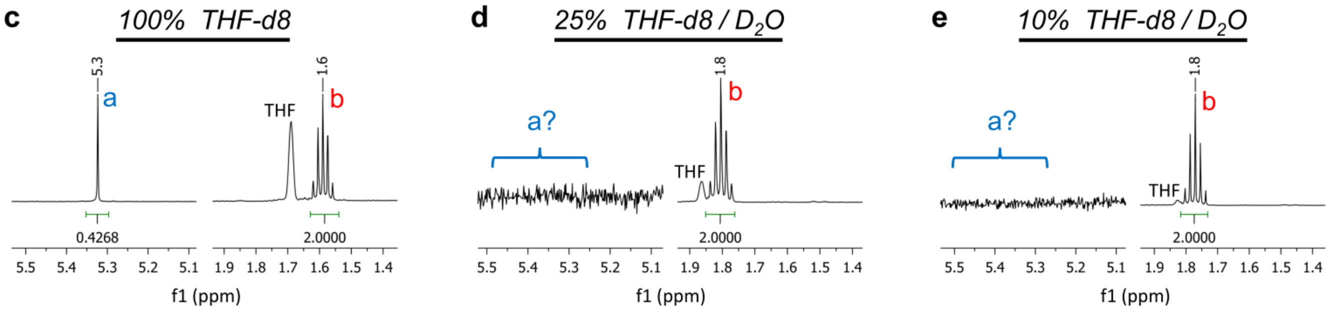

Supplementary Figure 1. NMR-based solubility study of ethylene gas. (**a**) Chemical structures of ethylene and 1,3-propanediol (internal NMR standard). (**b**) Chart comparing the integration ratios of peak a-to-b, thereby allowing the calculation of dissolved ethylene. NMR peaks are shown for the solvents of 100% THF (**c**), 25% THF in H_2O (**d**), and 10% THF in H_2O (**e**). To perform the experiment, 1 ml of ethylene gas was added to airtight sealed NMR tubes containing varying solvent concentrations. Peaks were integrated and then compared to peaks of the internal standard (10 mM of 1,3-propanediol).

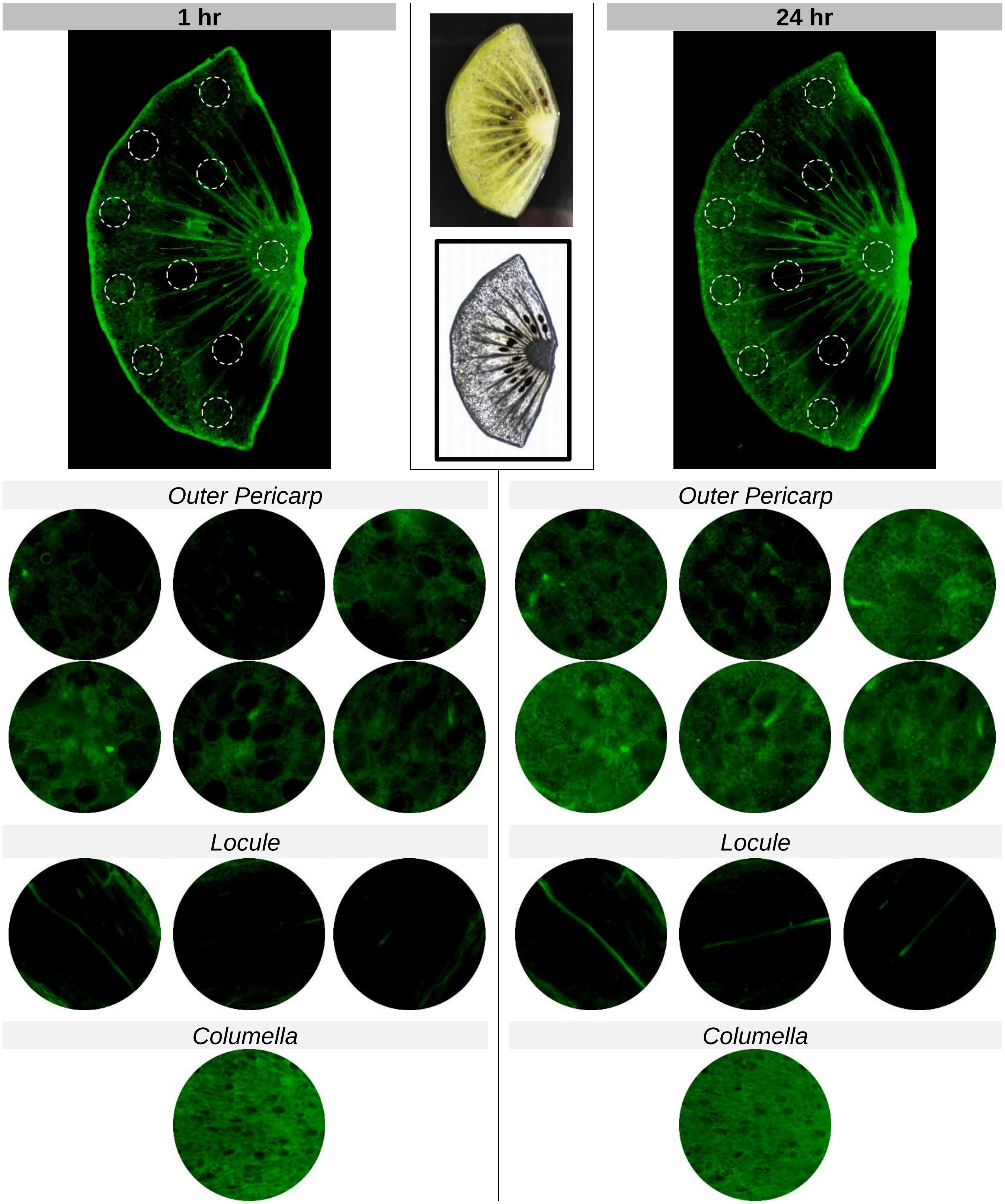

Supplementary Figure 2. Spatial imaging of ripening kiwifruit (sample#1), with emphasis on the outer pericarp, locules, and columella.

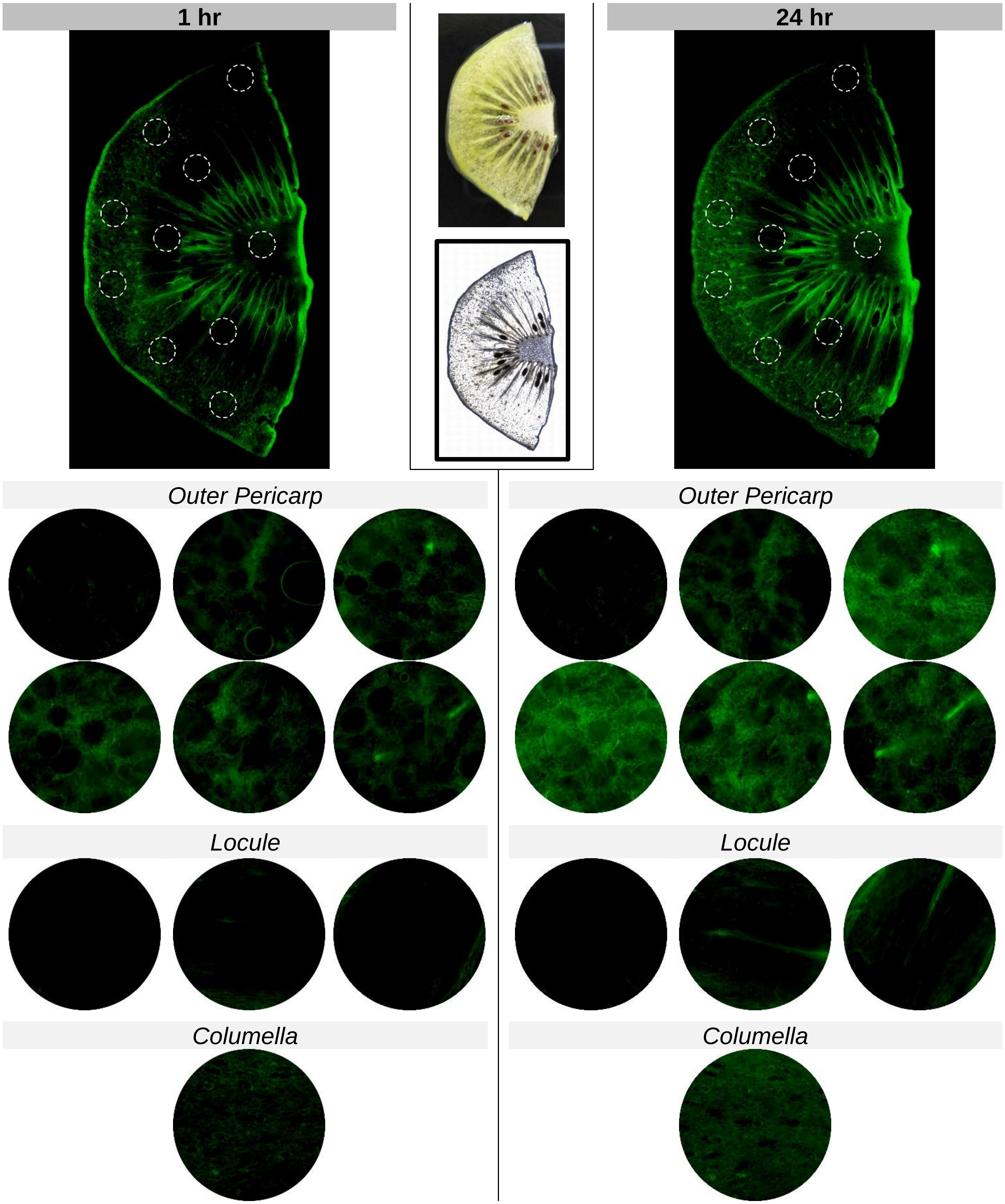

Supplementary Figure 3. Spatial imaging of ripening kiwifruit (sample#2), with emphasis on the outer pericarp, locules, and columella.

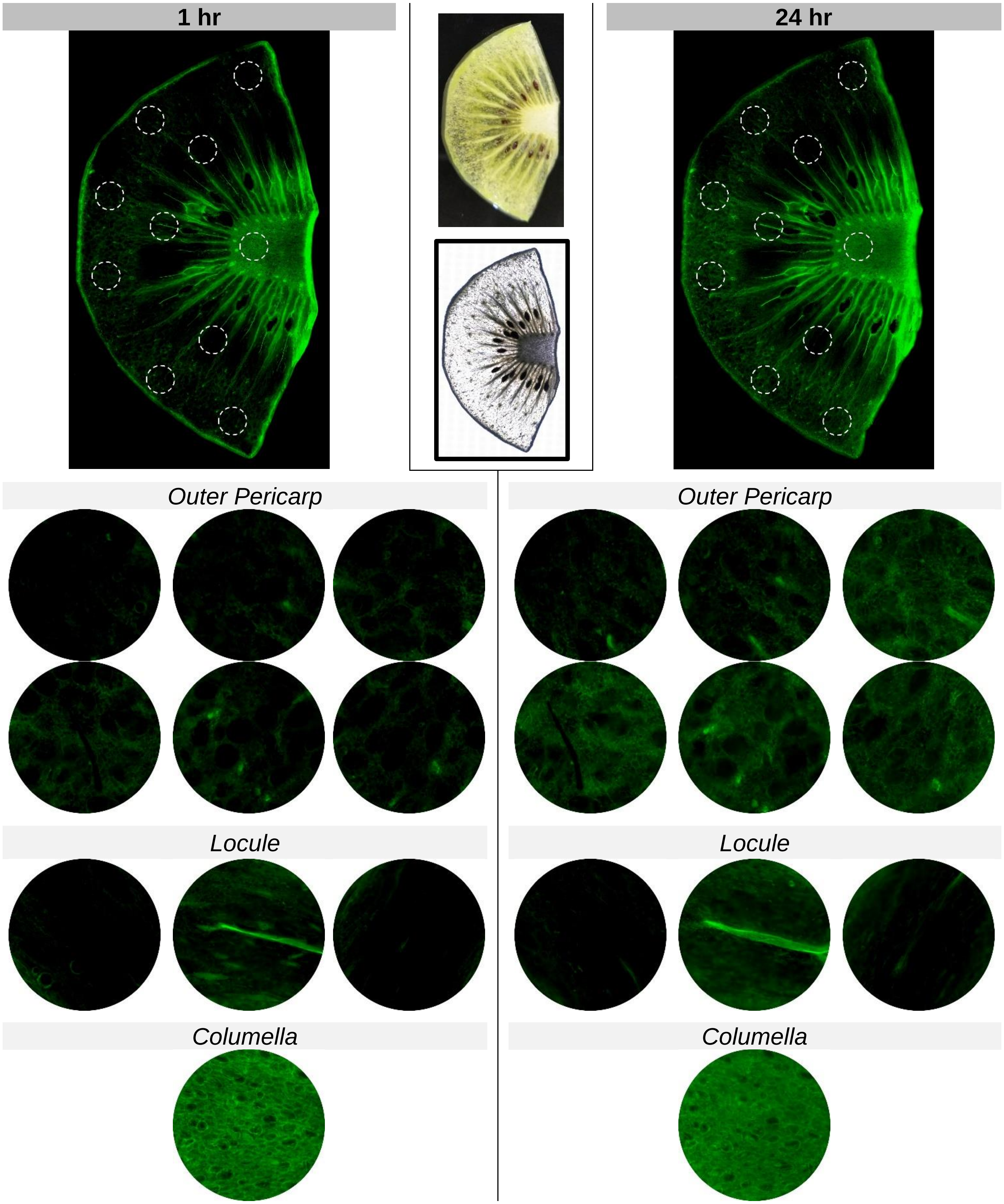

Supplementary Figure 4. Spatial imaging of ripening kiwifruit (sample#3), with emphasis on the outer pericarp, locules, and columella.

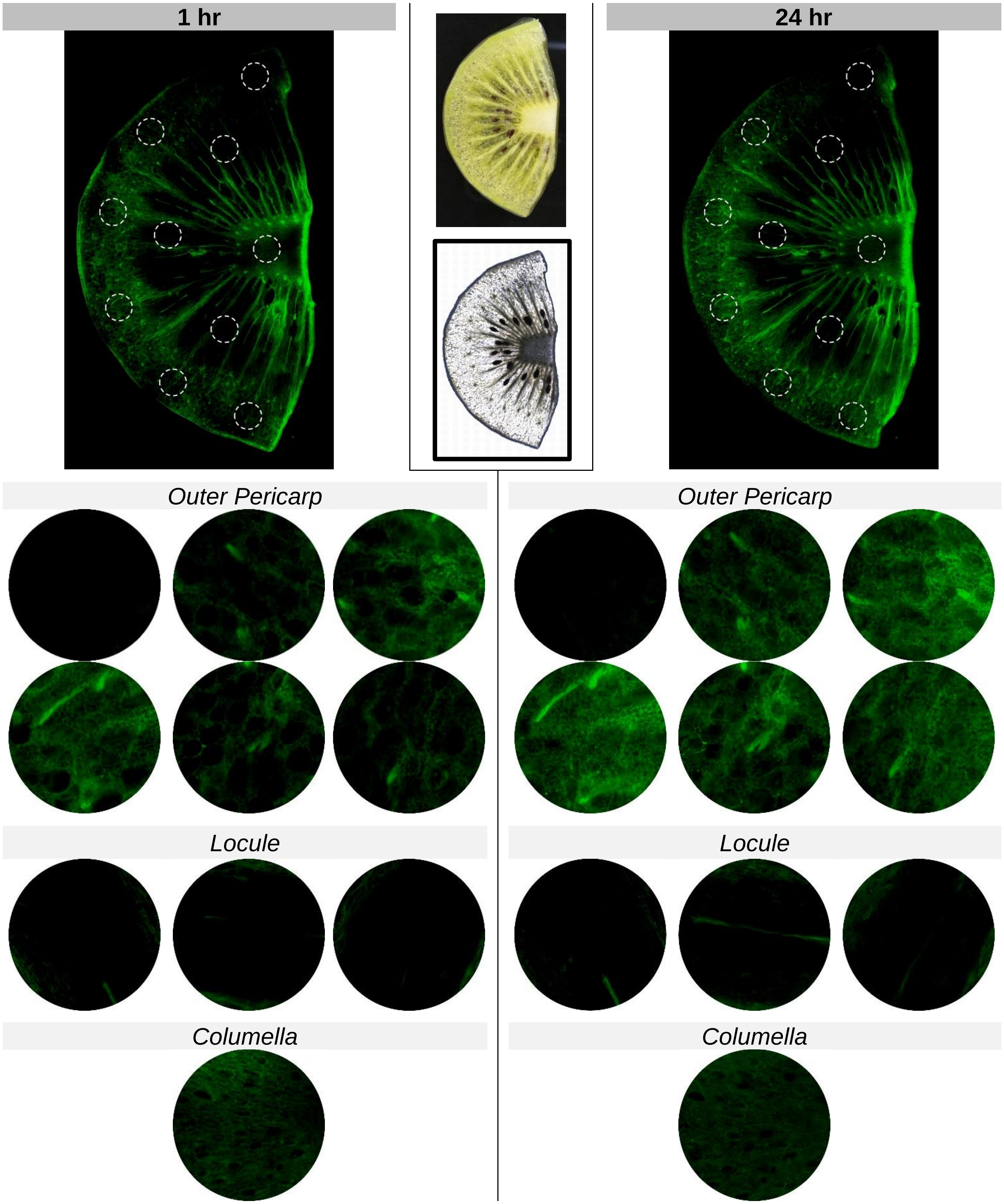

Supplementary Figure 5. Spatial imaging of ripening kiwifruit (sample#4), with emphasis on the outer pericarp, locules, and columella.

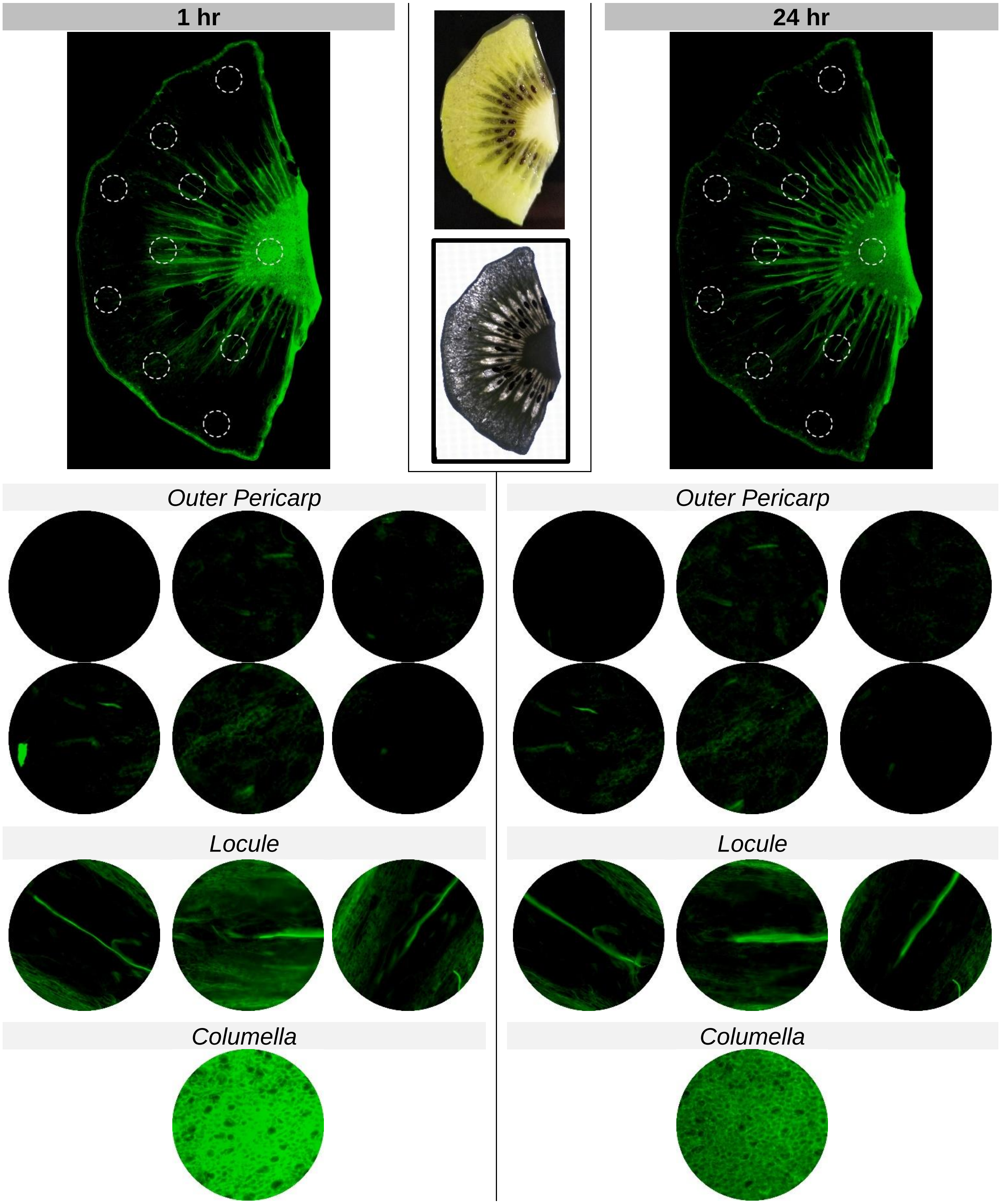

Supplementary Figure 6. Spatial imaging of unripe kiwifruit (sample#1), with emphasis on the outer pericarp, locules, and columella.

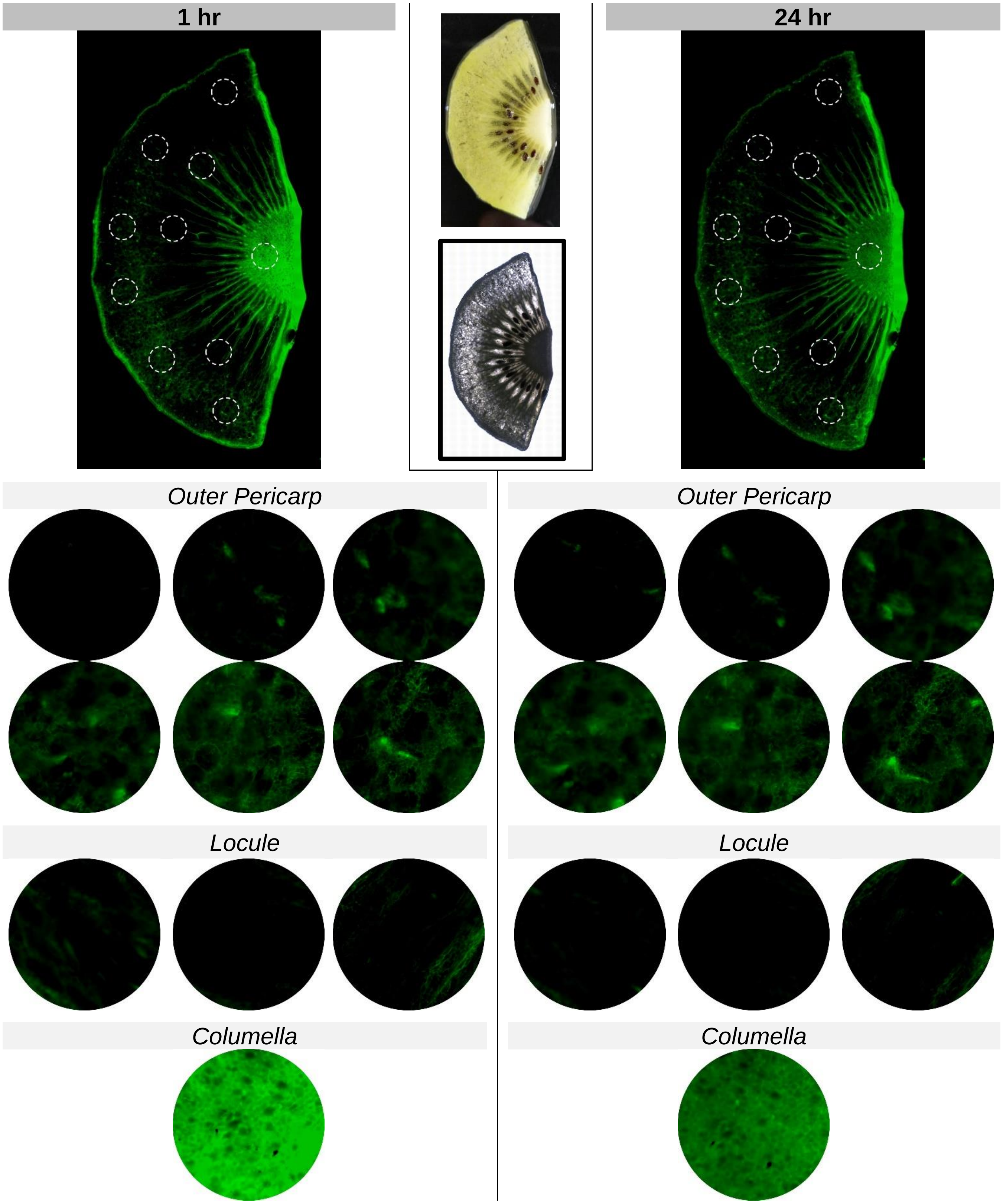

Supplementary Figure 7. Spatial imaging of unripe kiwifruit (sample#2), with emphasis on the outer pericarp, locules, and columella.

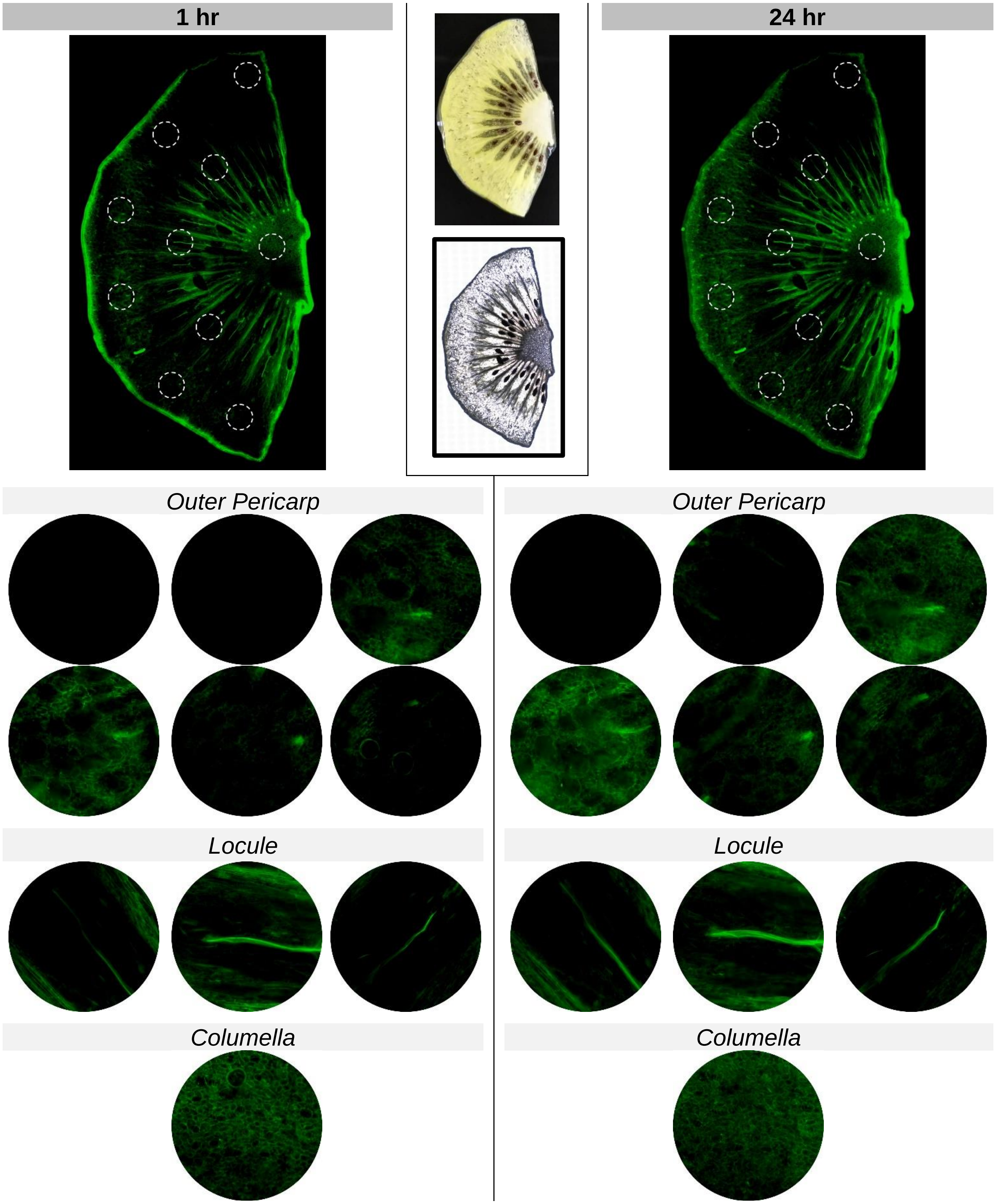

Supplementary Figure 8. Spatial imaging of unripe kiwifruit (sample#3), with emphasis on the outer pericarp, locules, and columella.

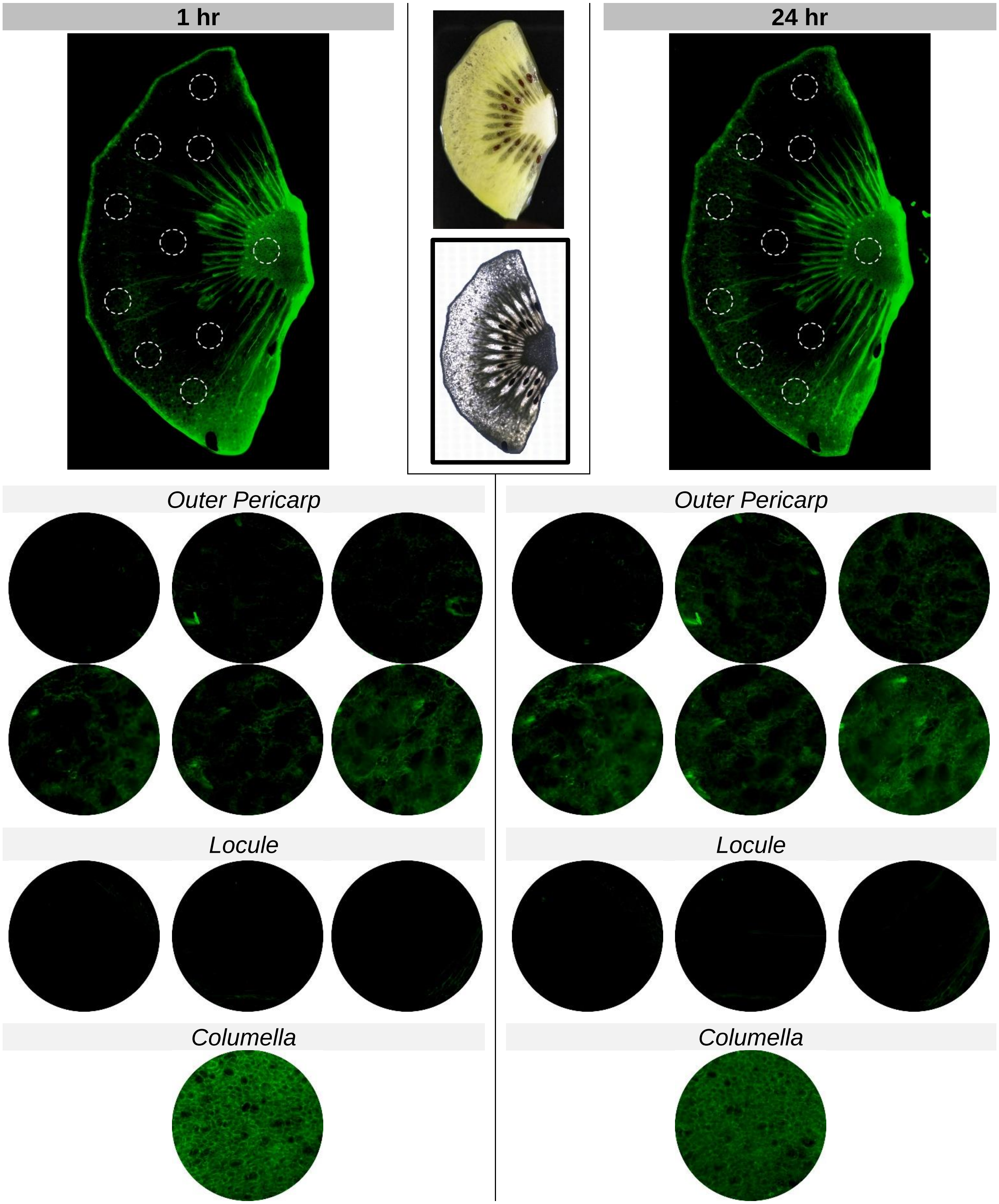

Supplementary Figure 9. Spatial imaging of unripe kiwifruit (sample#4), with emphasis on the outer pericarp, locules, and columella.

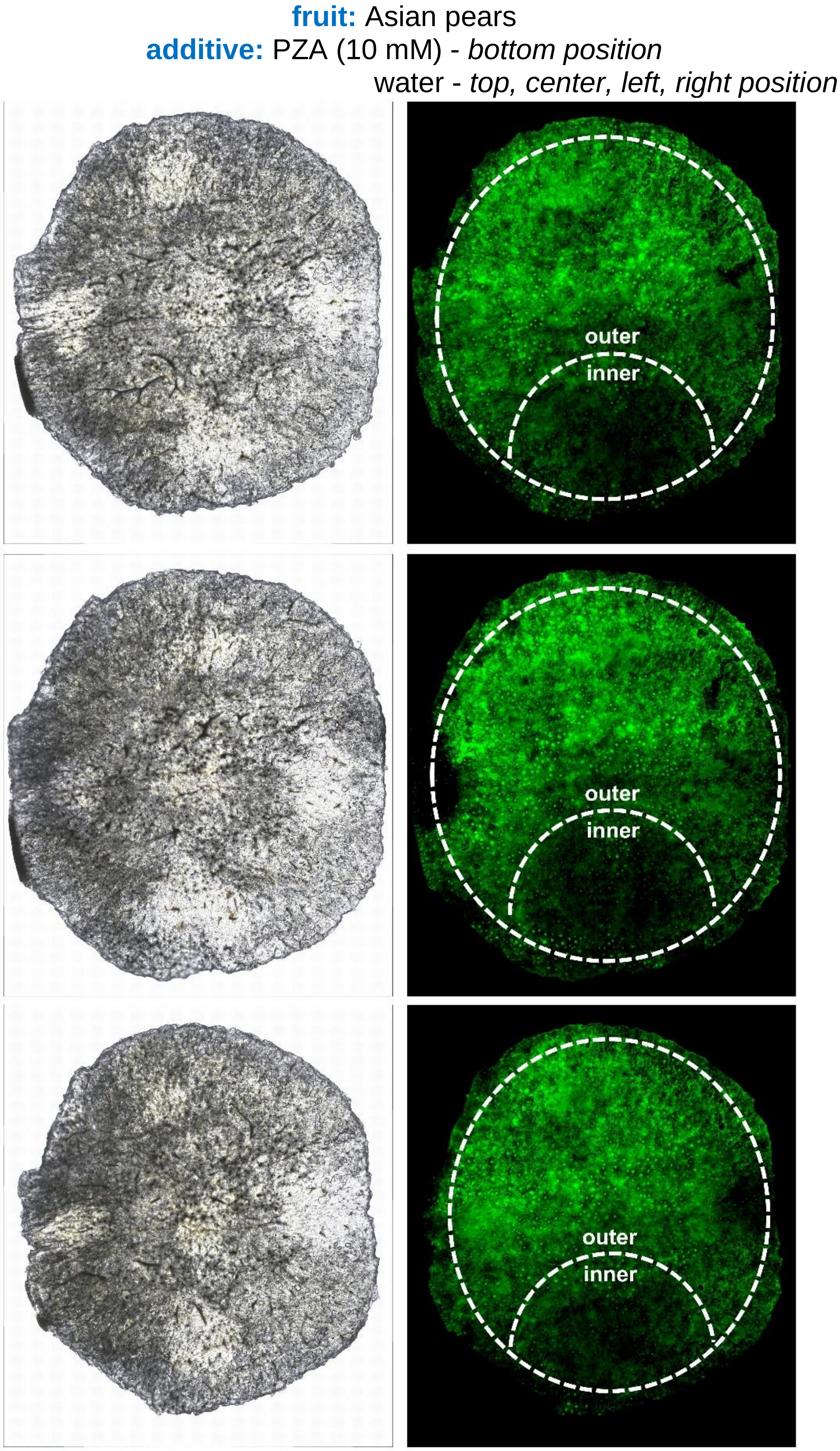

Supplementary Figure 10. Spatial imaging of Asian pear sections supplemented with 10 mM PZA (inner area) and water (outer area).

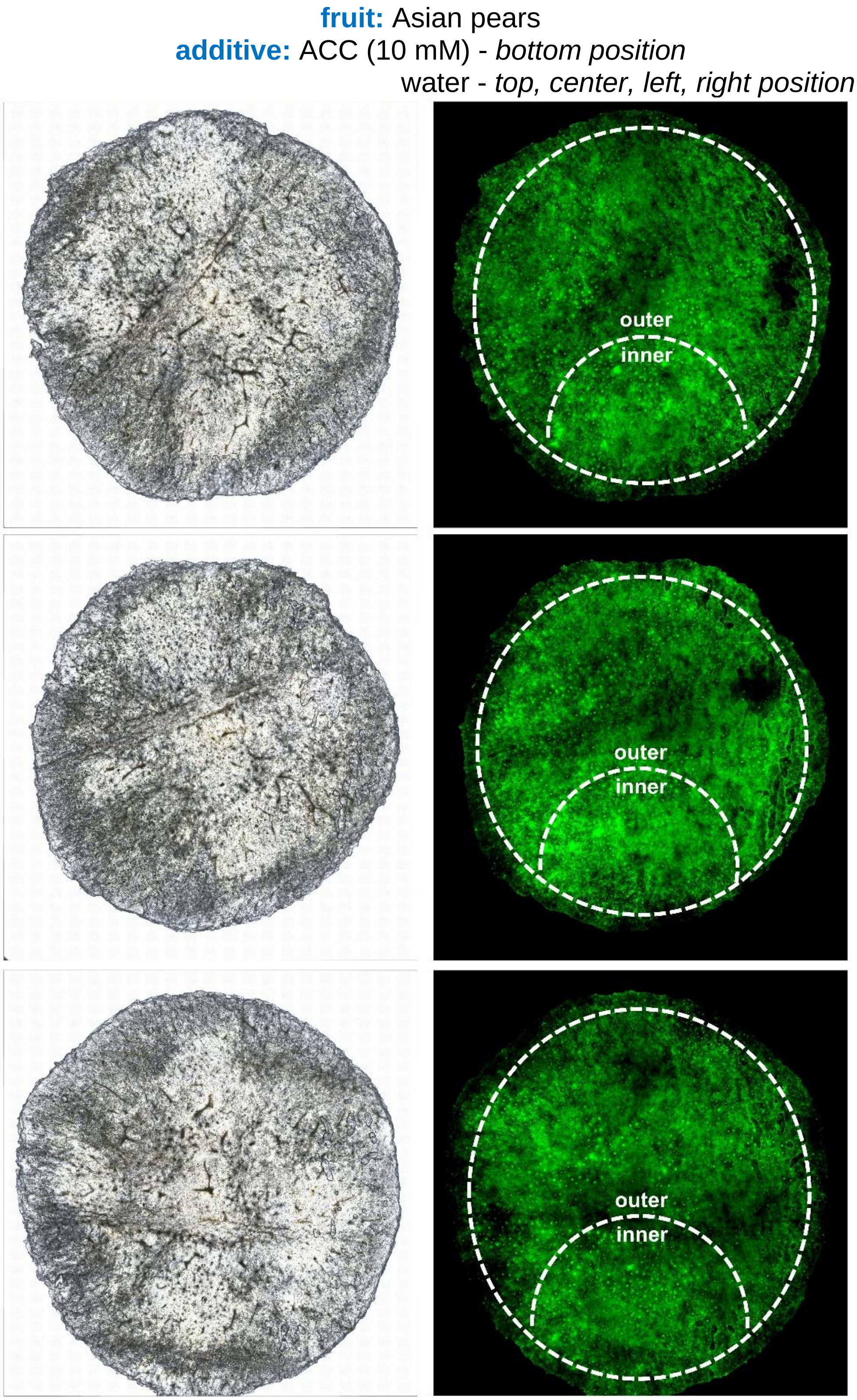

Supplementary Figure 11. Spatial imaging of Asian pear sections supplemented with 10 mM ACC (inner area) and water (outer area).

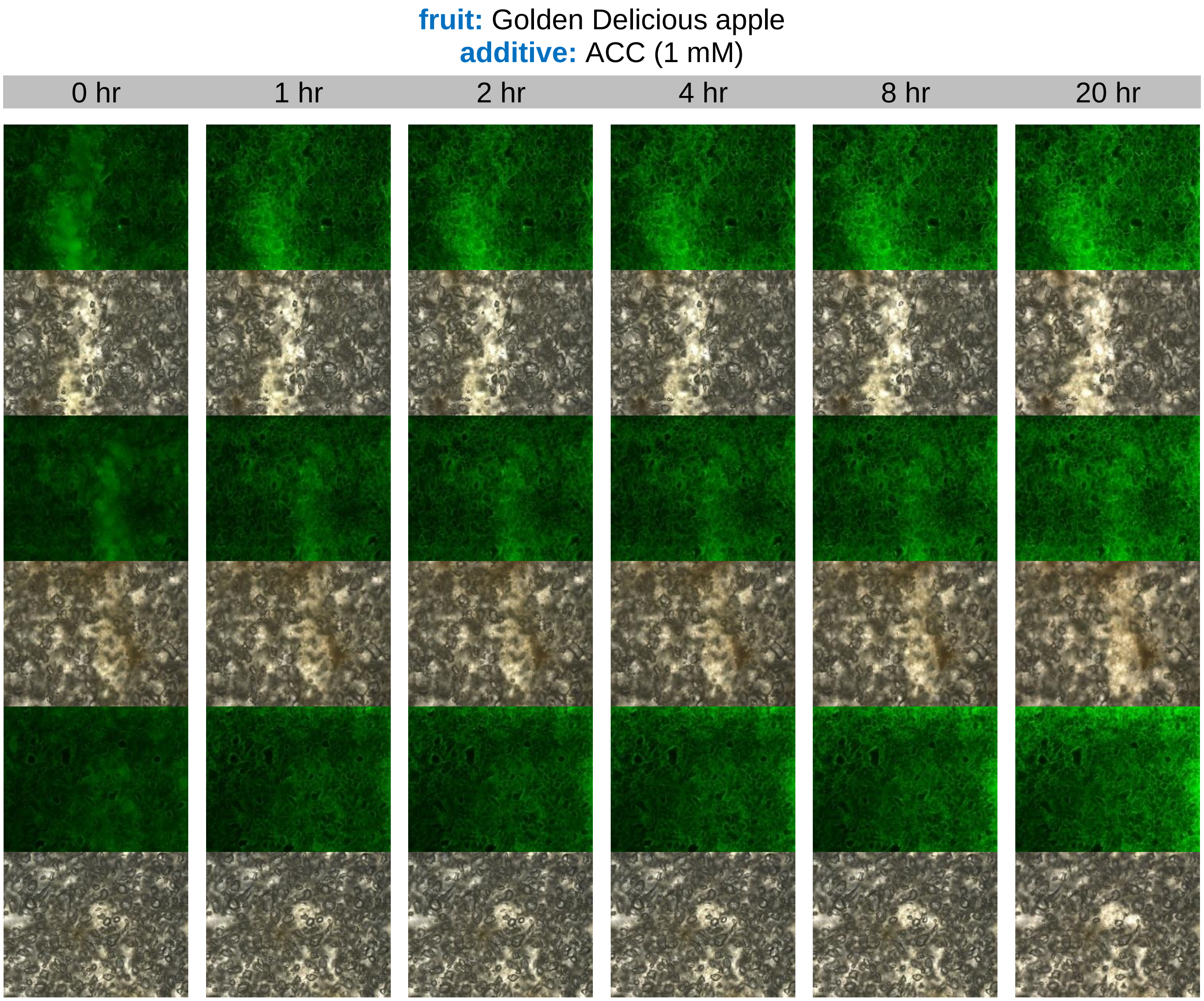

Supplementary Figure 12. Time-based imaging of Golden Delicious apples with 1 mM ACC supplementation.

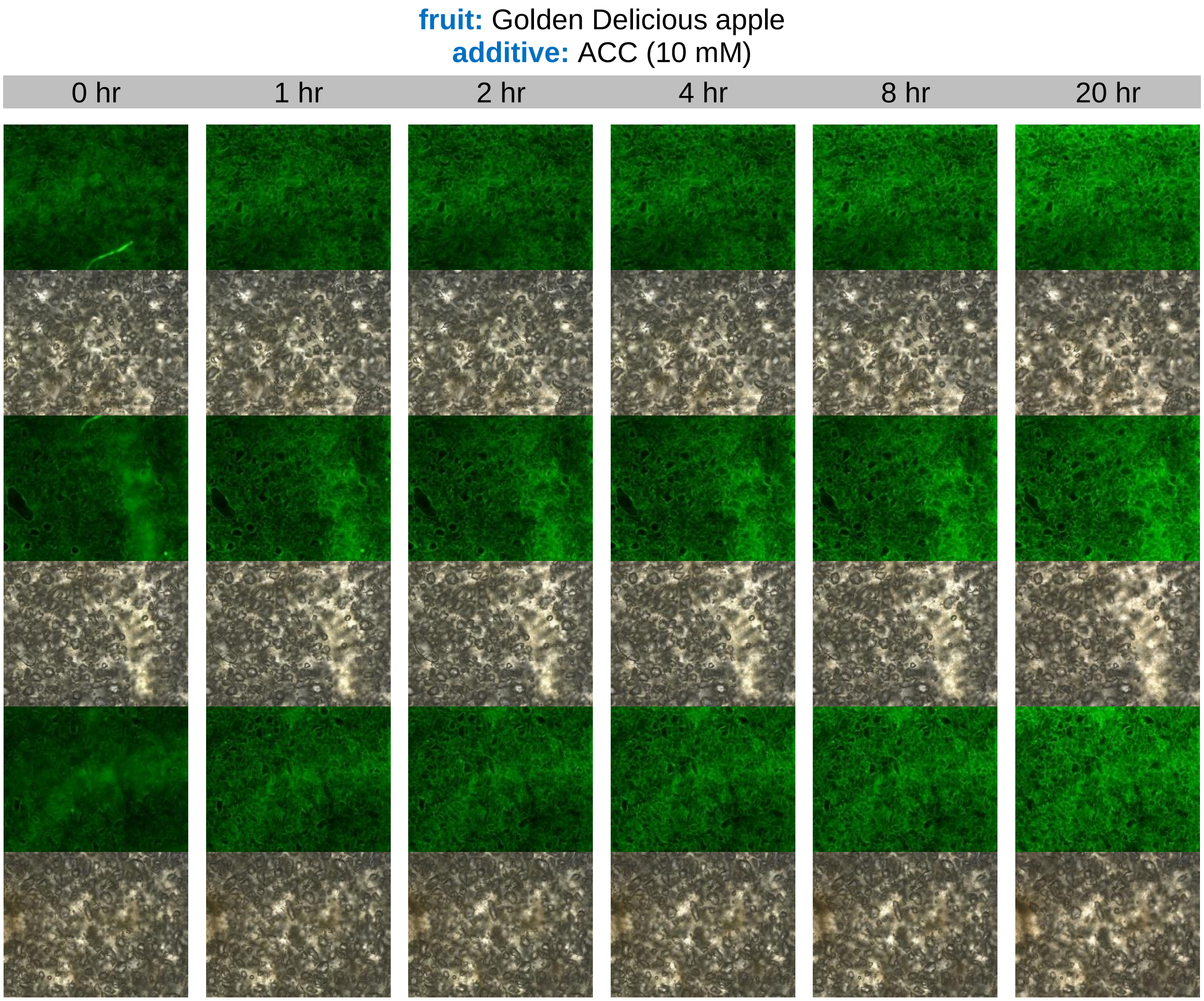

Supplementary Figure 13. Time-based imaging of Golden Delicious apples with 10 mM ACC supplementation.

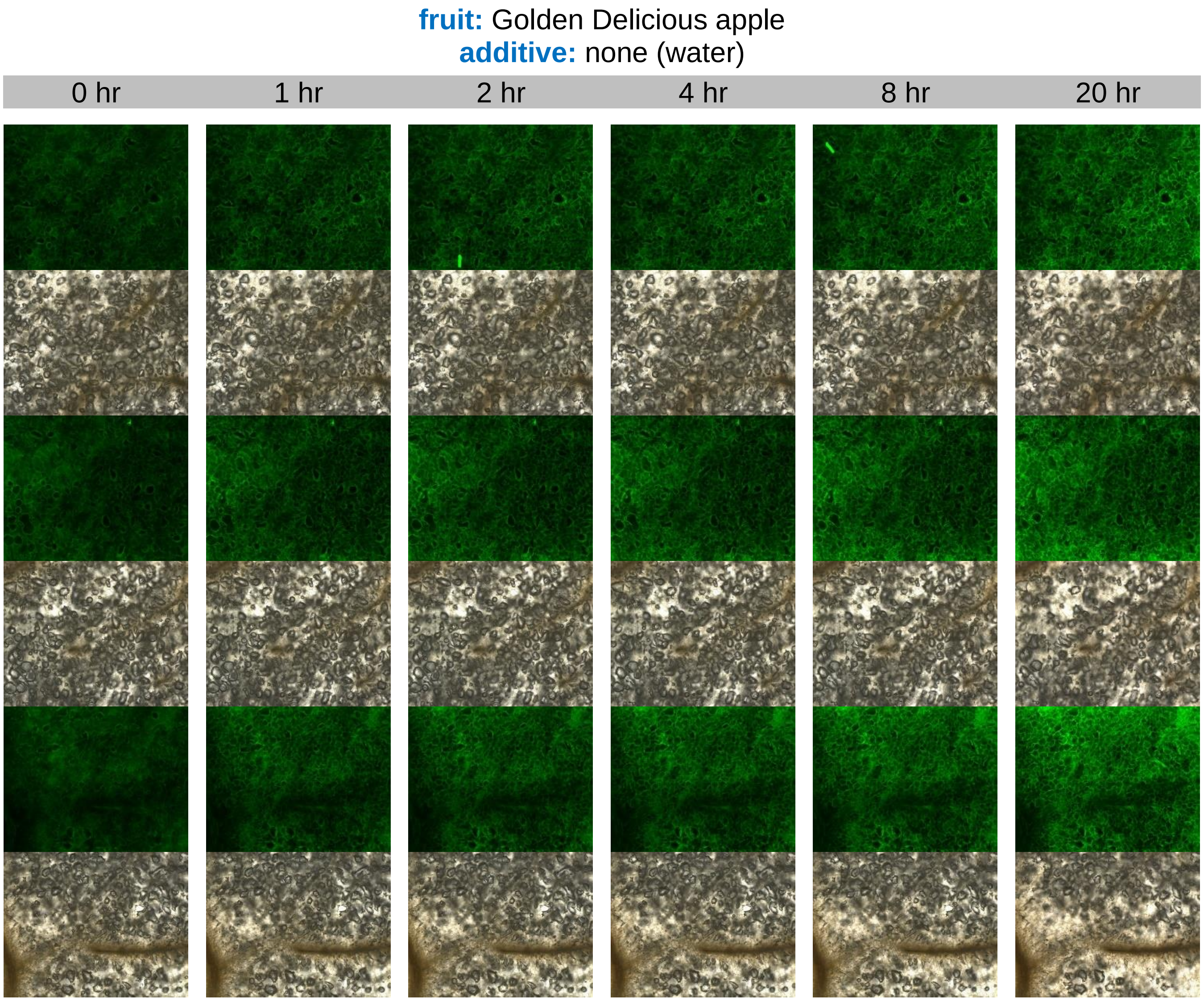

Supplementary Figure 14. Time-based imaging of Golden Delicious apples with no supplementation.

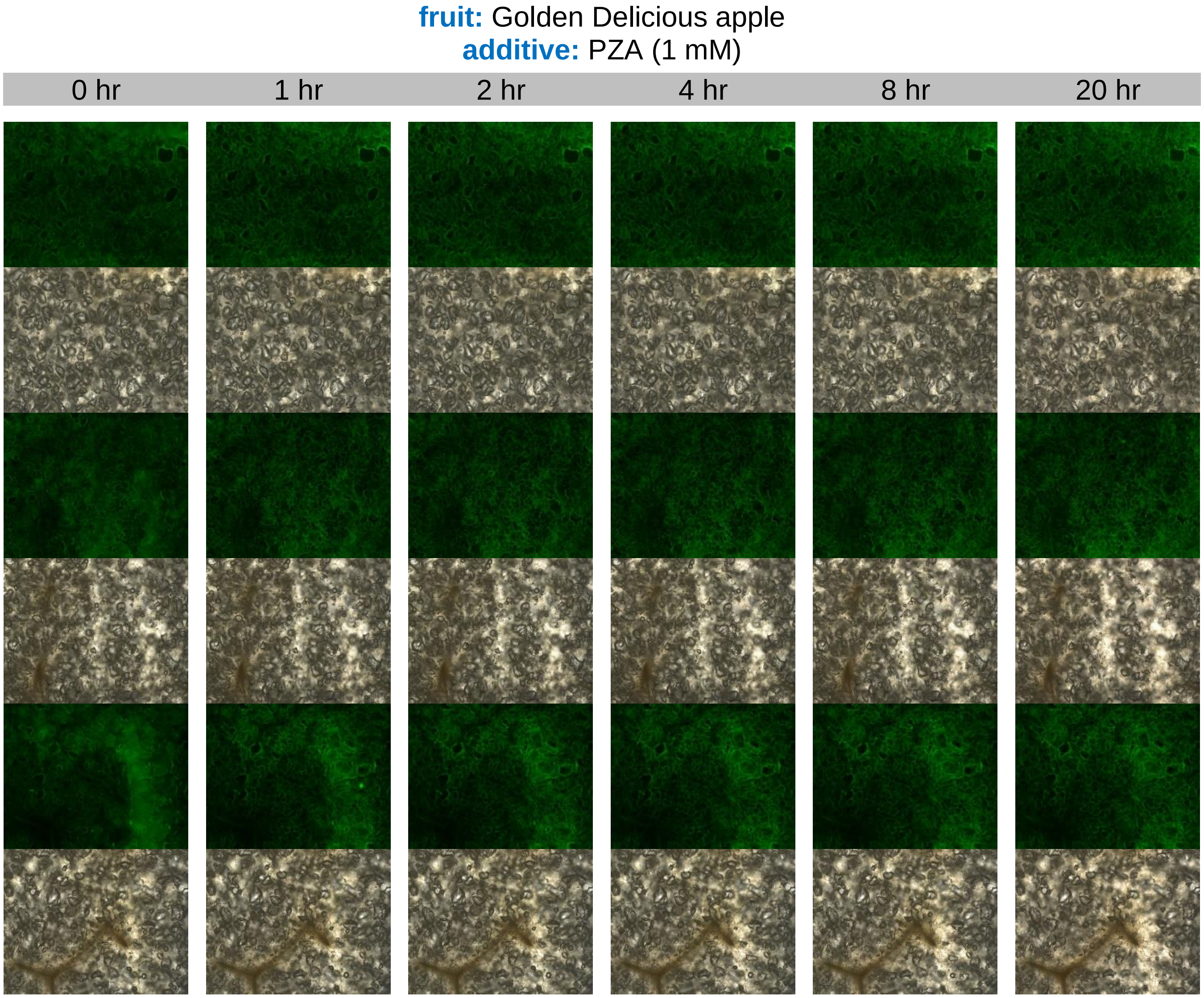

Supplementary Figure 15. Time-based imaging of Golden Delicious apples with 1 mM PZA supplementation.

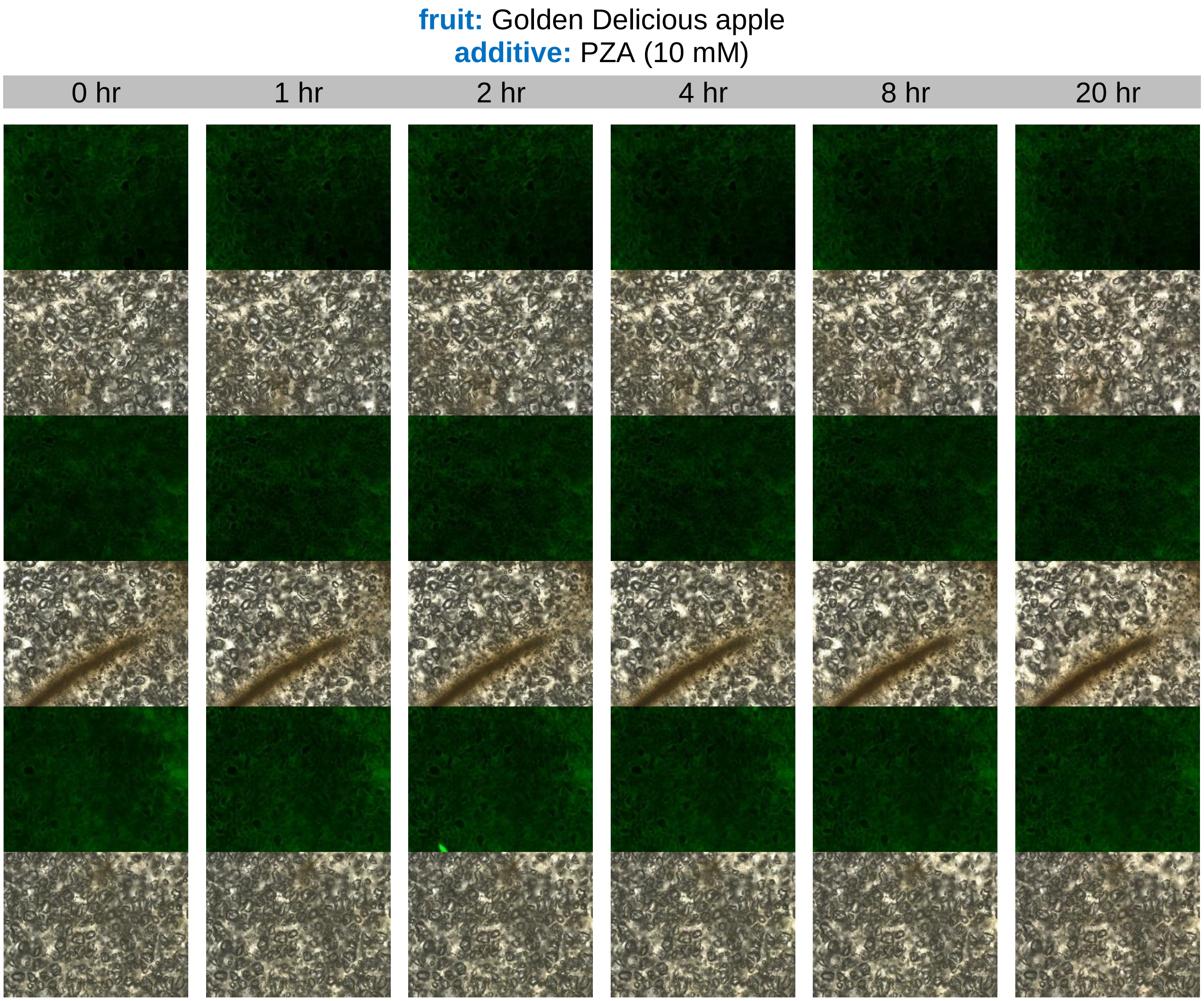

Supplementary Figure 16. Time-based imaging of Golden Delicious apples with 10 mM PZA supplementation.

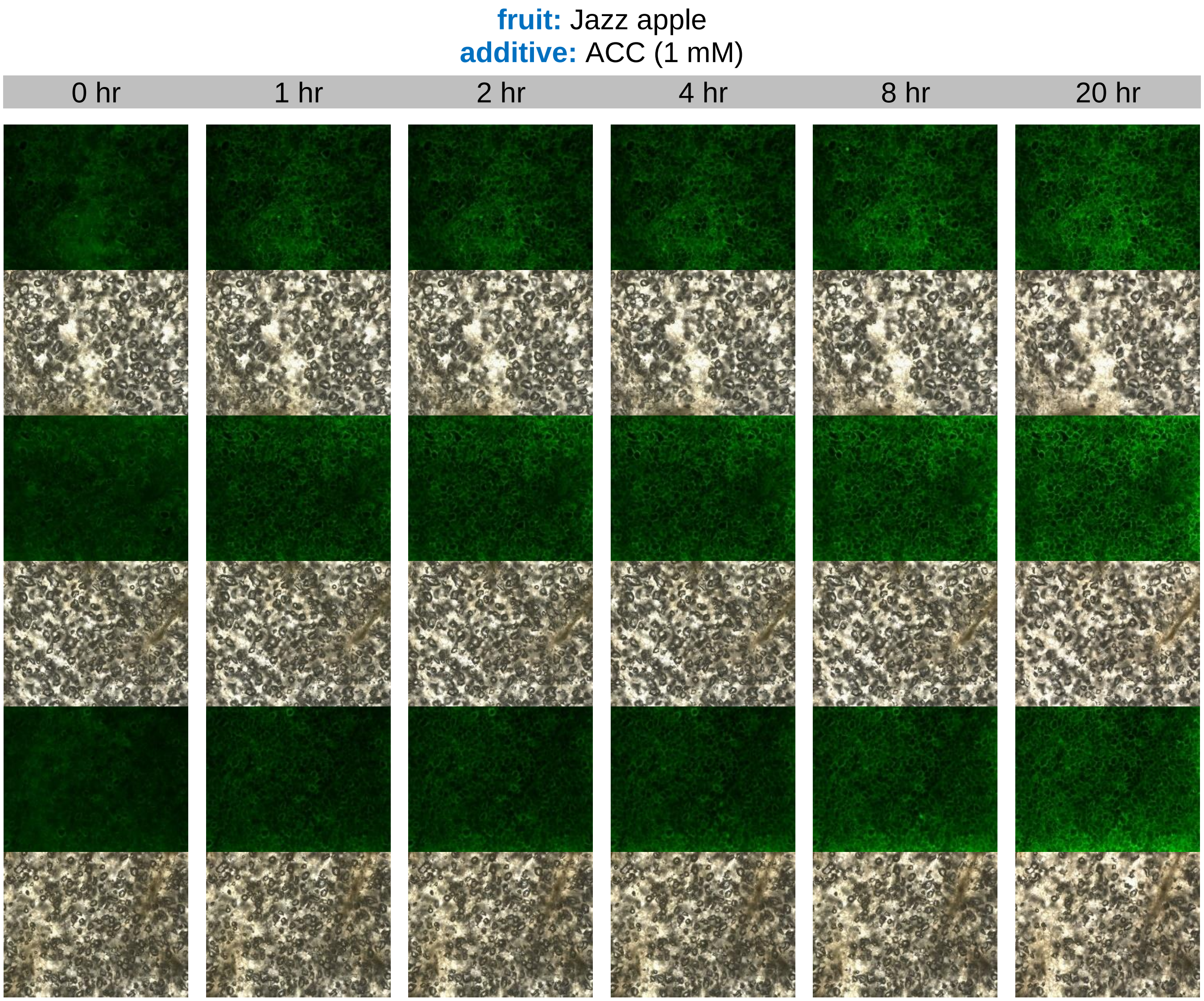

Supplementary Figure 17. Time-based imaging of Jazz apples with 1 mM ACC supplementation.

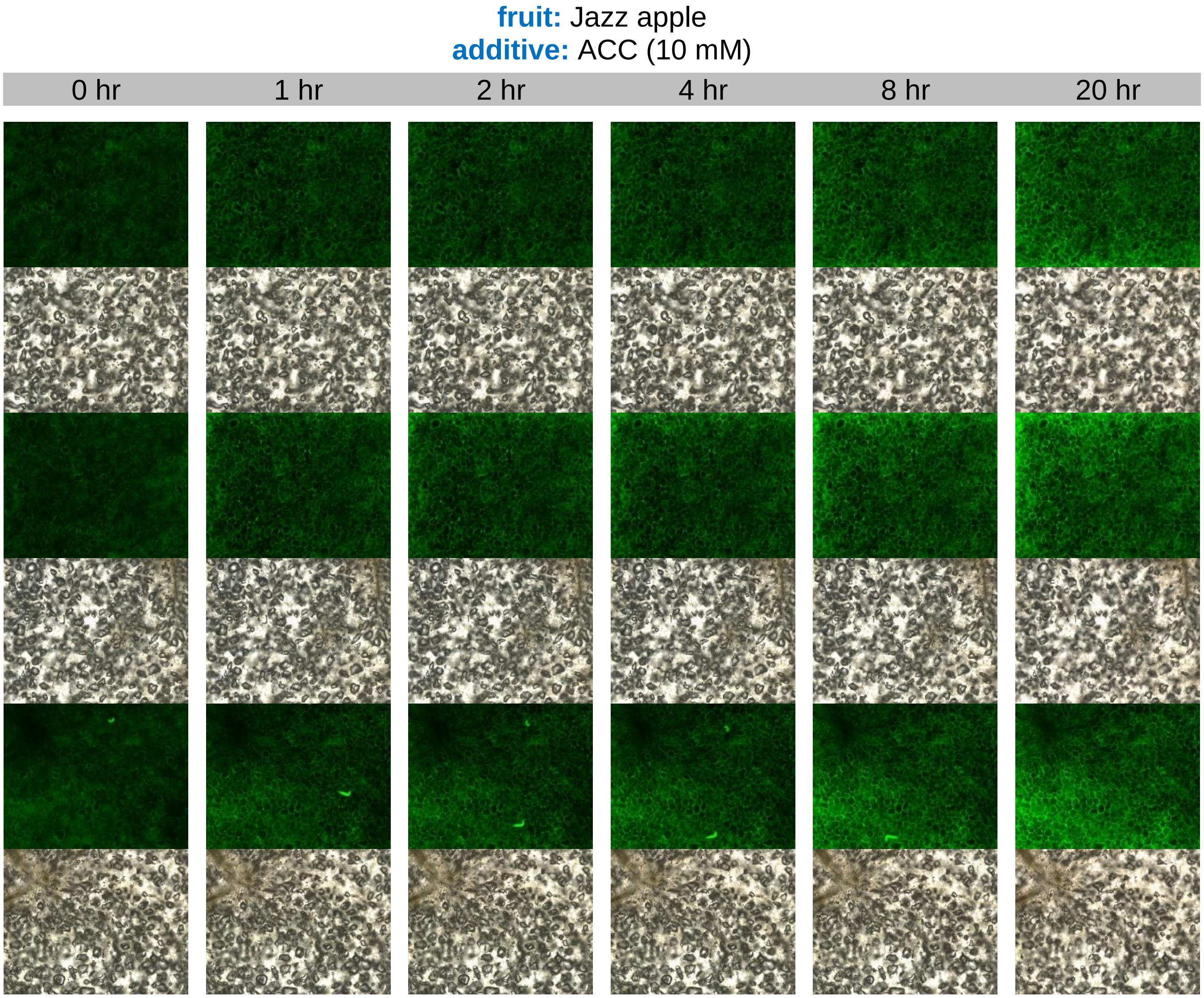

Supplementary Figure 18. Time-based imaging of Jazz apples with 10 mM ACC supplementation.

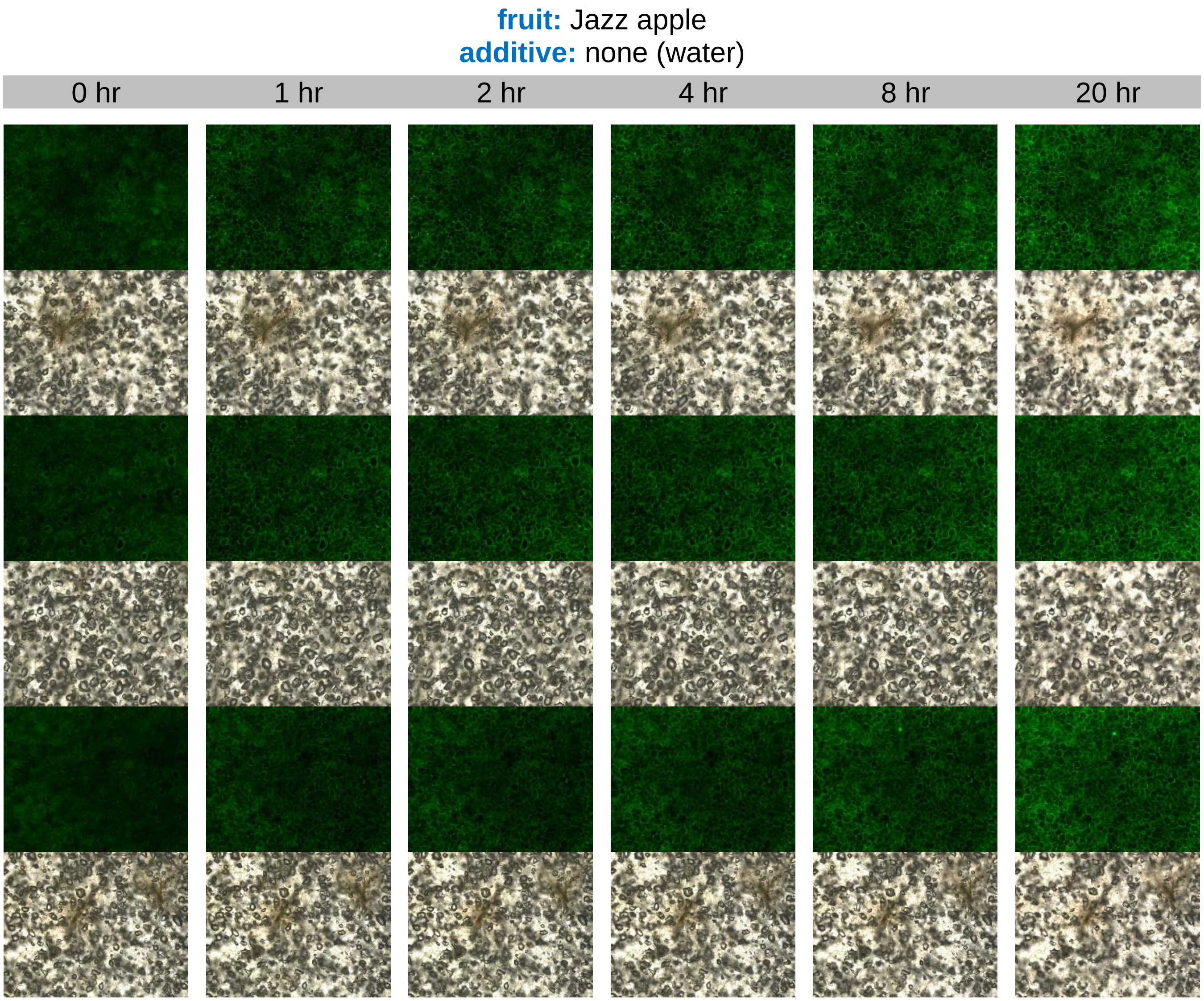

Supplementary Figure 19. Time-based imaging of Jazz apples with no supplementation.

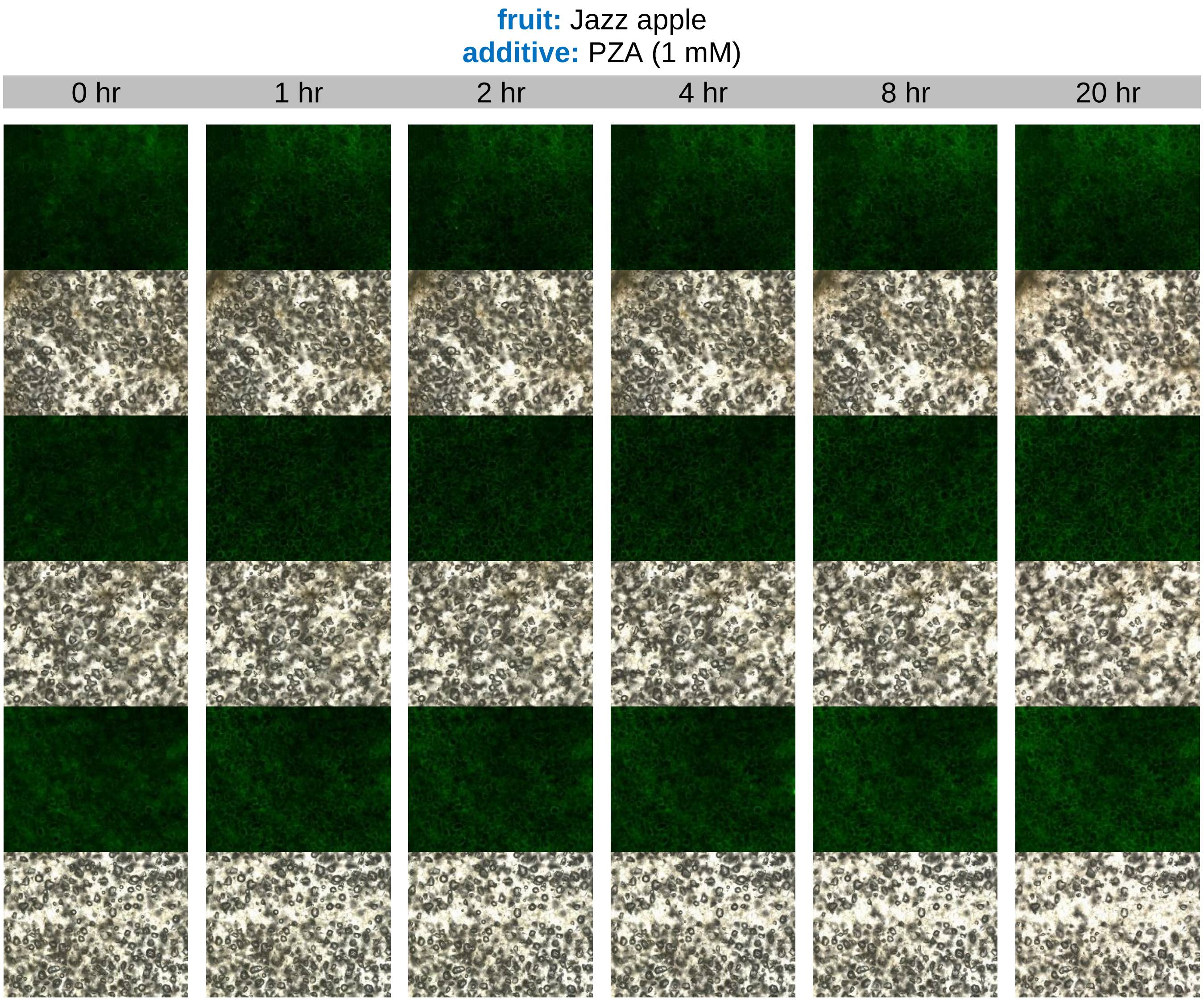

Supplementary Figure 20. Time-based imaging of Jazz apples with 1 mM PZA supplementation.

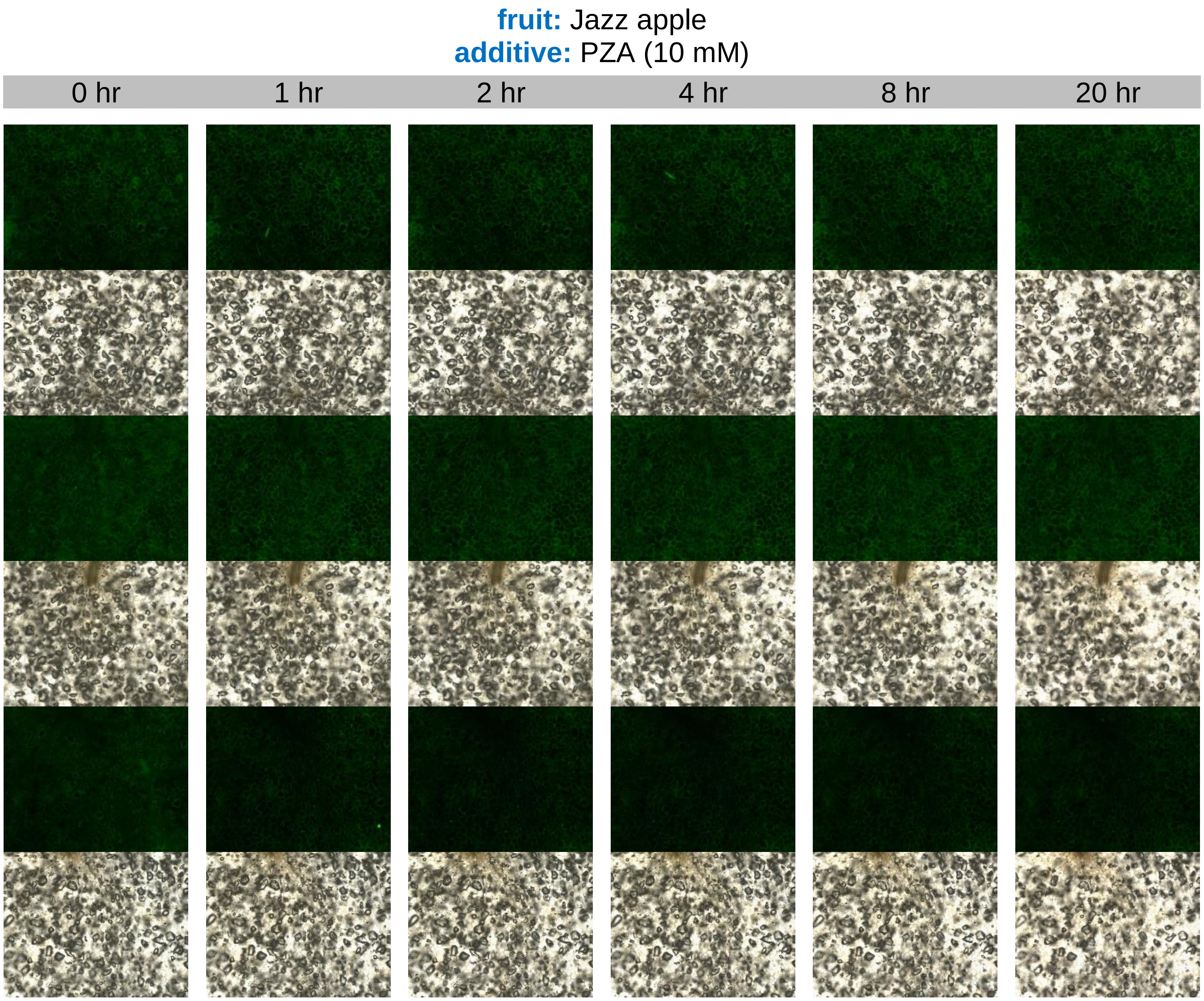

Supplementary Figure 21. Time-based imaging of Jazz apples with 10 mM PZA supplementation.

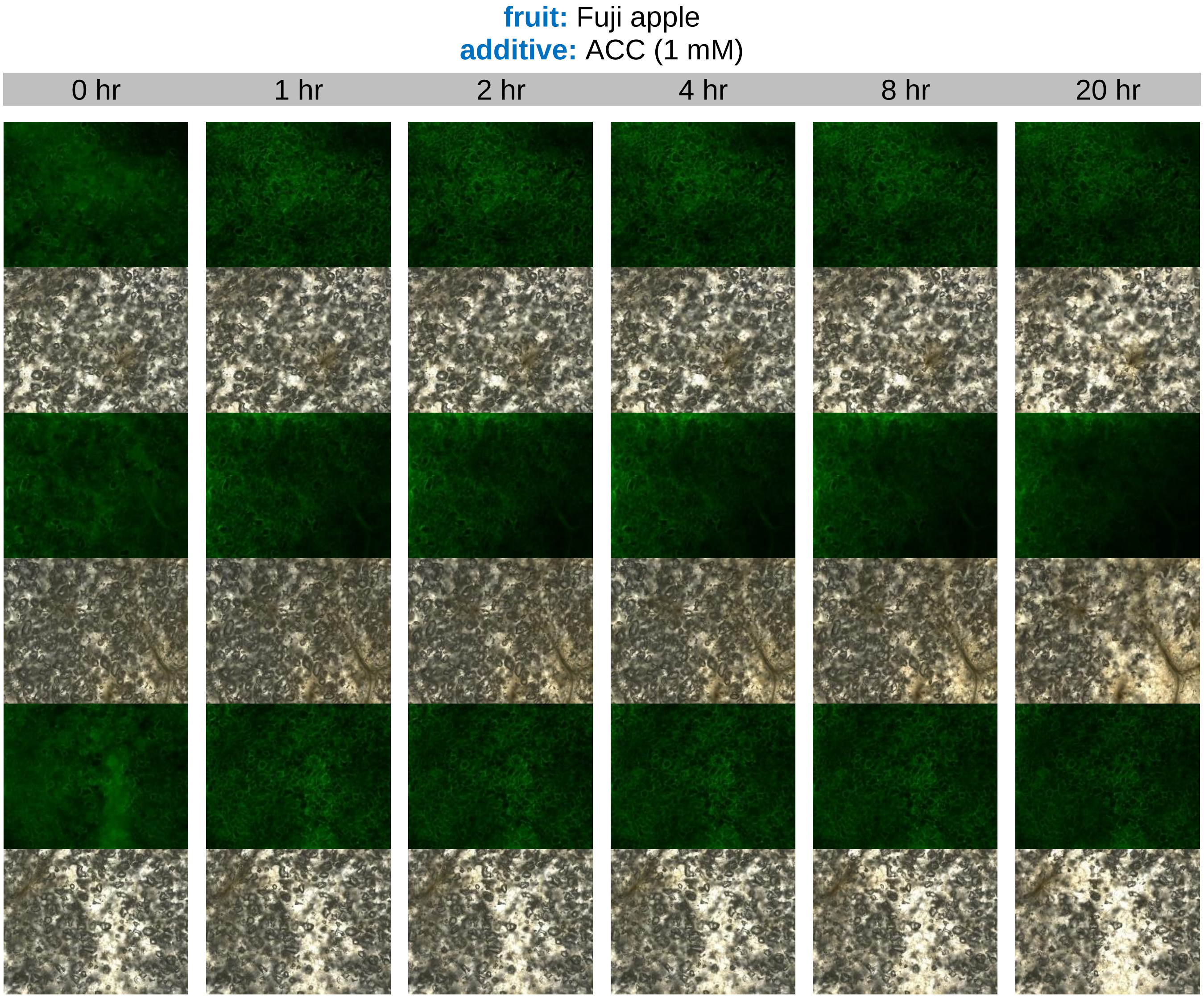

Supplementary Figure 22. Time-based imaging of Fuji apples with 1 mM ACC supplementation.

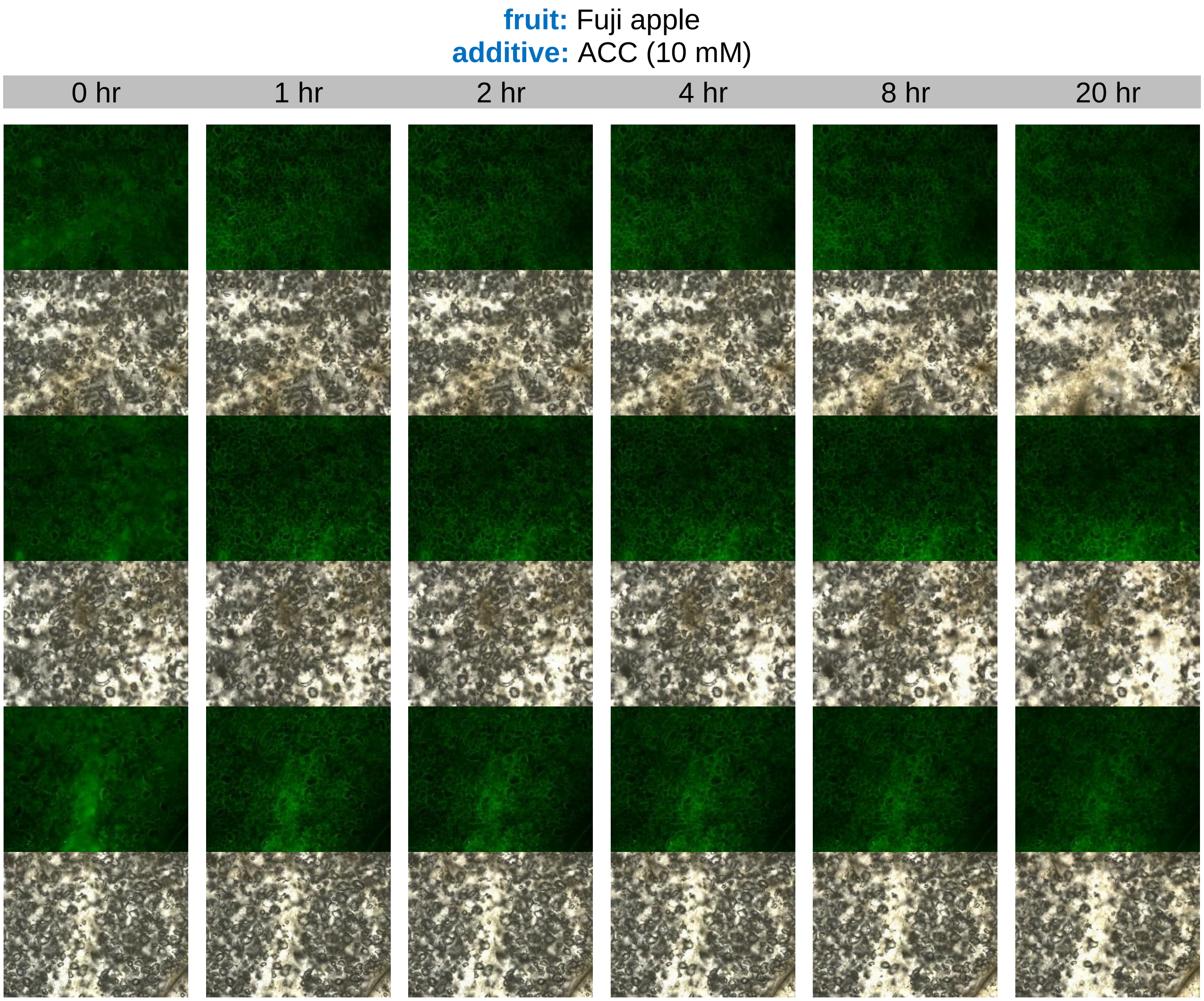

Supplementary Figure 23. Time-based imaging of Fuji apples with 10 mM ACC supplementation.

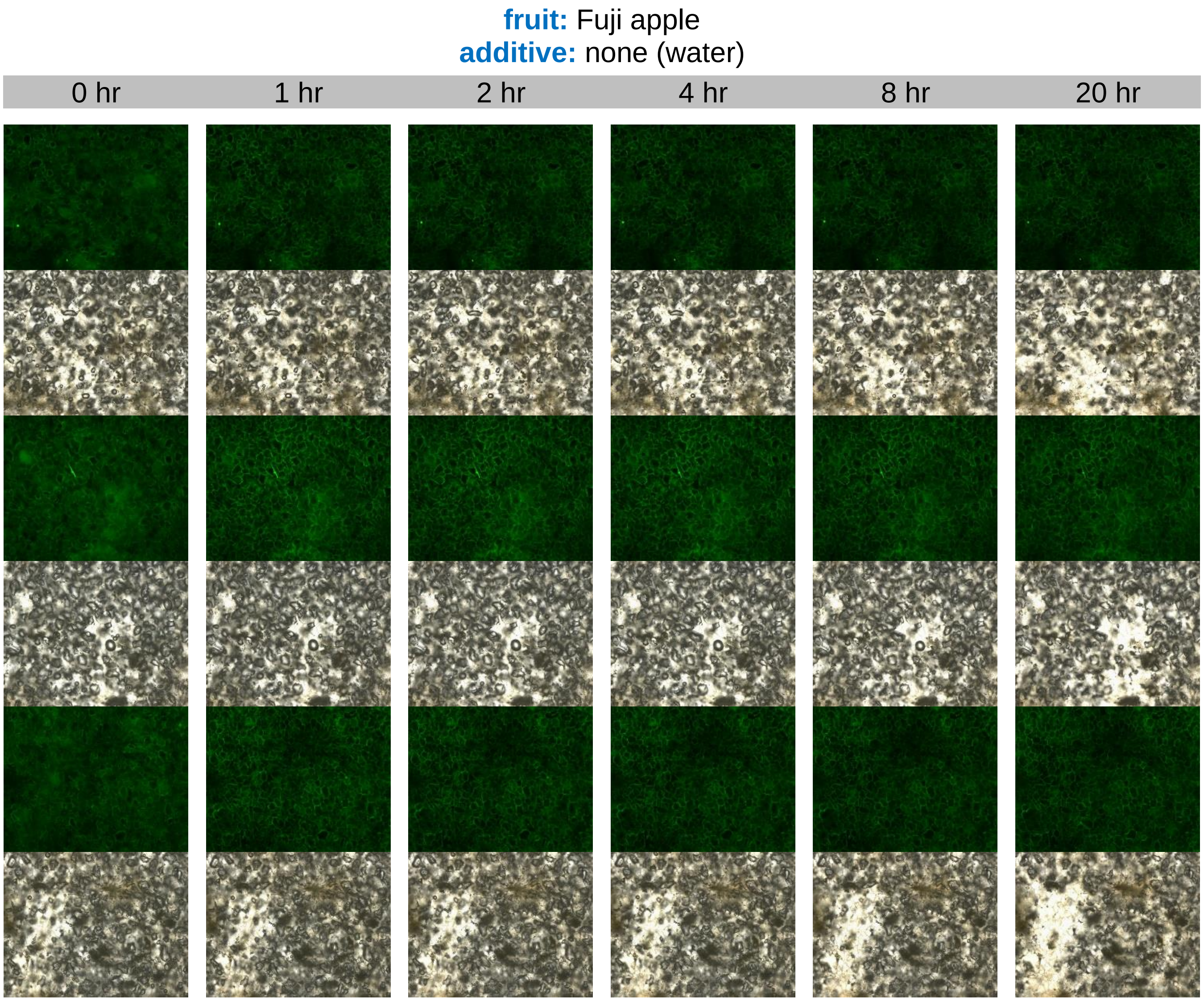

Supplementary Figure 24. Time-based imaging of Fuji apples with no supplementation.

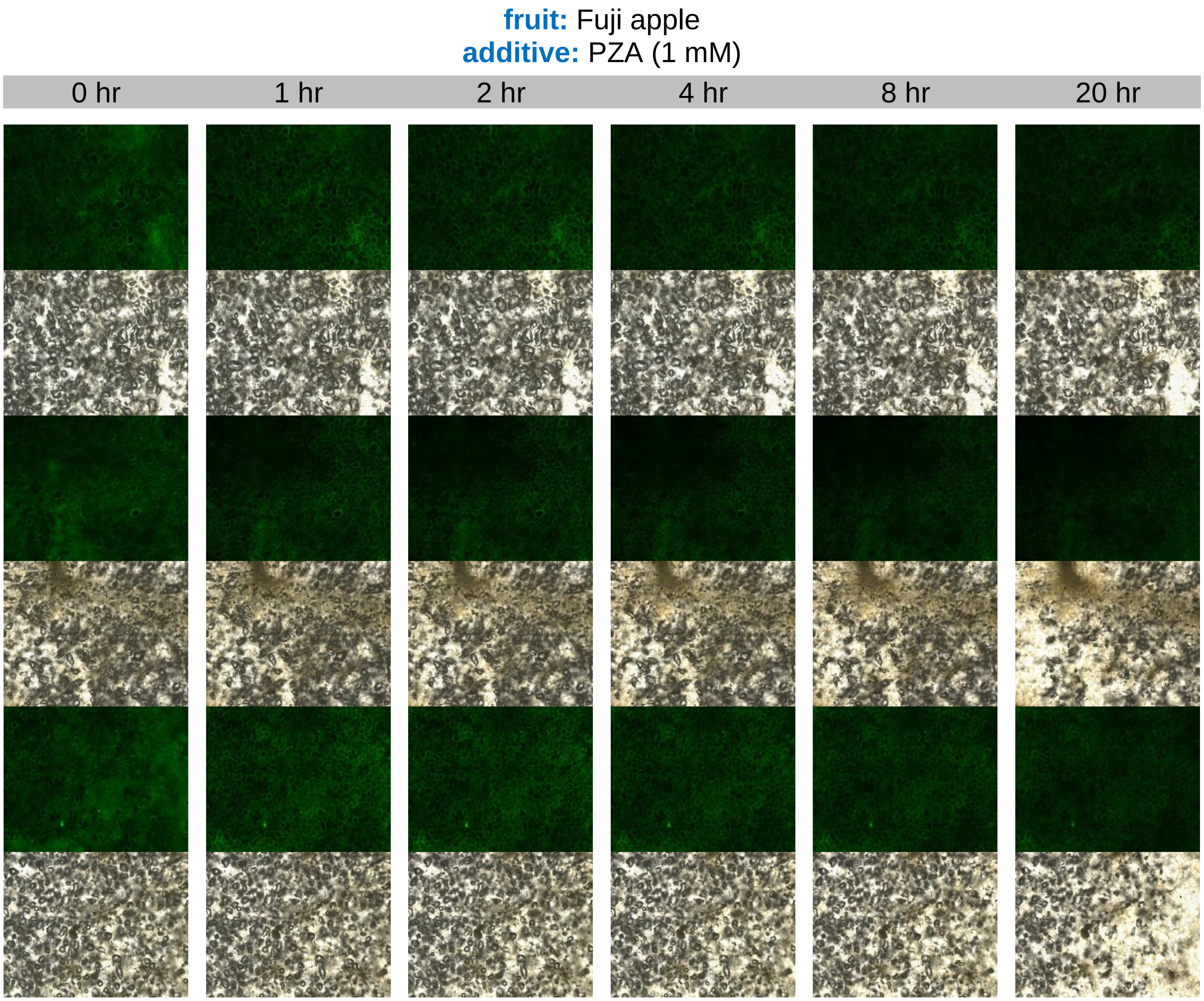

Supplementary Figure 25. Time-based imaging of Fuji apples with 1 mM PZA supplementation.

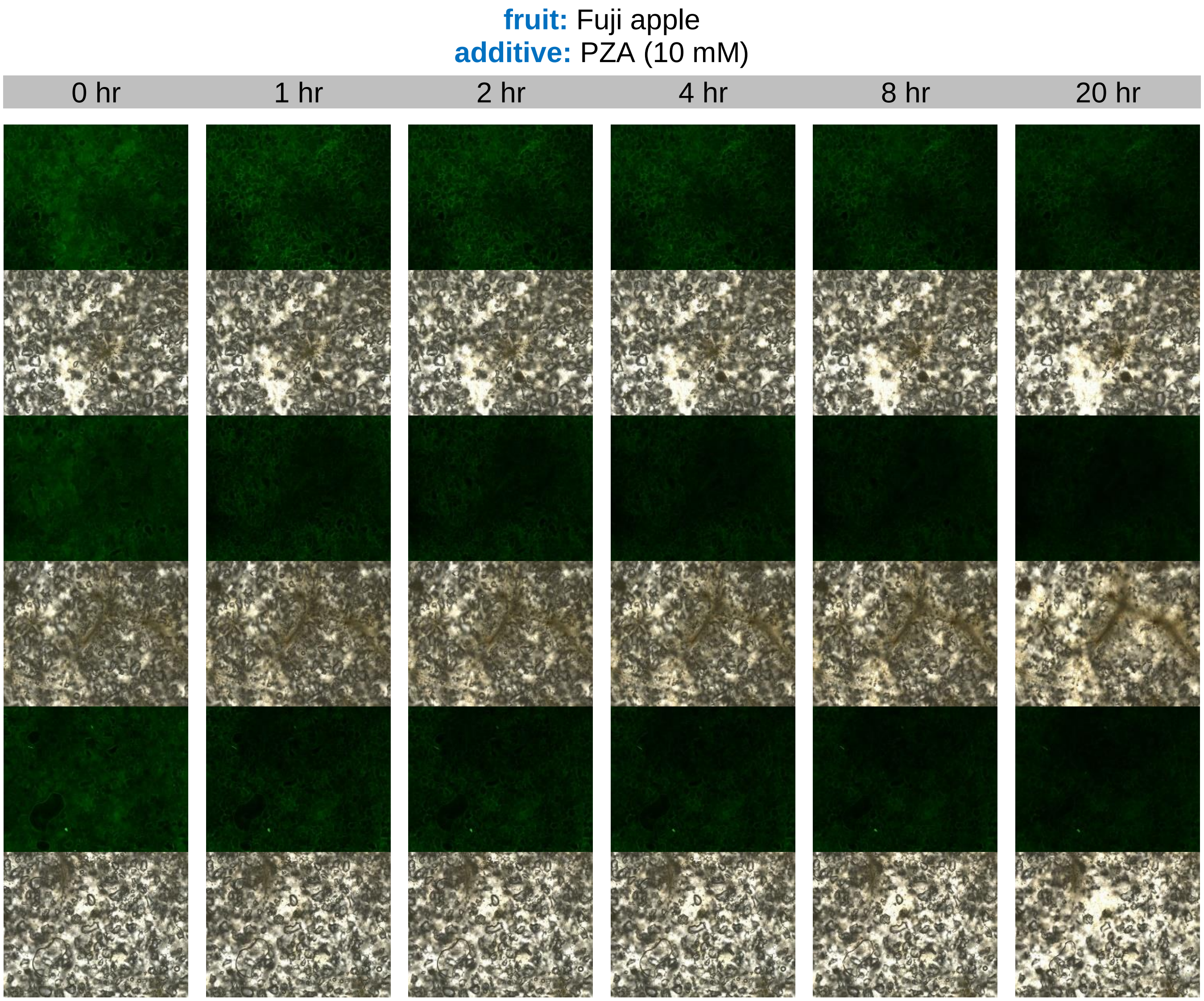

Supplementary Figure 26. Time-based imaging of Fuji apples with 10 mM PZA supplementation.

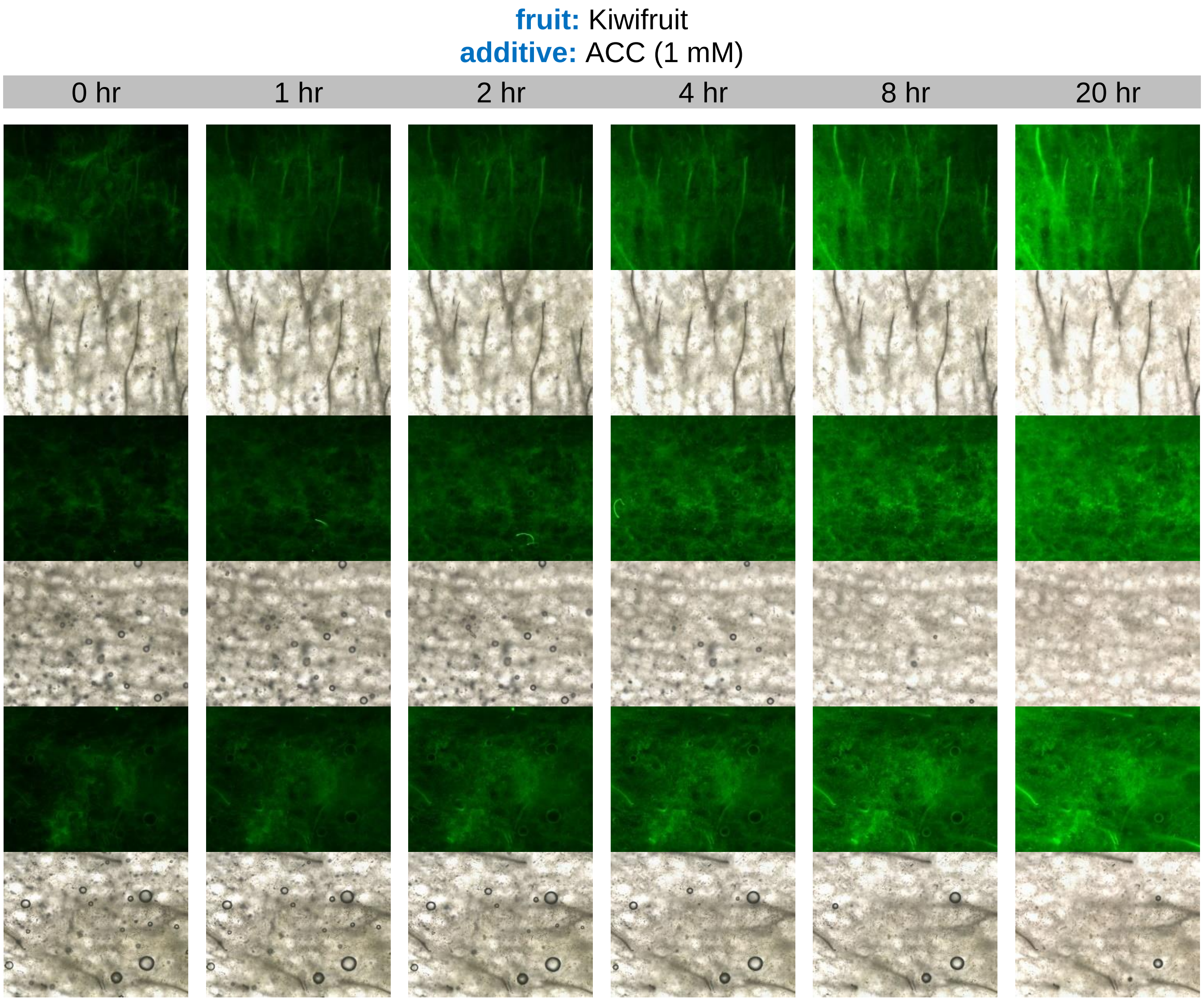

Supplementary Figure 27. Time-based imaging of kiwifruit with 1 mM ACC supplementation.

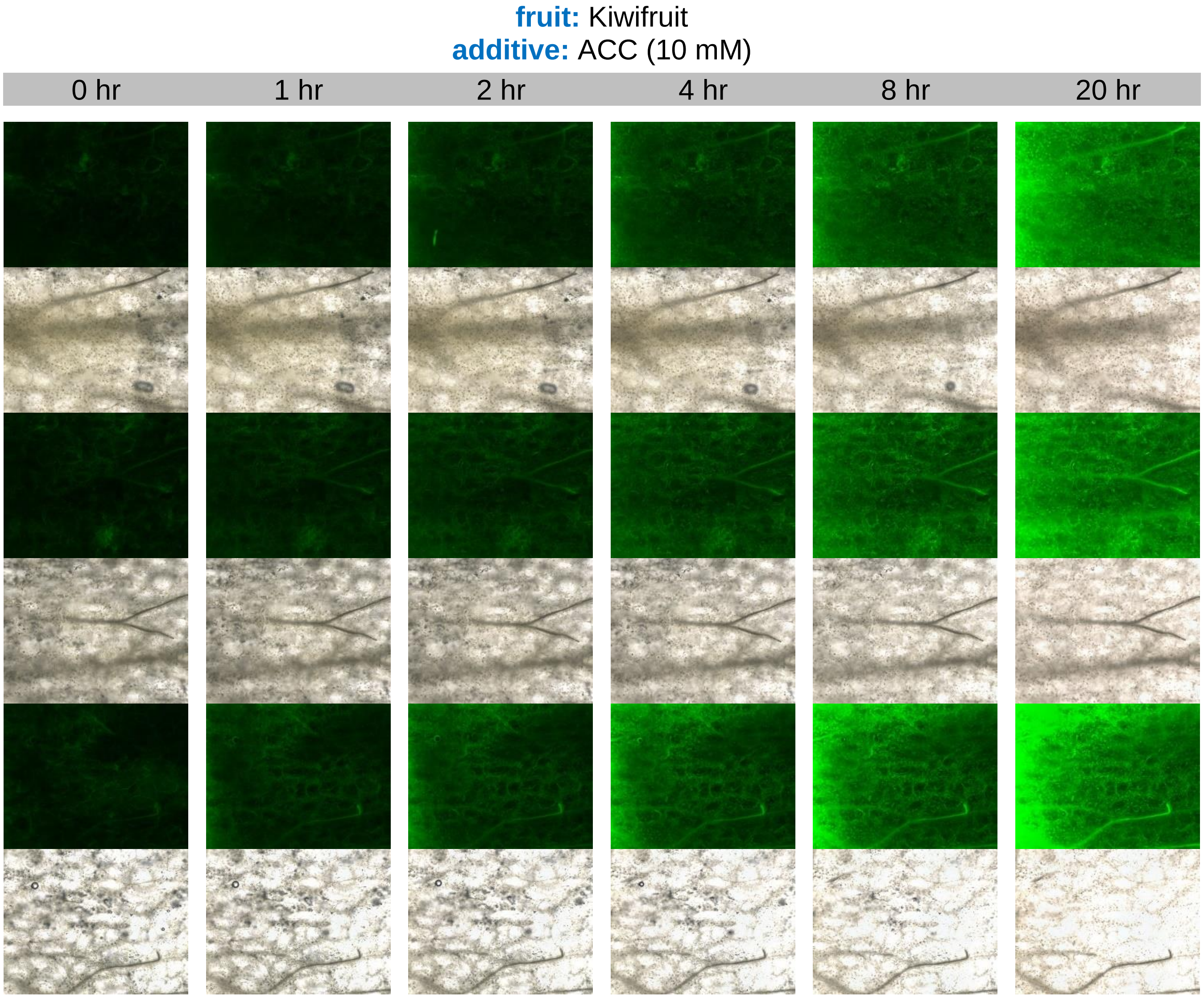

Supplementary Figure 28. Time-based imaging of kiwifruit with 10 mM ACC supplementation.

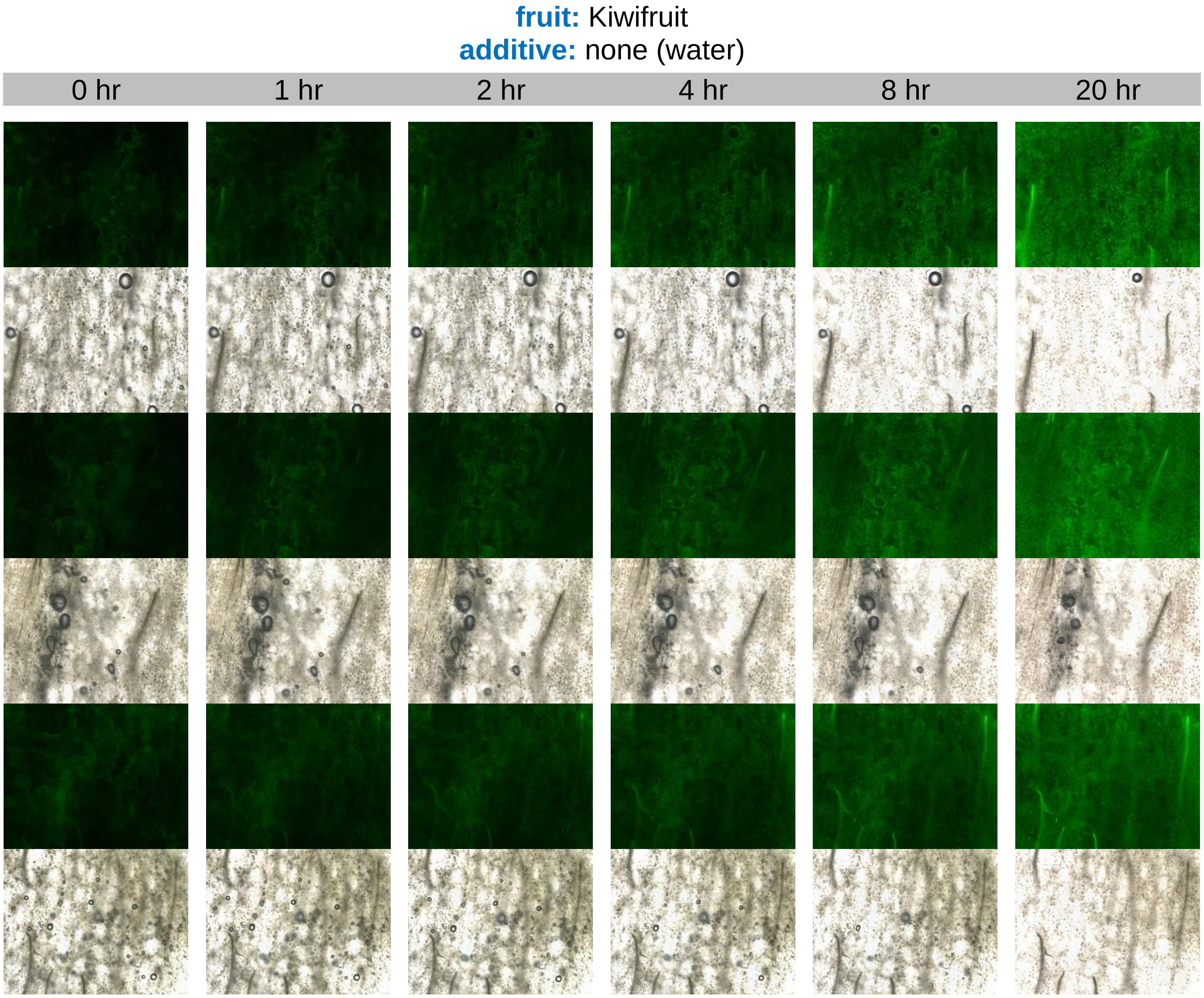

Supplementary Figure 29. Time-based imaging of kiwifruit with no supplementation.

fruit: Kiwifruit
additive: PZA (1 mM)

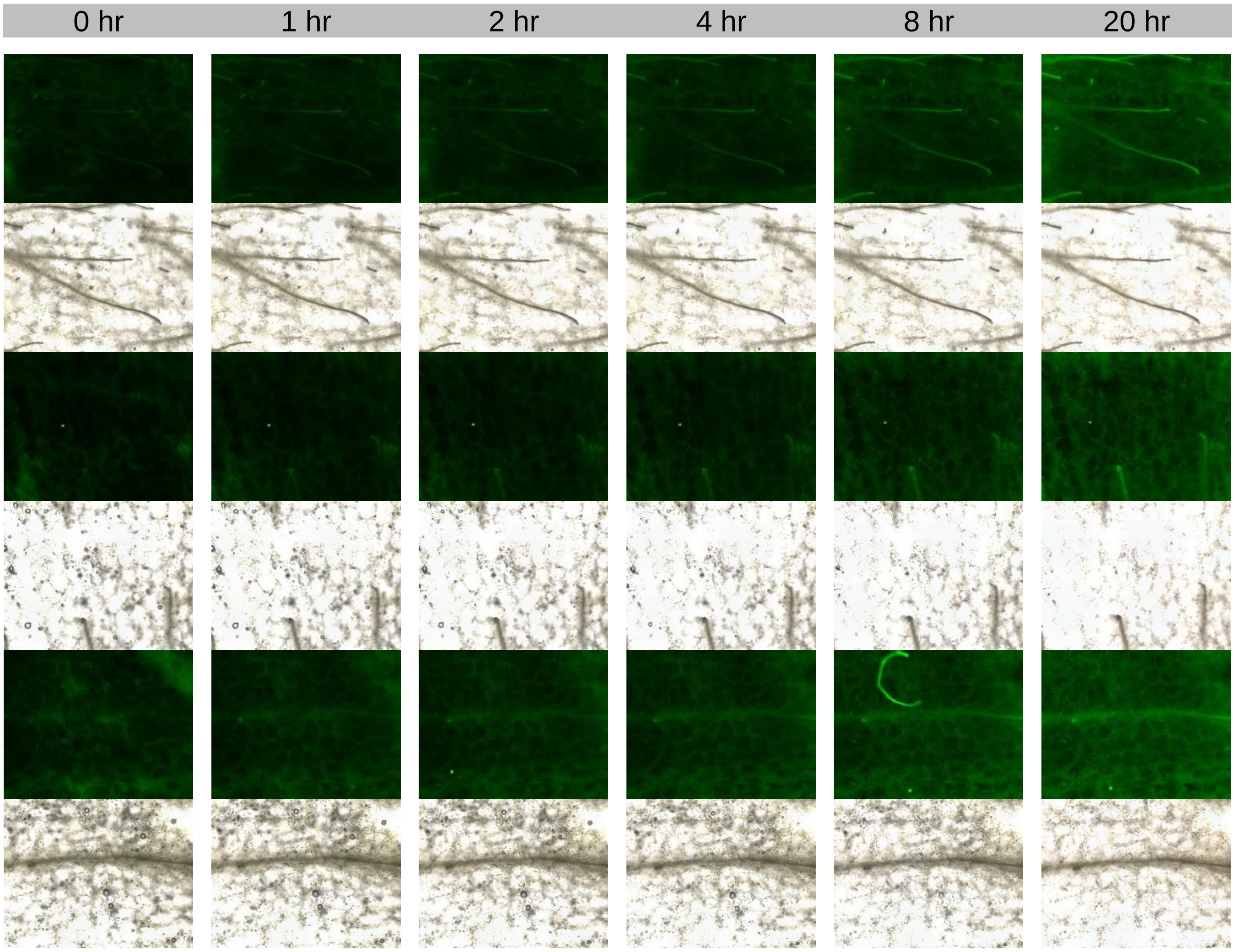

Supplementary Figure 30. Time-based imaging of kiwifruit with 1 mM PZA supplementation.

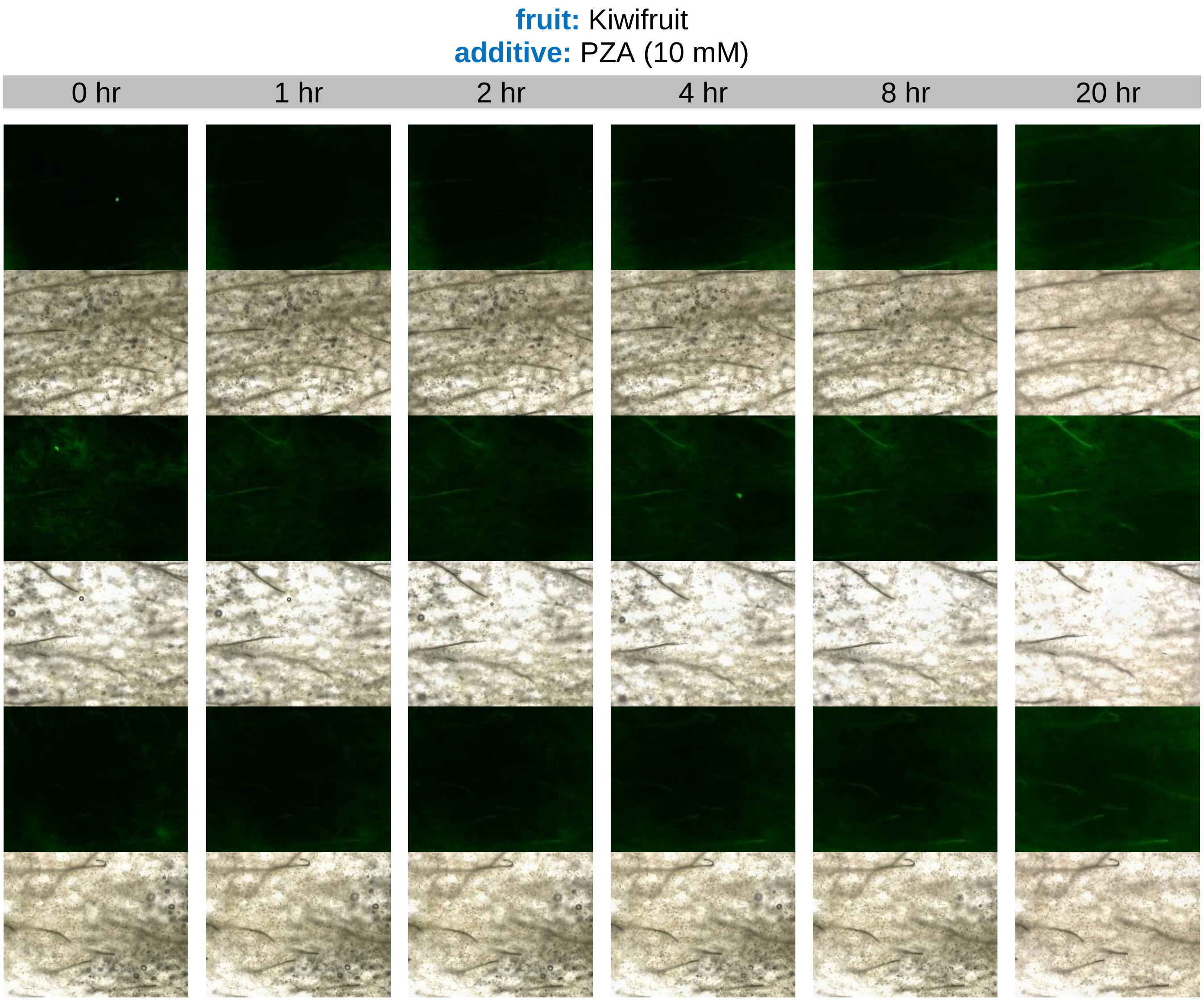

Supplementary Figure 31. Time-based imaging of kiwifruit with 10 mM PZA supplementation.

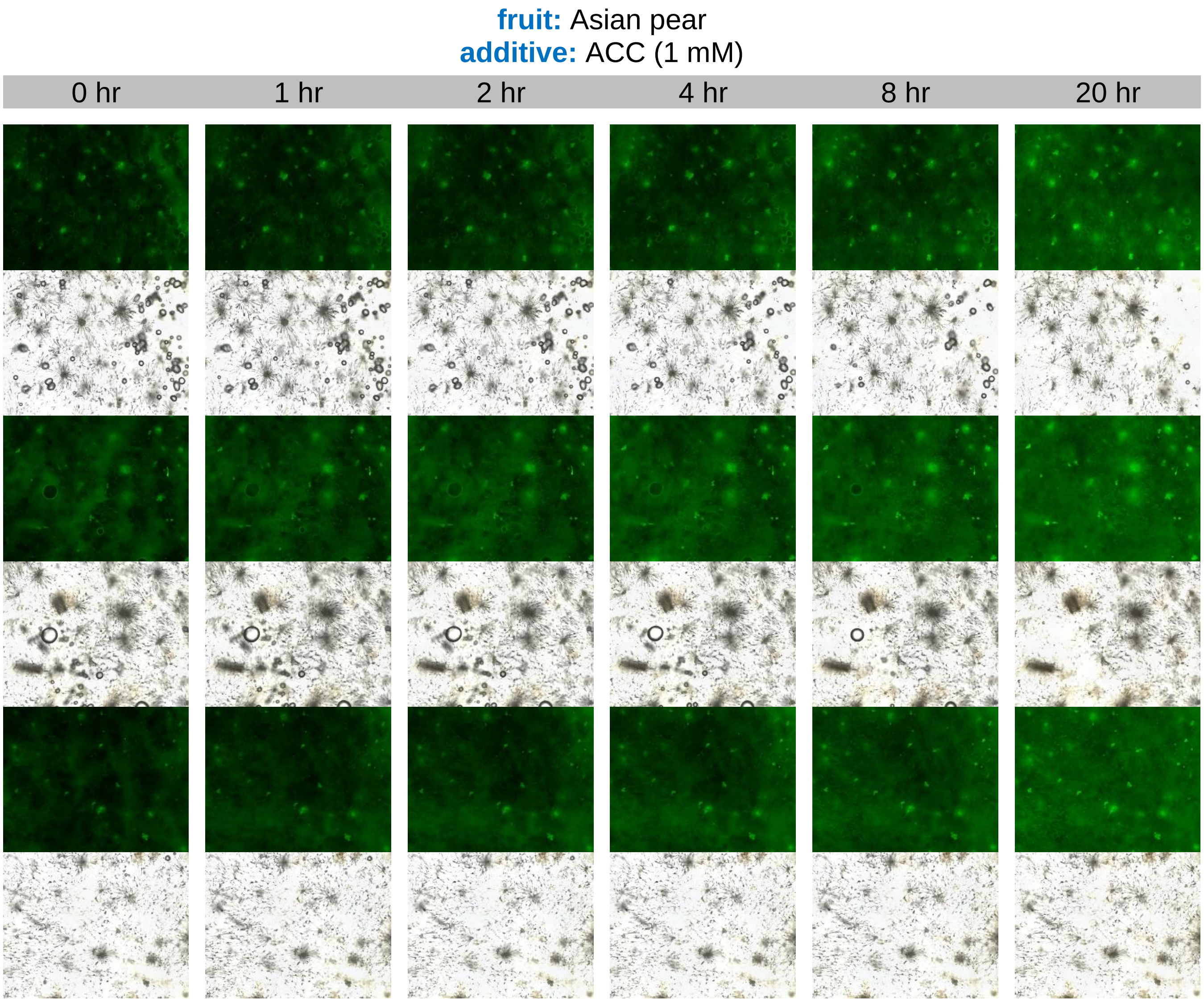

Supplementary Figure 32. Time-based imaging of Asian pear with 1 mM ACC supplementation.

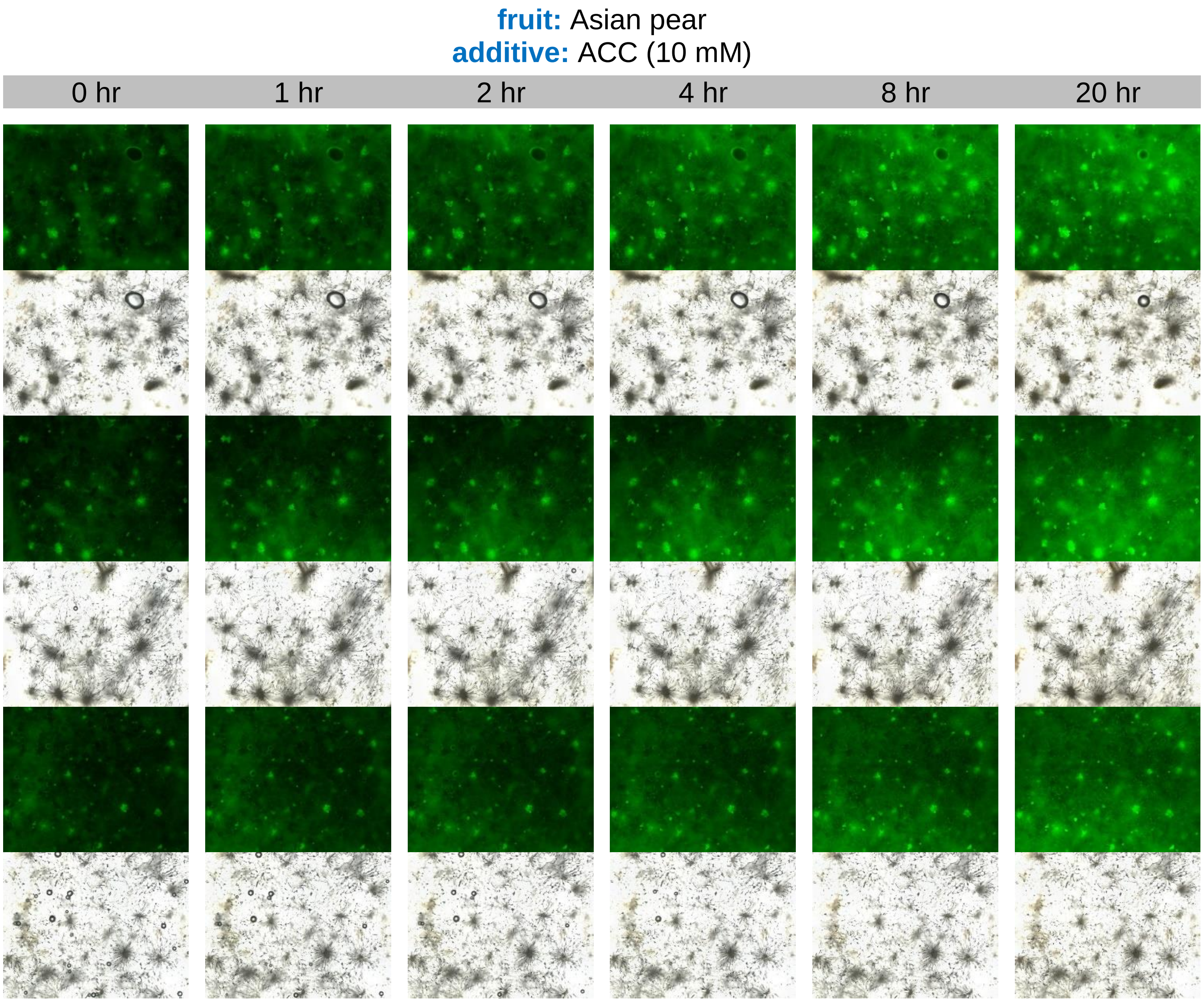

Supplementary Figure 33. Time-based imaging of Asian pear with 10 mM ACC supplementation.

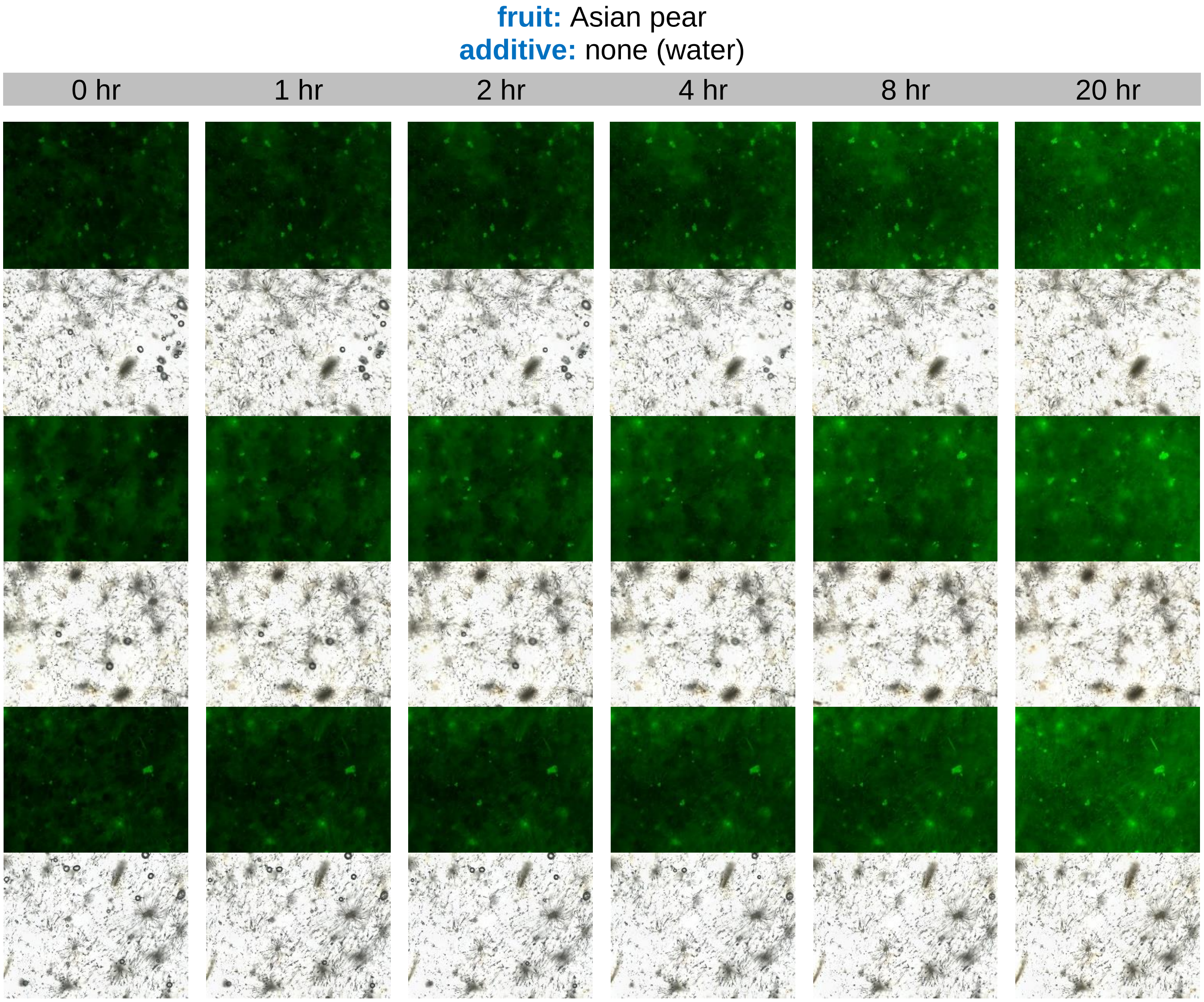

Supplementary Figure 34. Time-based imaging of Asian pear with no supplementation.

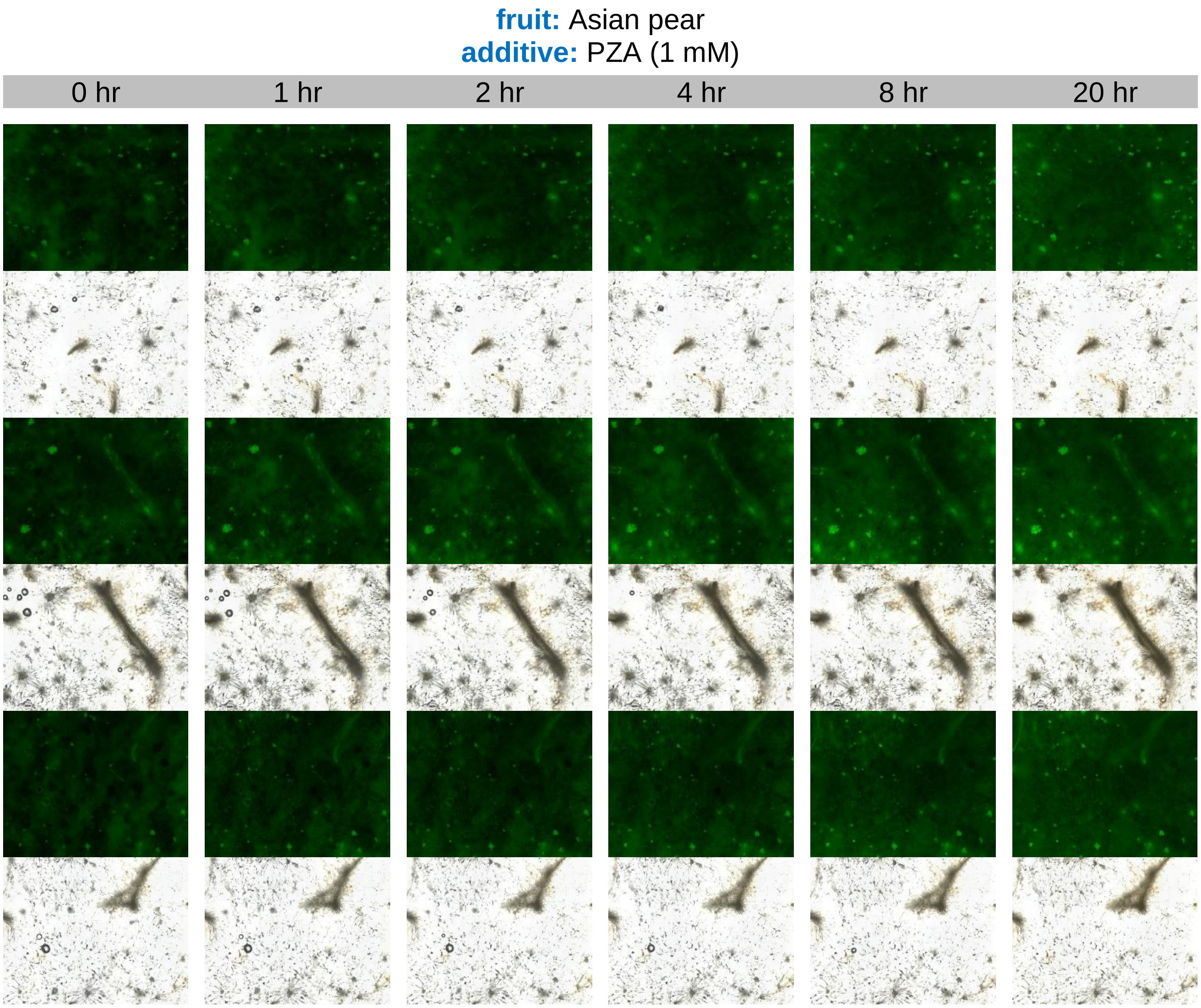

Supplementary Figure 35. Time-based imaging of Asian pear with 1 mM PZA supplementation.

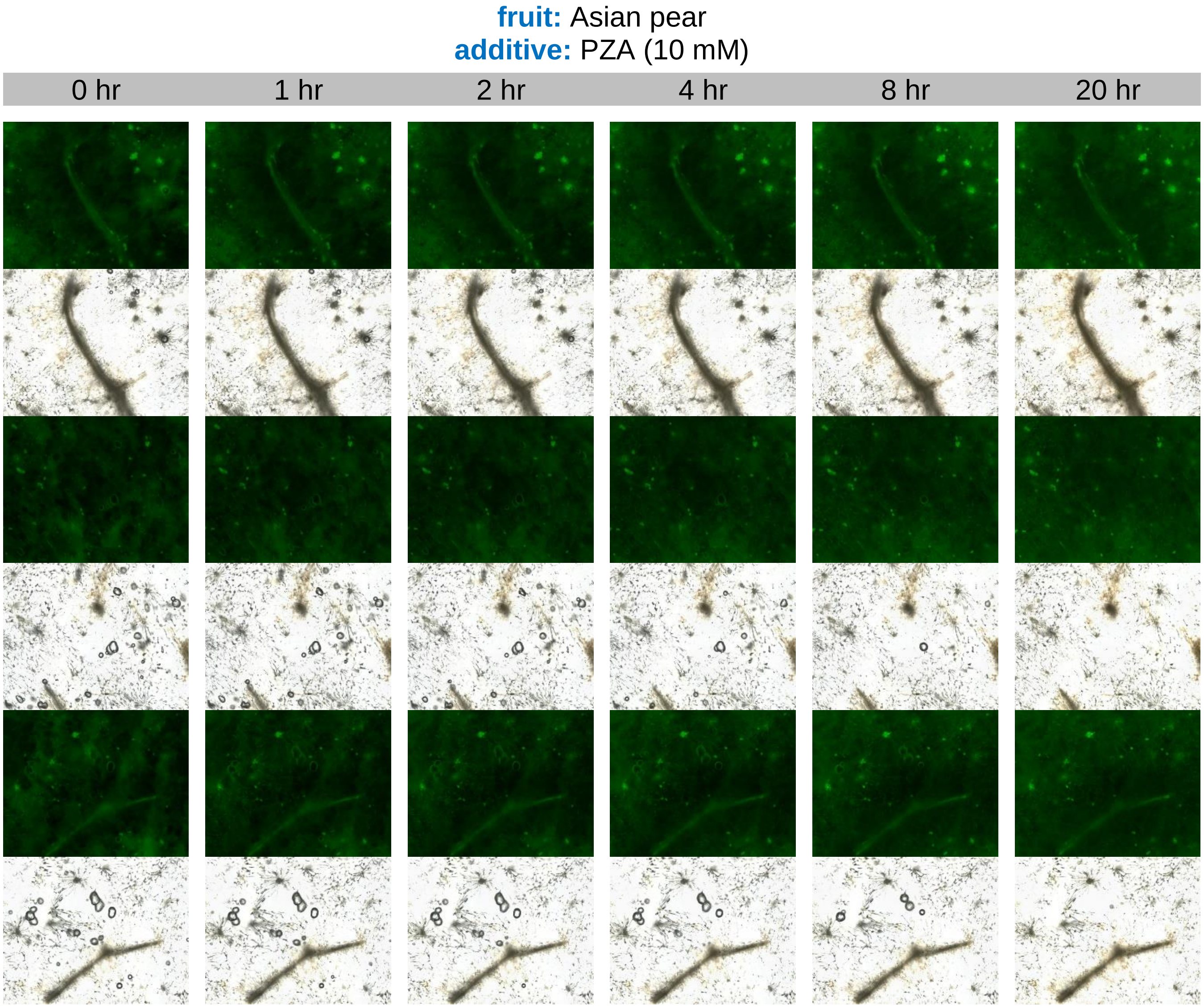

Supplementary Figure 36. Time-based imaging of Asian pear with 10 mM PZA supplementation.

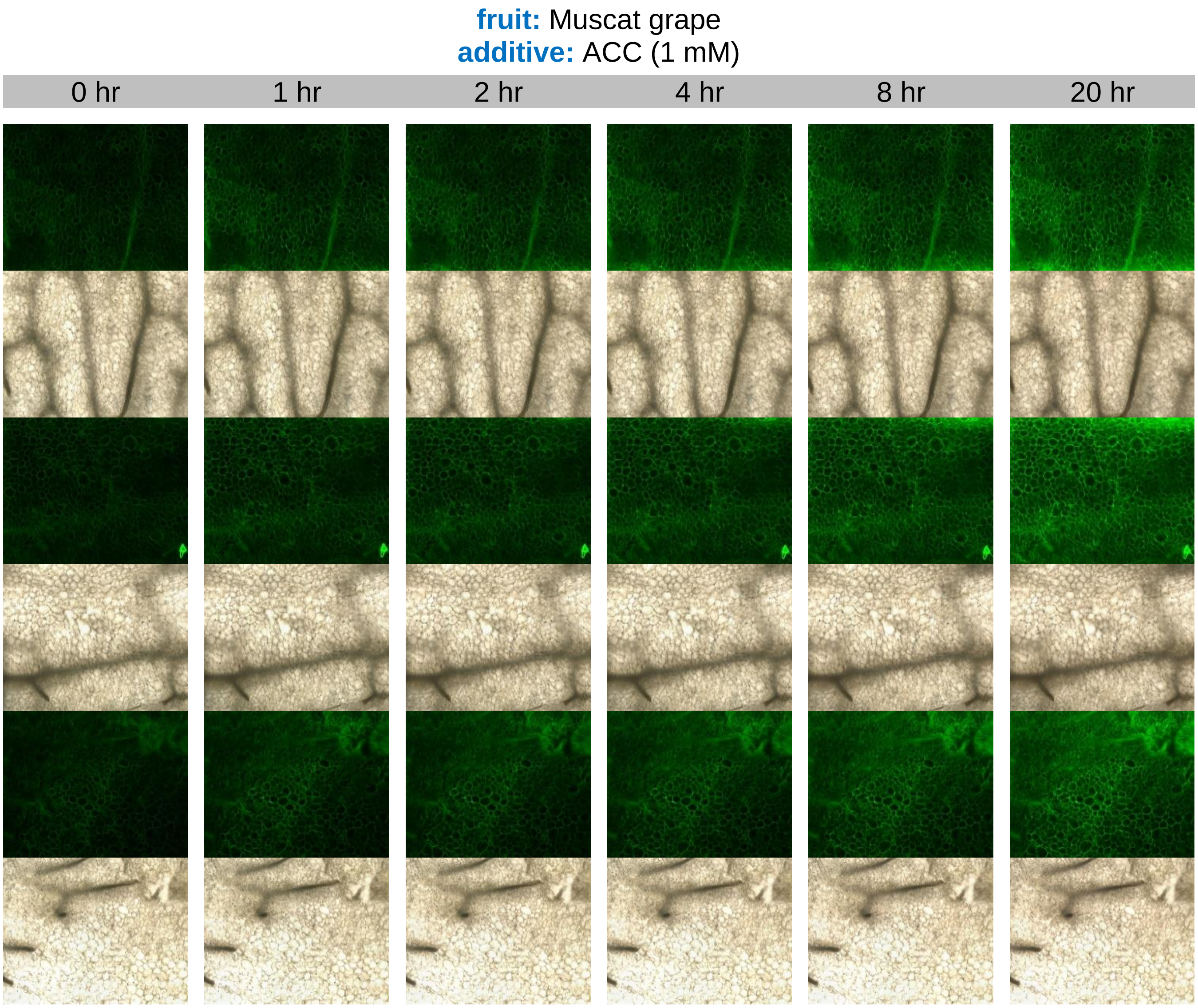

Supplementary Figure 37. Time-based imaging of muscat grape with 1 mM ACC supplementation.

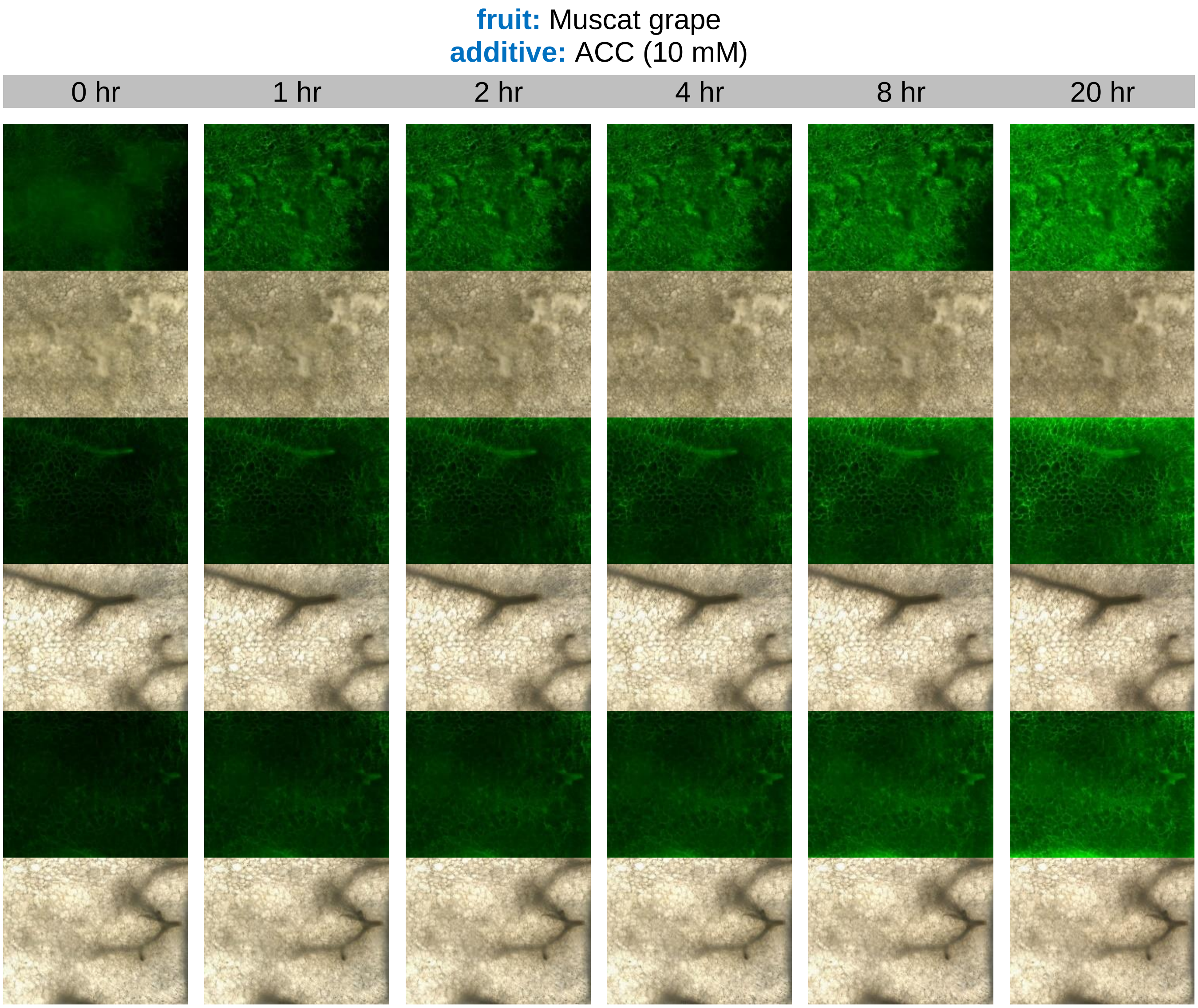

Supplementary Figure 38. Time-based imaging of muscat grape with 10 mM ACC supplementation.

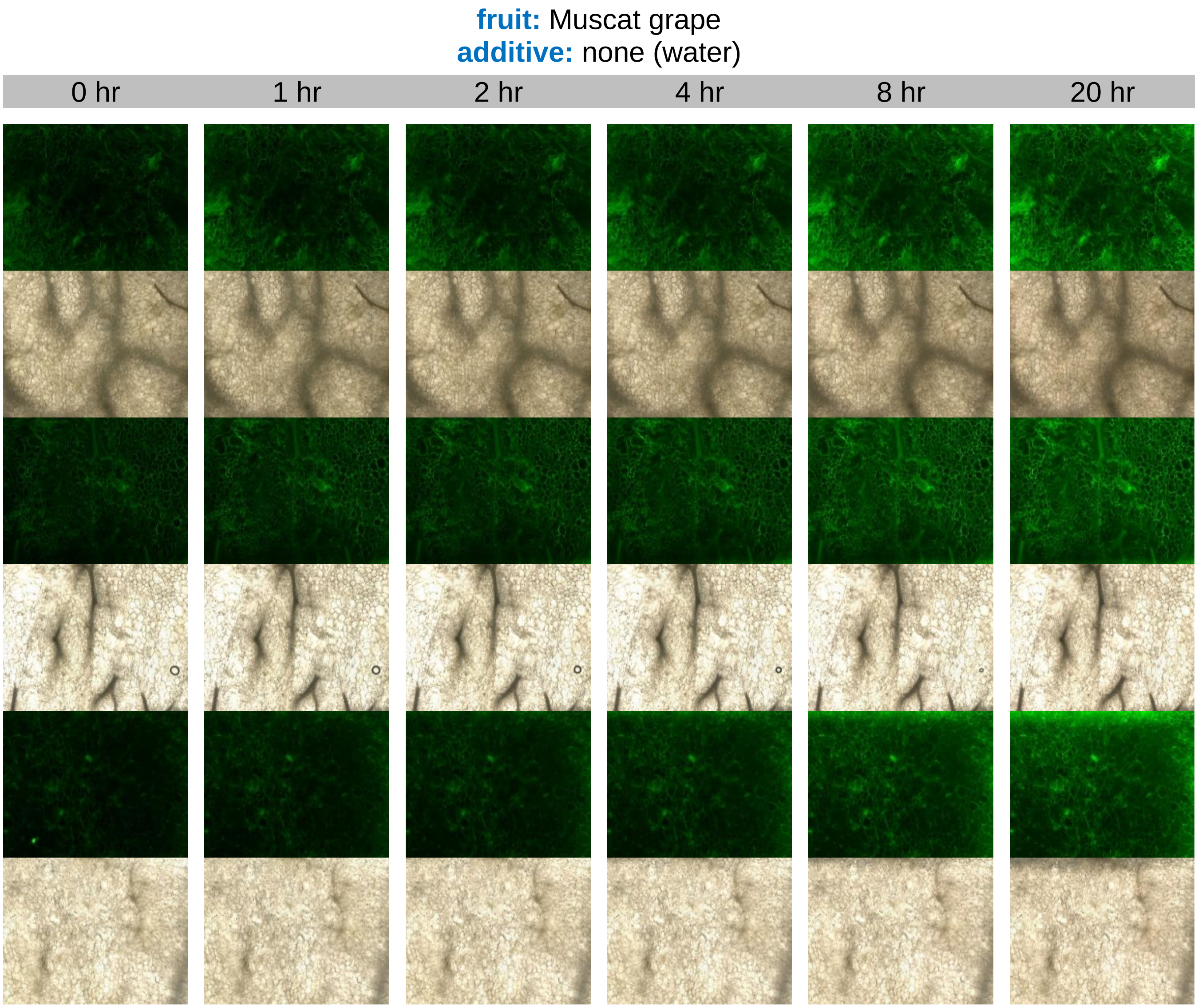

Supplementary Figure 39. Time-based imaging of muscat grape with no supplementation.

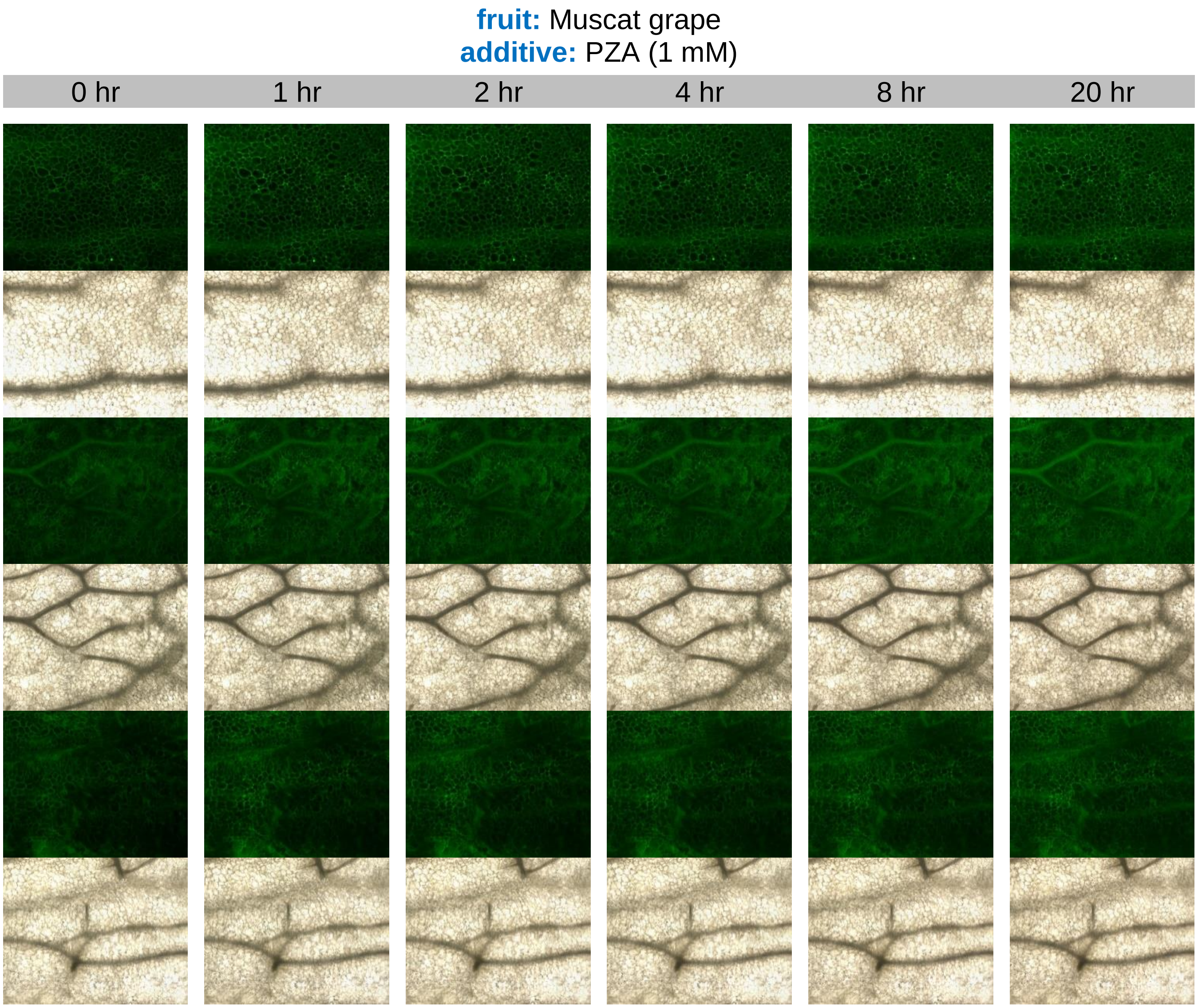

Supplementary Figure 40. Time-based imaging of muscat grape with 1 mM PZA supplementation.

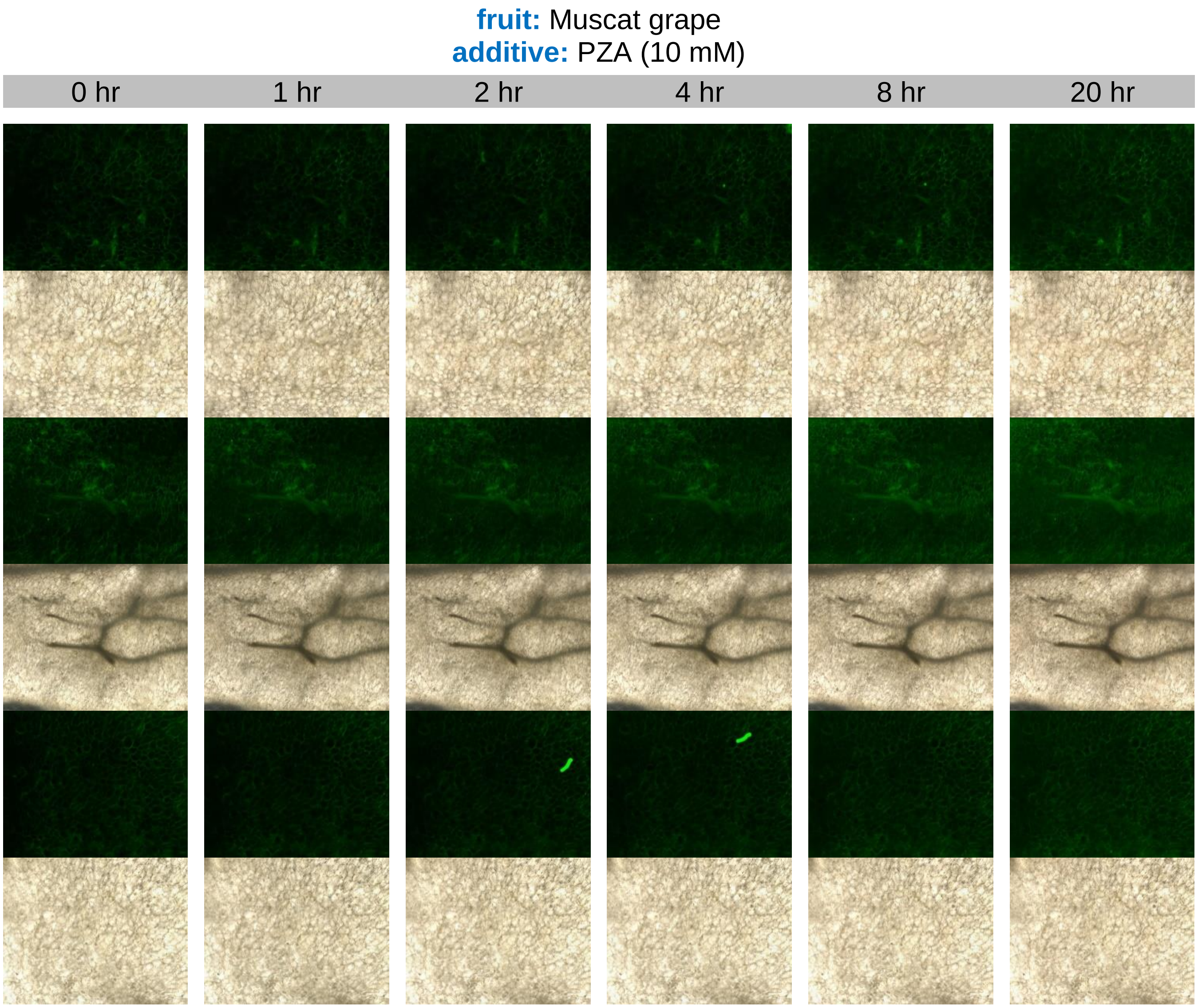

Supplementary Figure 41. Time-based imaging of muscat grape with 10 mM PZA supplementation.

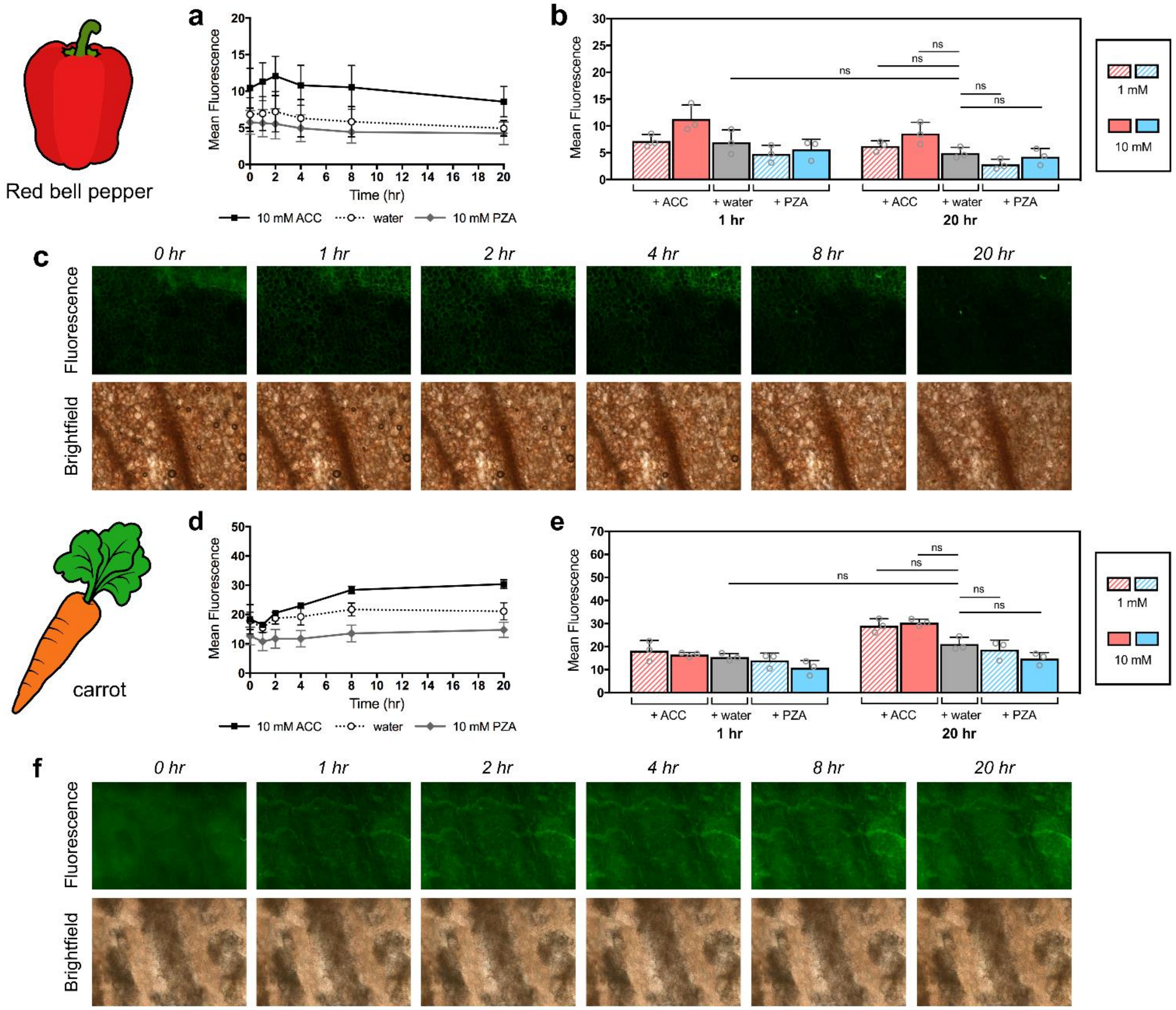

Supplementary Figure 42. Time-dependent detection of ethylene in vegetables. Fluorescence was monitored over time under various conditions (ACC or PZA) for red bell peppers (**a**) and carrots (**d**). A summary and statistical comparison between the 1 hr and 20 hr timepoints for each vegetable are shown (**b,e**), as well as samples images (water-control) over various timepoints (**c,f**). All values were obtained in triplicate. Statistical analysis was performed using a one-way ANOVA with Tukey's multiple comparisons test. *P<0.03, **P<0.002, ***P<0.0002, ****P<0.0001, ns = not significant. Source data are provided as a Source Data file

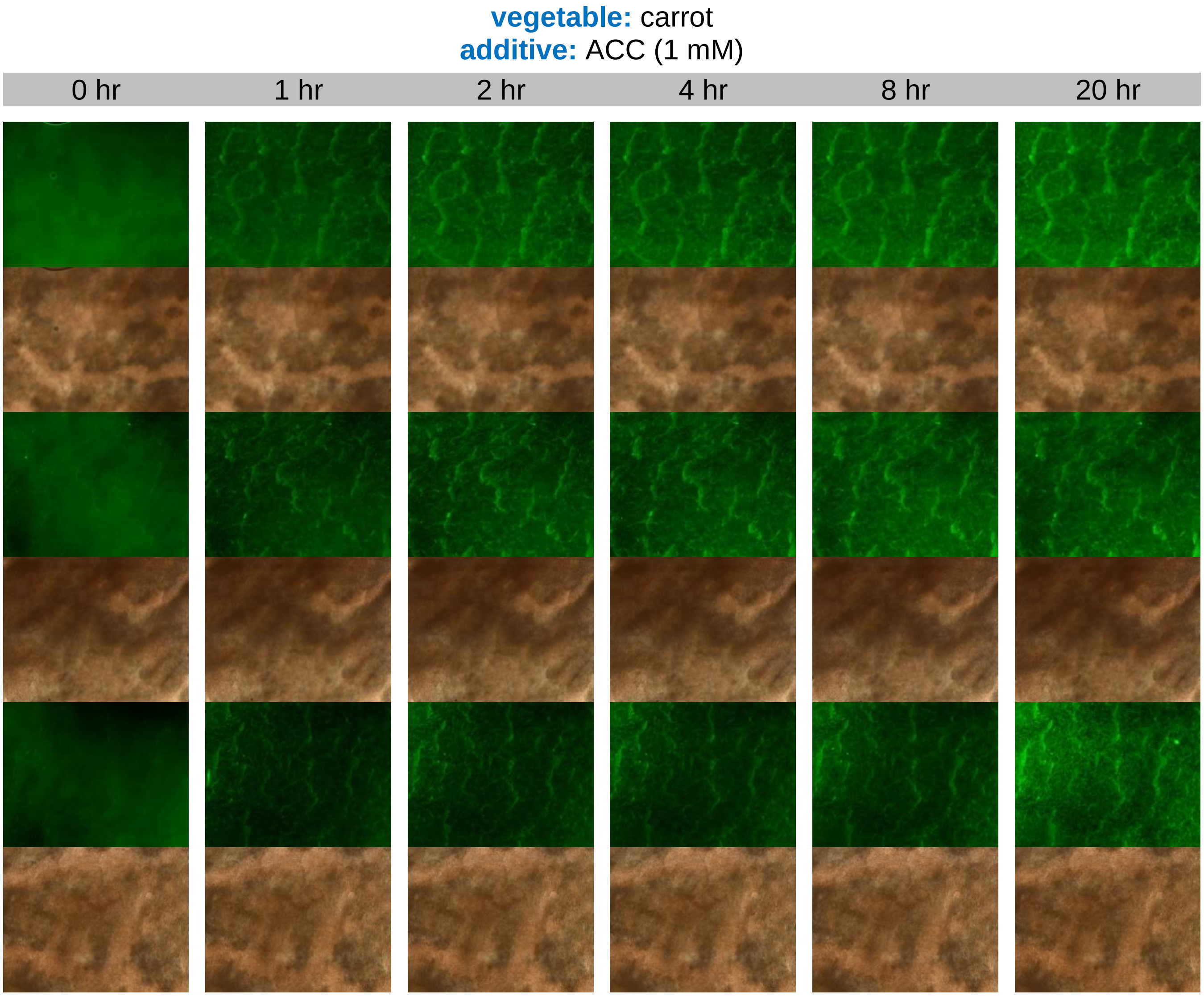

Supplementary Figure 43. Time-based imaging of carrot with 1 mM ACC supplementation.

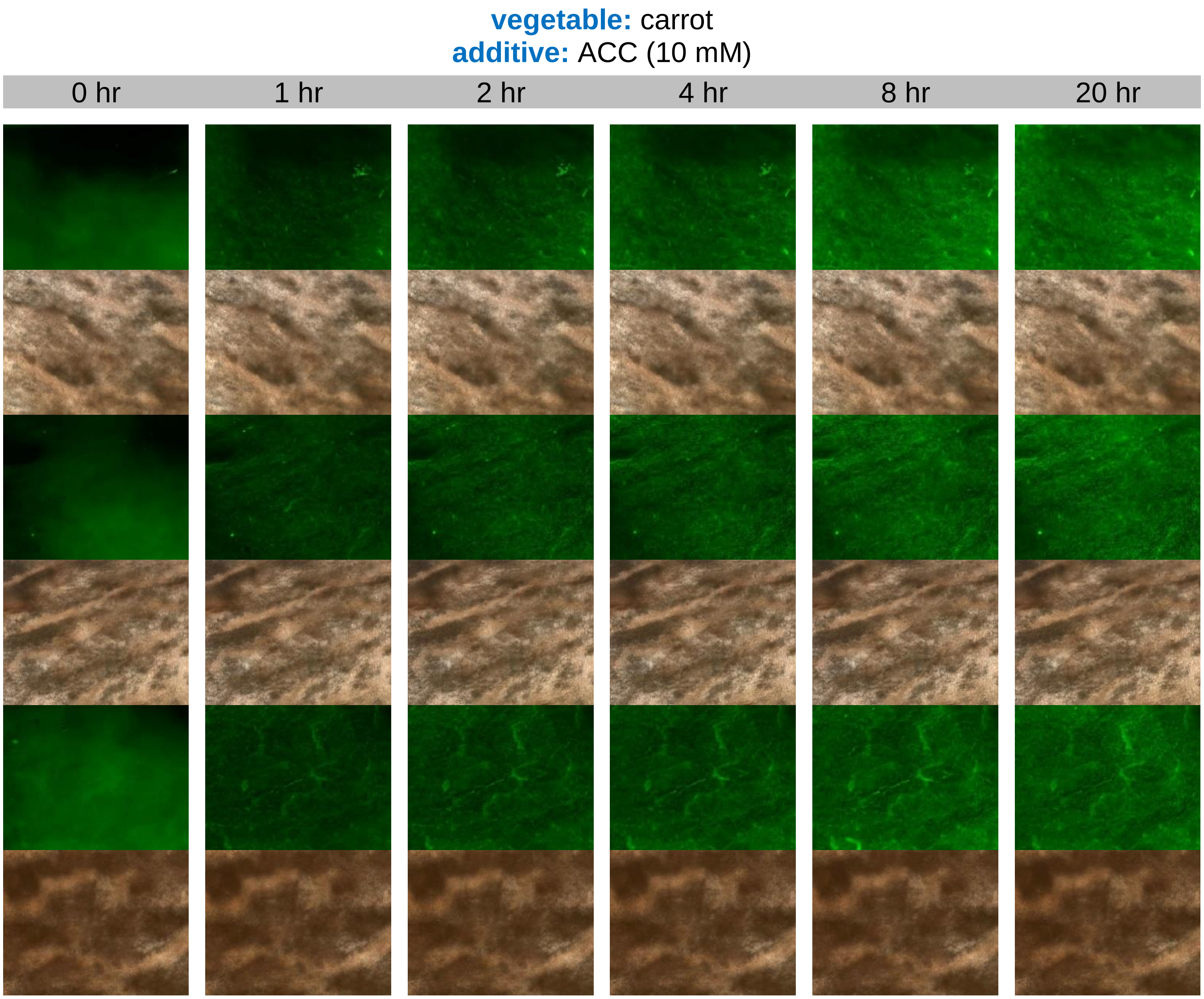

Supplementary Figure 44. Time-based imaging of carrot with 10 mM ACC supplementation.

vegetable: carrot
additive: none (water)

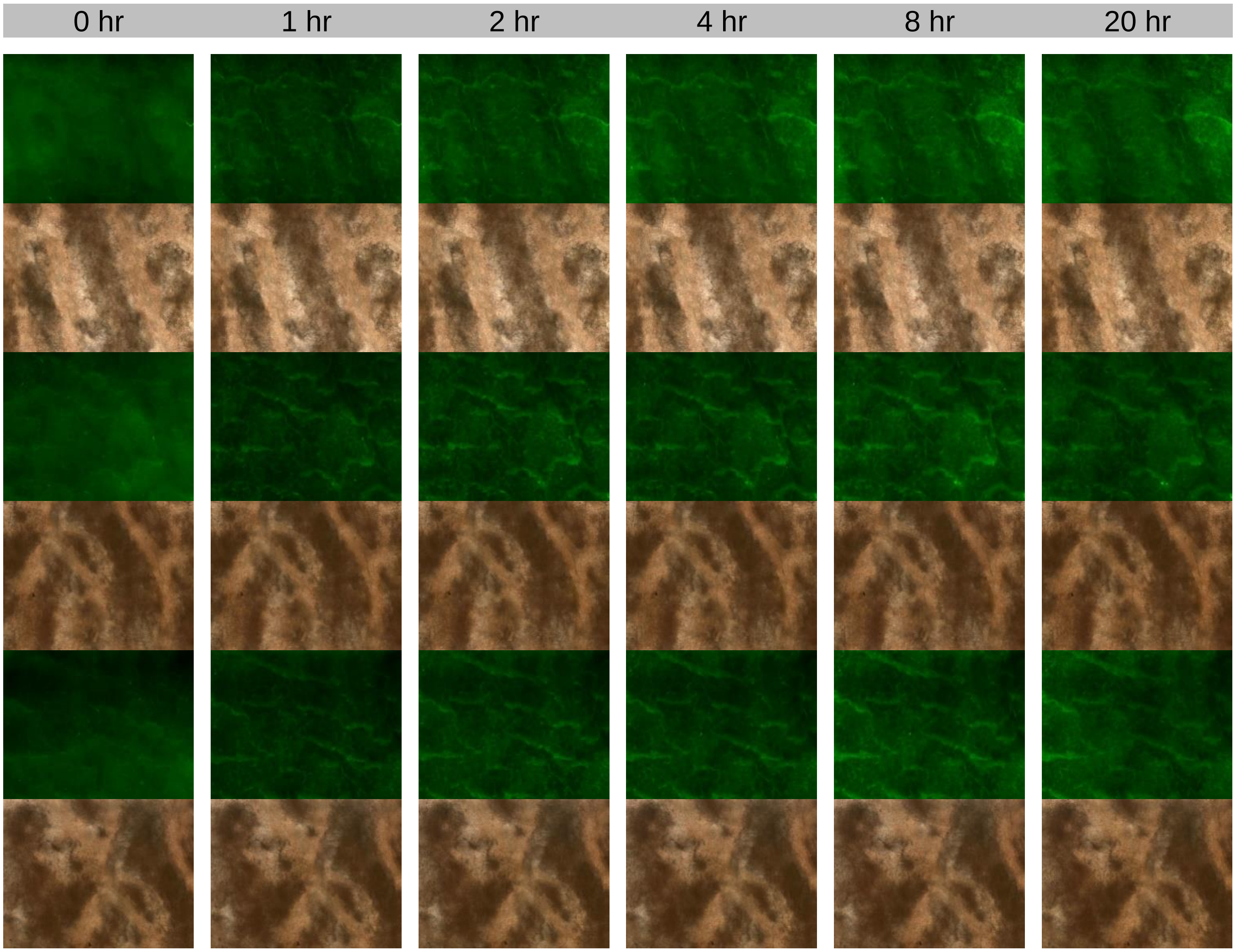

Supplementary Figure 45. Time-based imaging of carrot with no supplementation.

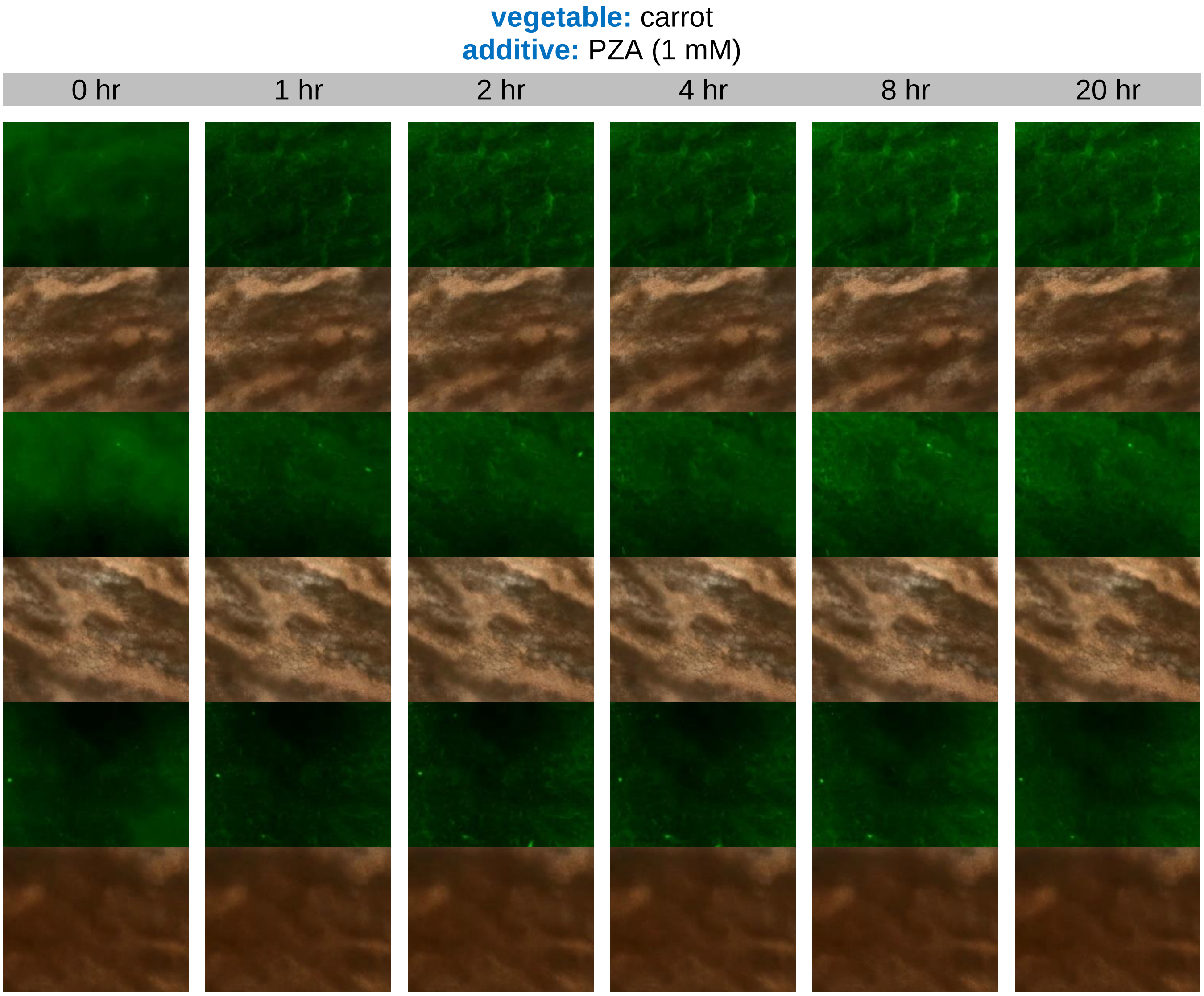

Supplementary Figure 46. Time-based imaging of carrot with 1 mM PZA supplementation.

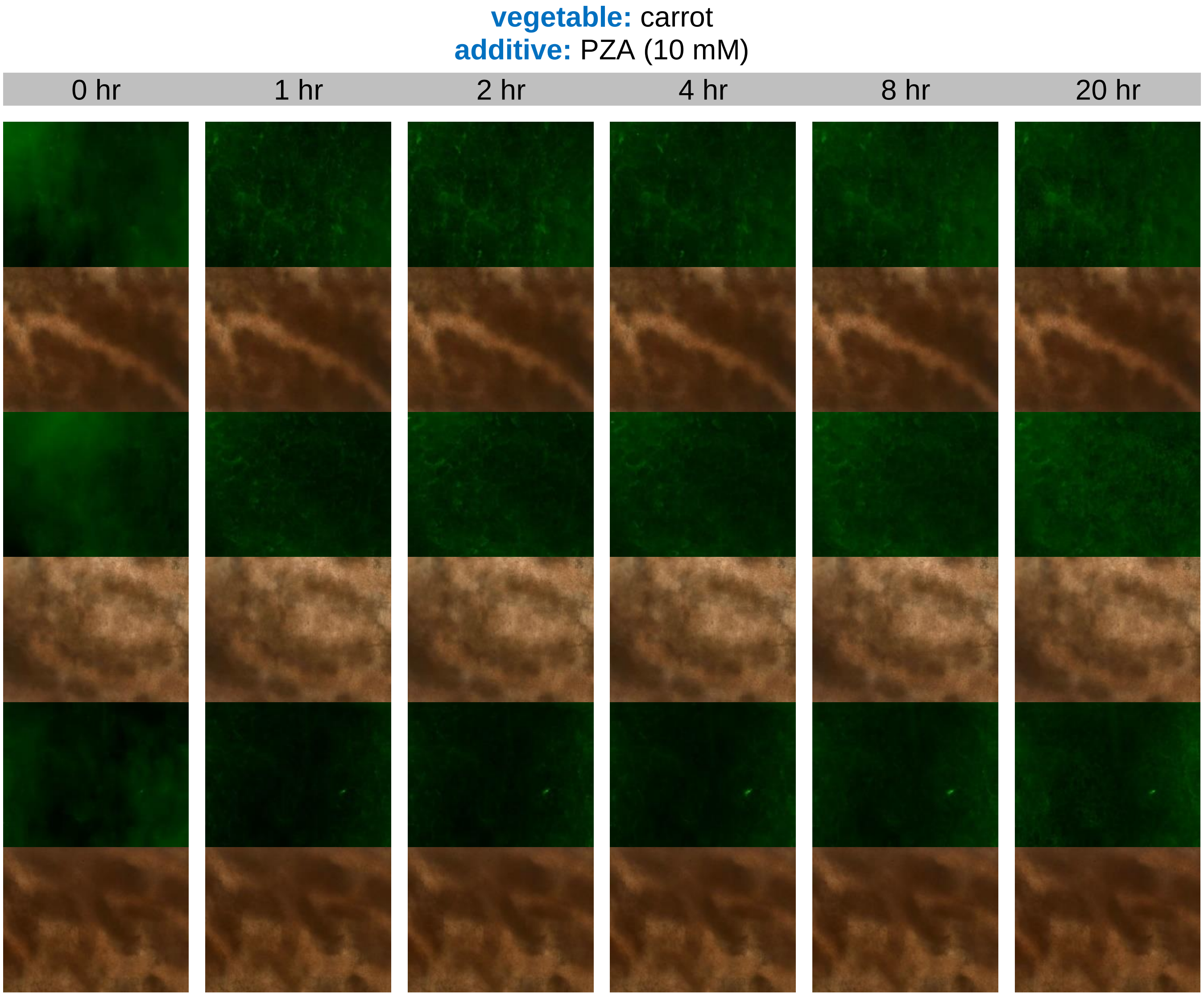

Supplementary Figure 47. Time-based imaging of carrot with 10 mM PZA supplementation.

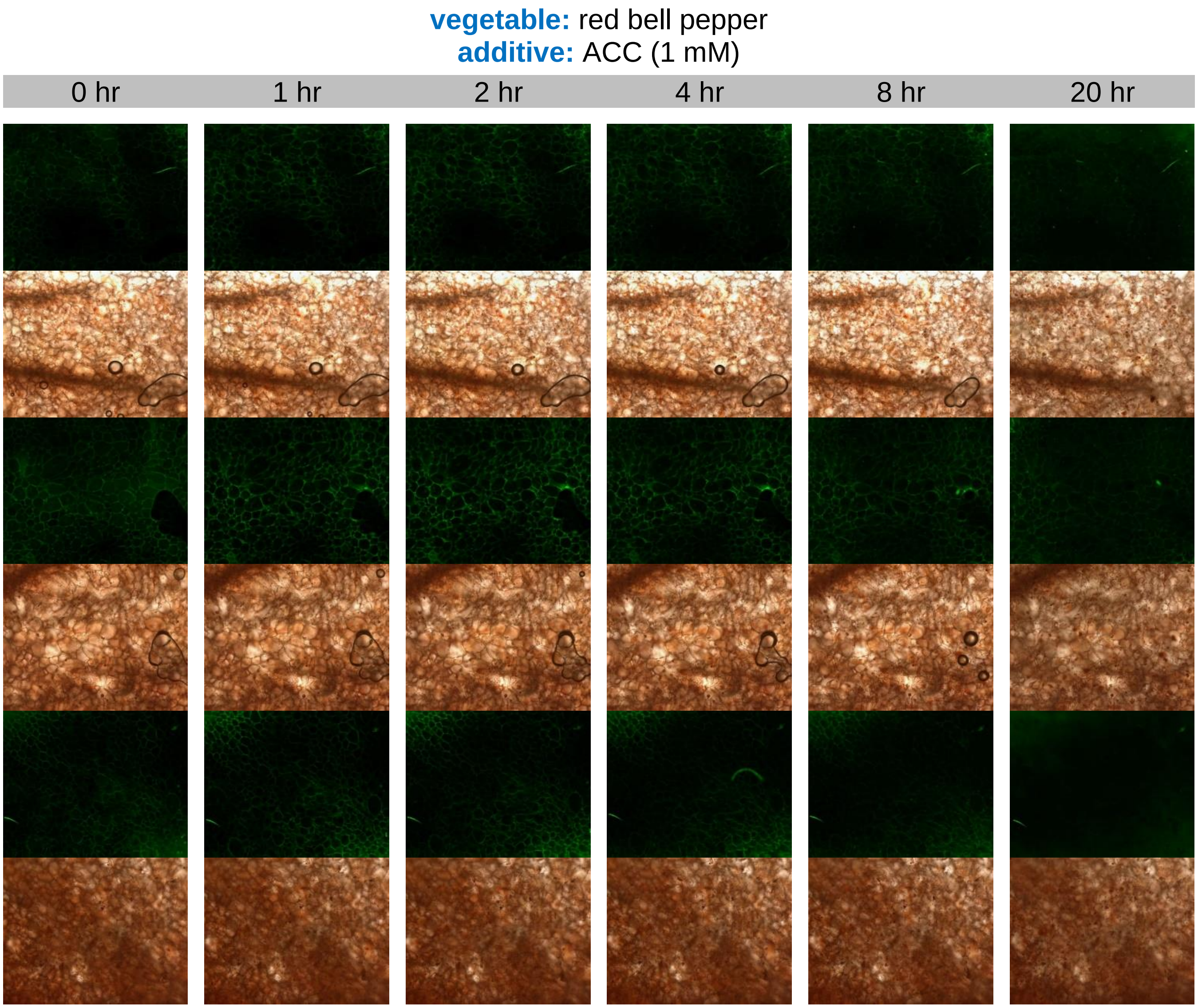

Supplementary Figure 48. Time-based imaging of red bell pepper with 1 mM ACC supplementation.

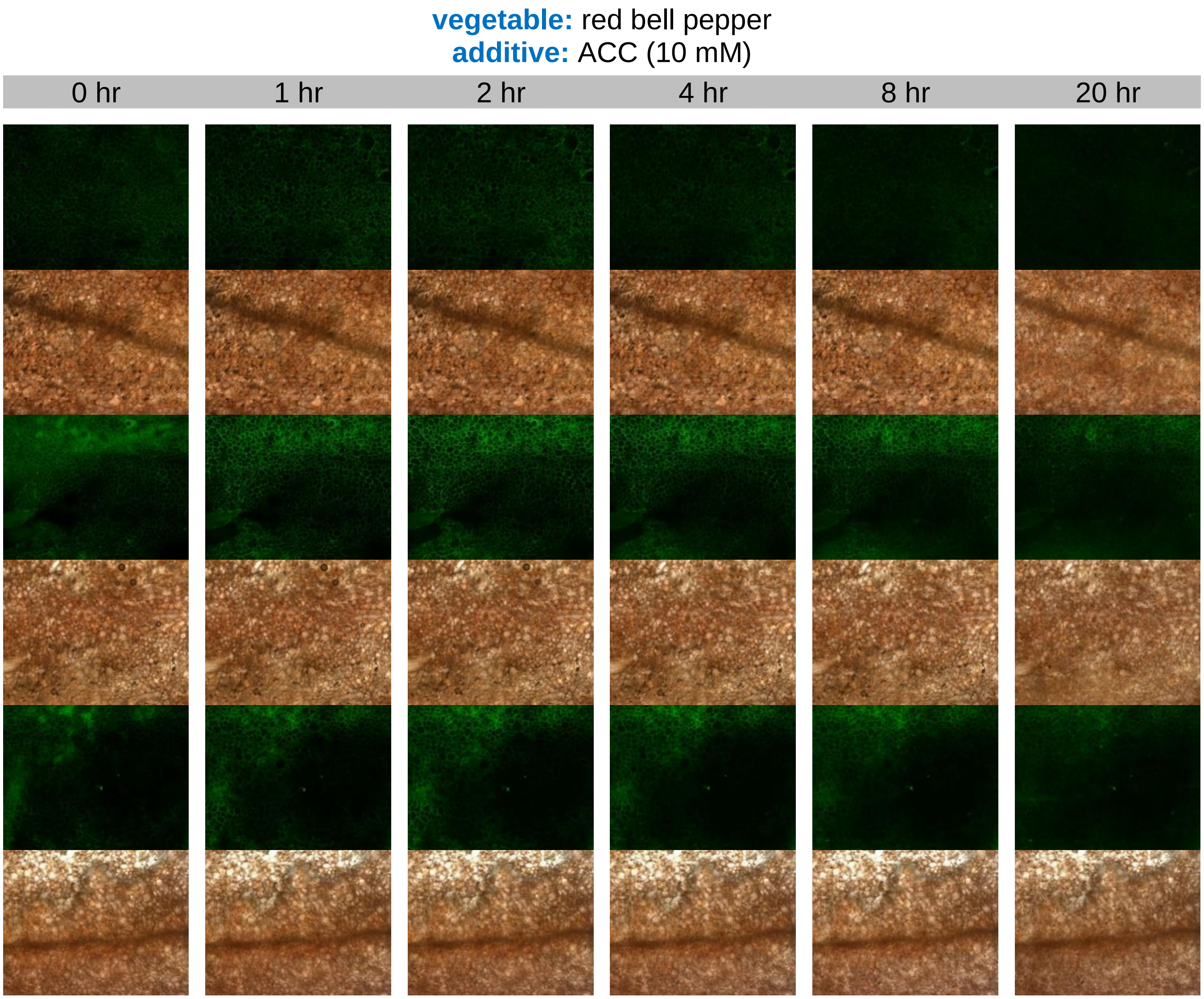

Supplementary Figure 49. Time-based imaging of red bell pepper with 10 mM ACC supplementation.

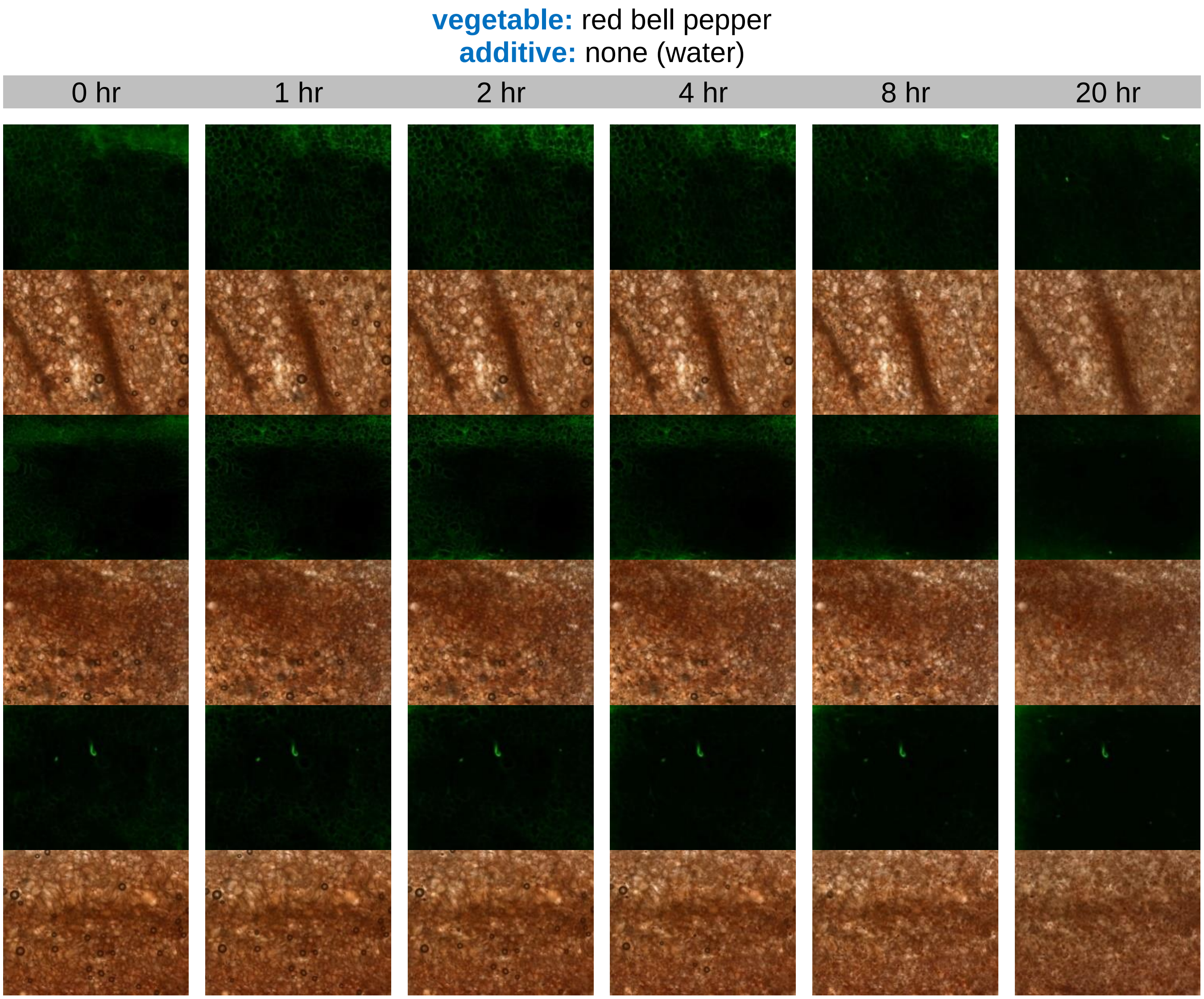

Supplementary Figure 50. Time-based imaging of red bell pepper with no supplementation.

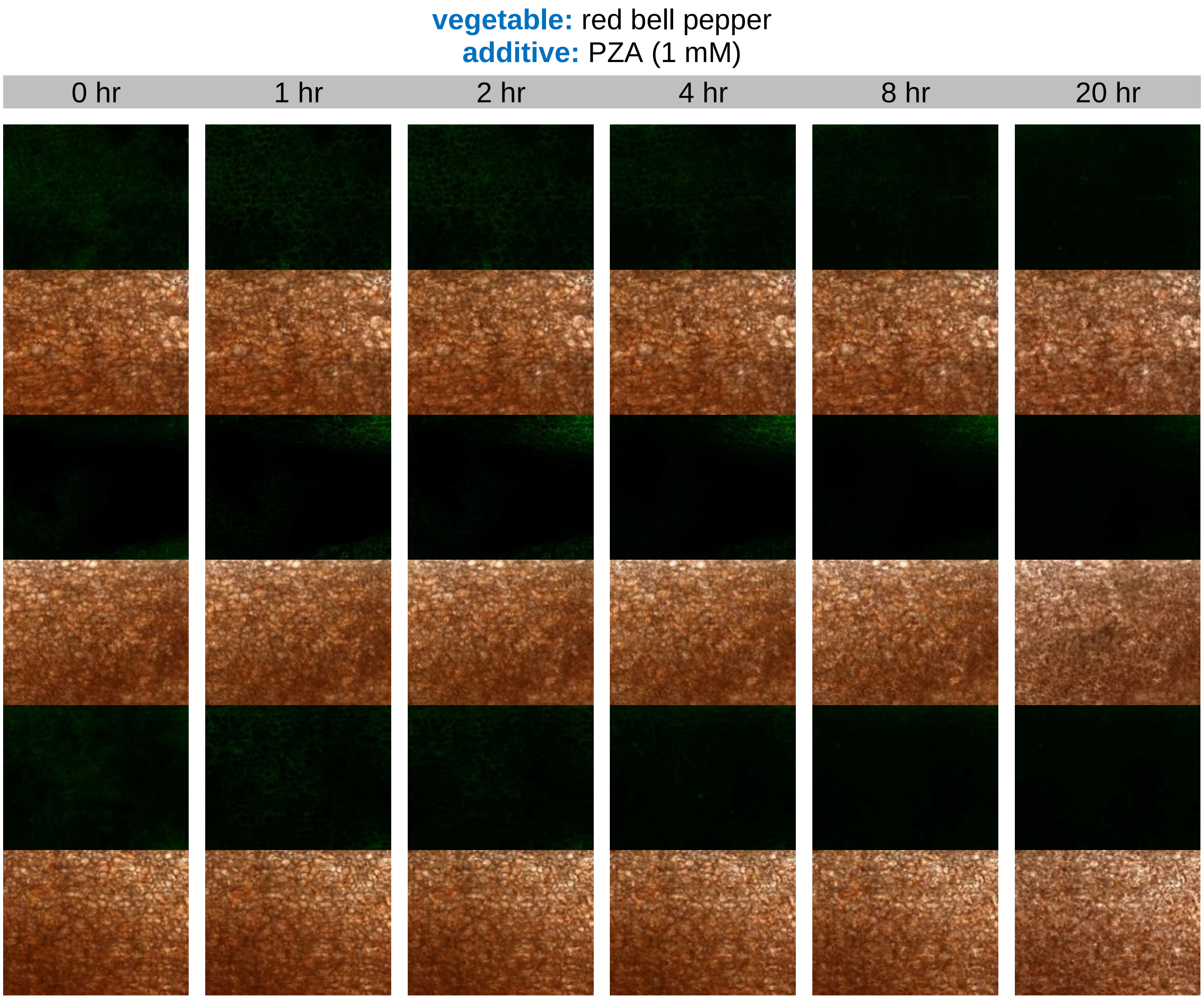

Supplementary Figure 51. Time-based imaging of red bell pepper with 1 mM PZA supplementation.

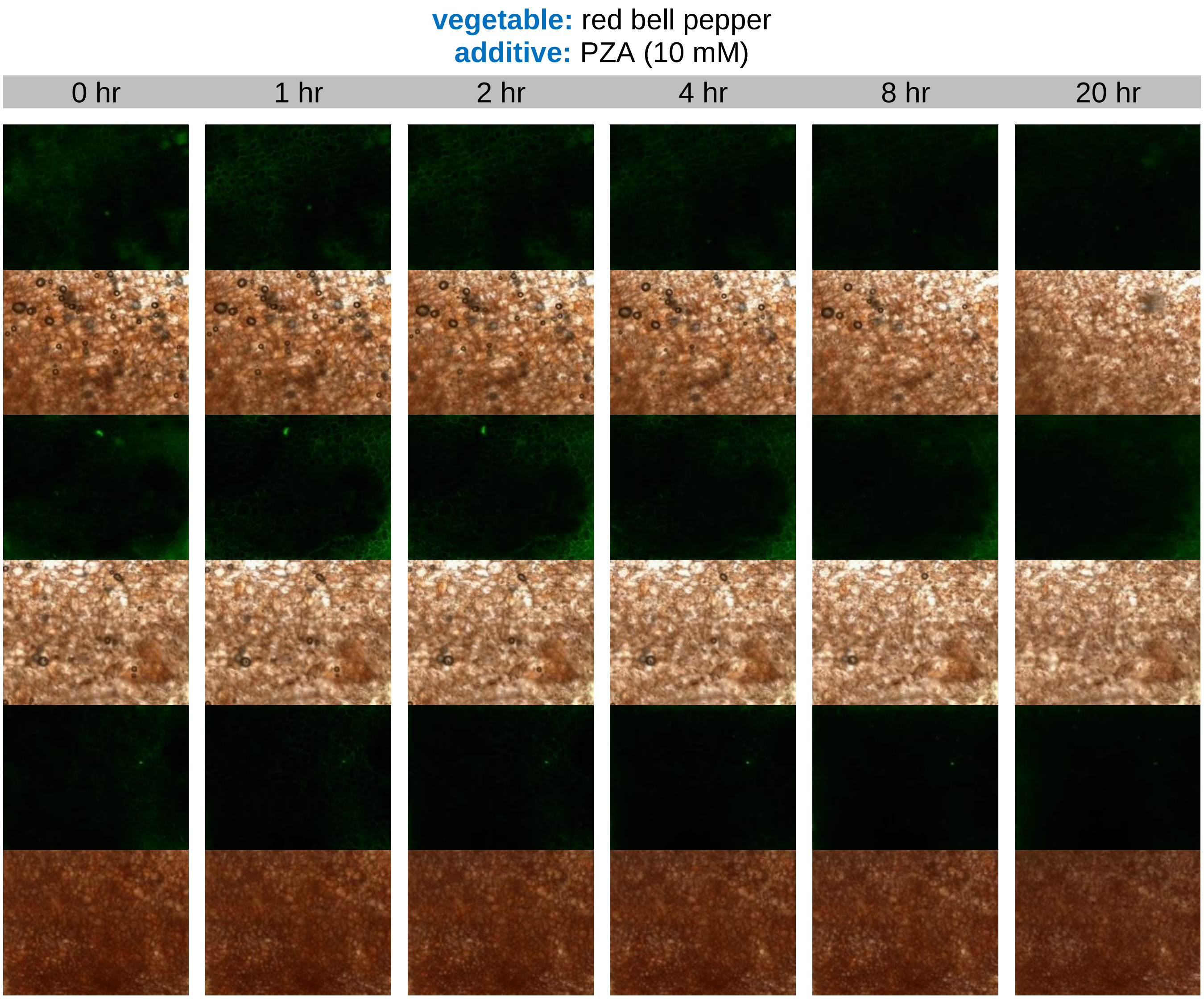

Supplementary Figure 52. Time-based imaging of red bell pepper with 10 mM PZA supplementation.

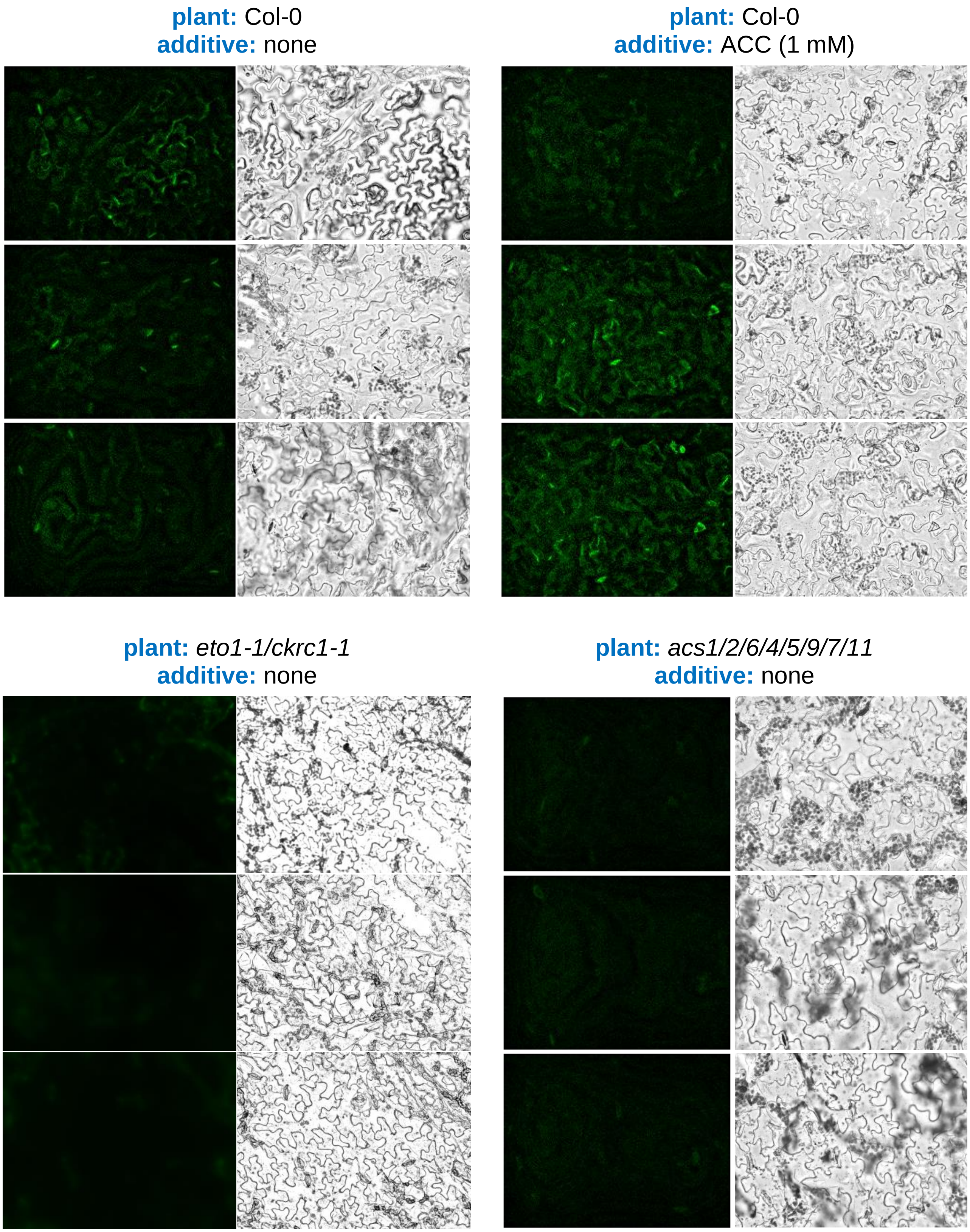

Supplementary Figure 53. Imaging (40× magnification) of epidermal peels treated without **AEP** probe for various strains of *A.thaliana* tissues under various conditions.

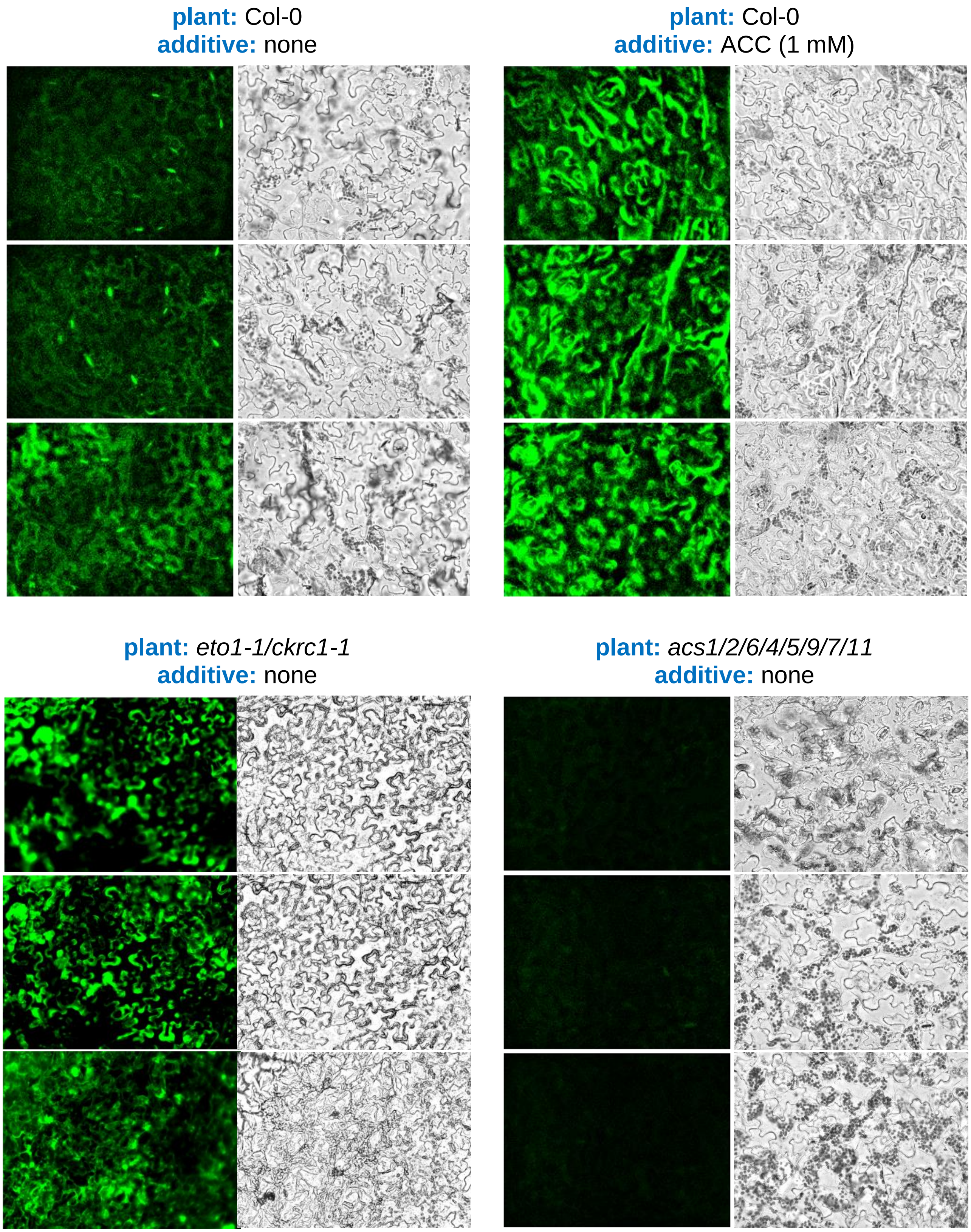

Supplementary Figure 54. Imaging (40× magnification) of epidermal peels treated with **AEP** probe for various strains of *A.thaliana* tissues under various conditions.

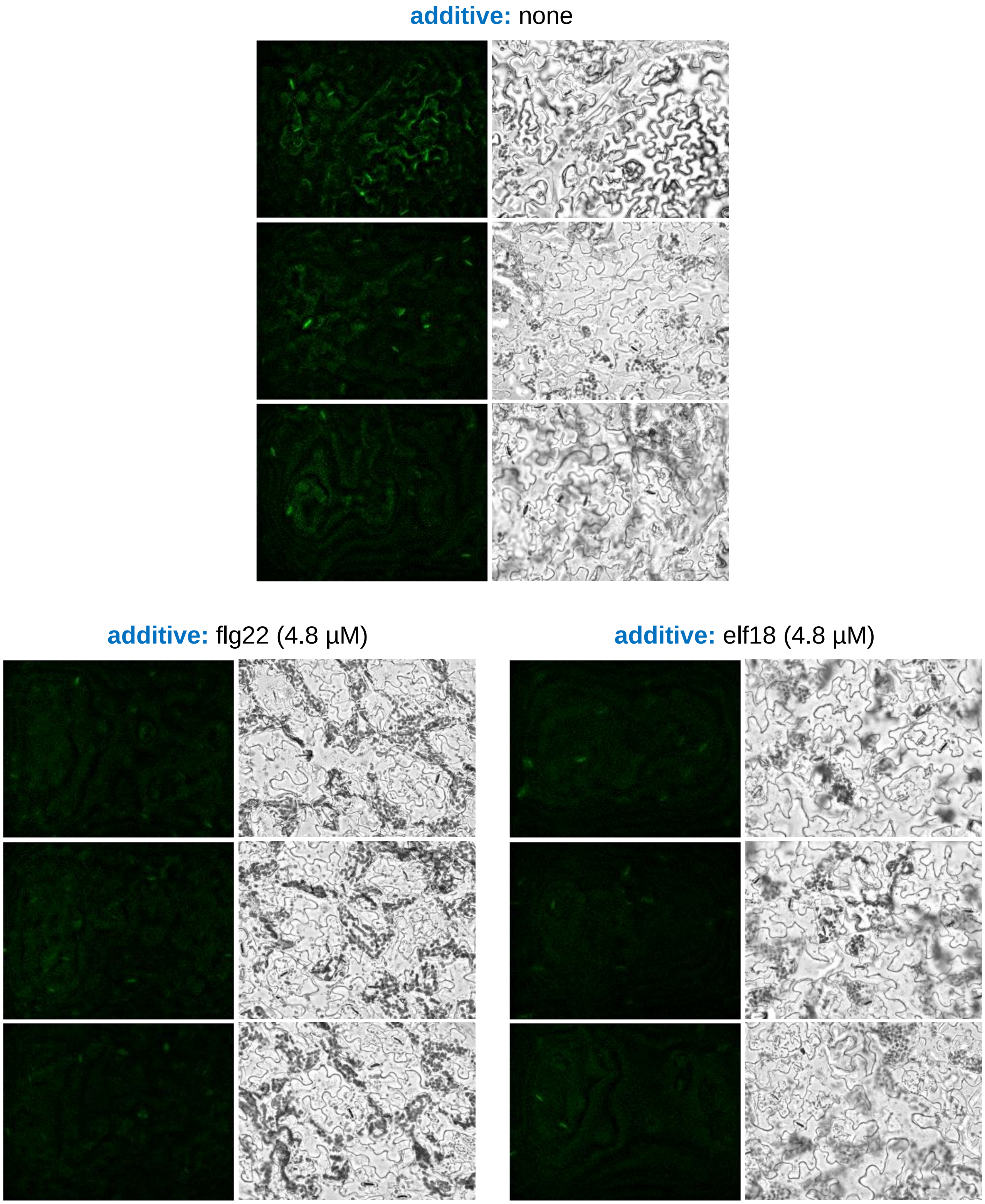

Supplementary Figure 55. Imaging (40× magnification) of epidermal peels treated without **AEP** probe for *A.thaliana* Col-0 tissues incubated in the presence and absence of PAMPs.

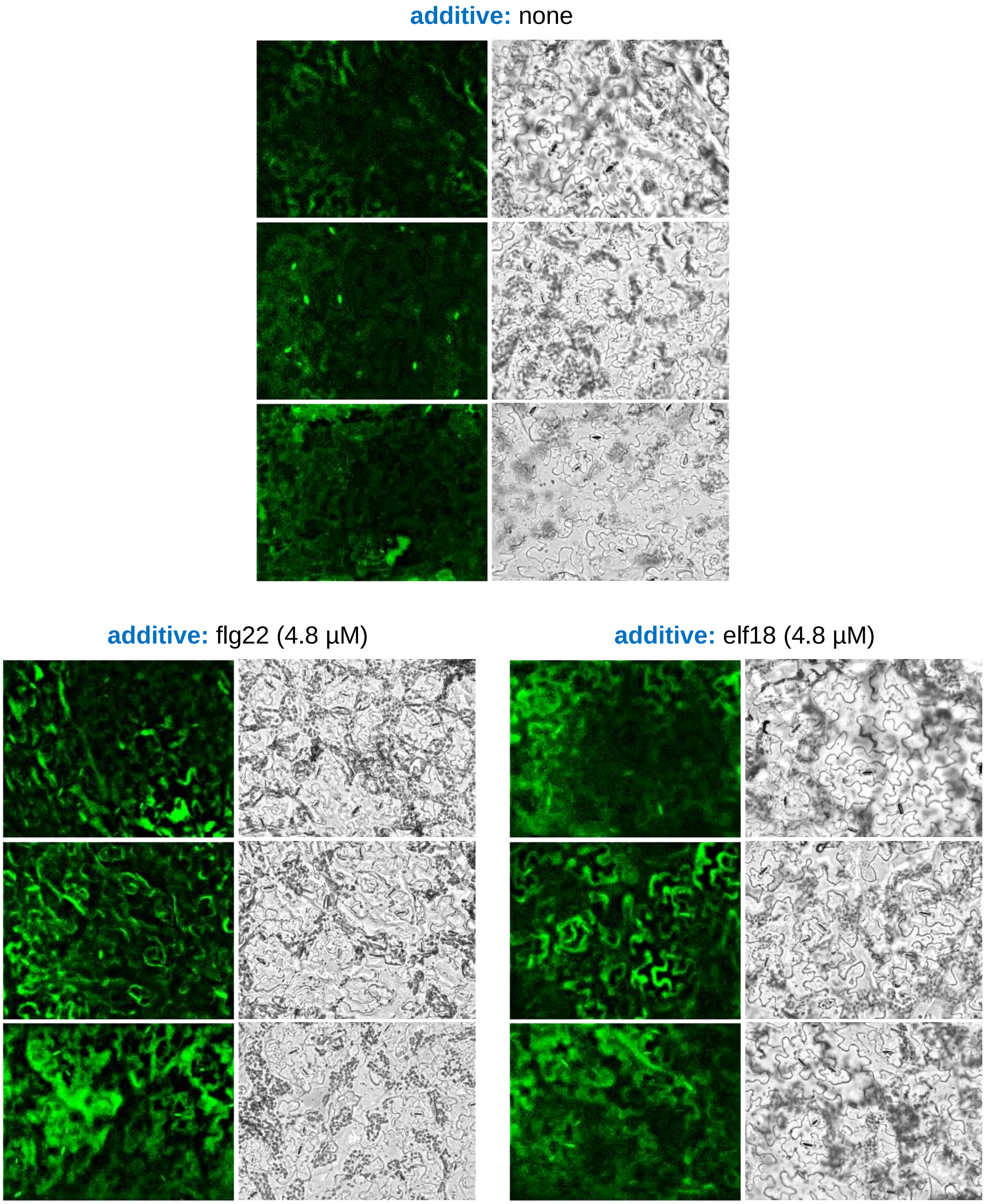

Supplementary Figure 56. Imaging (40× magnification) of epidermal peels treated with **AEP** probe for *A.thaliana* Col-0 tissues incubated in the presence and absence of PAMPs.

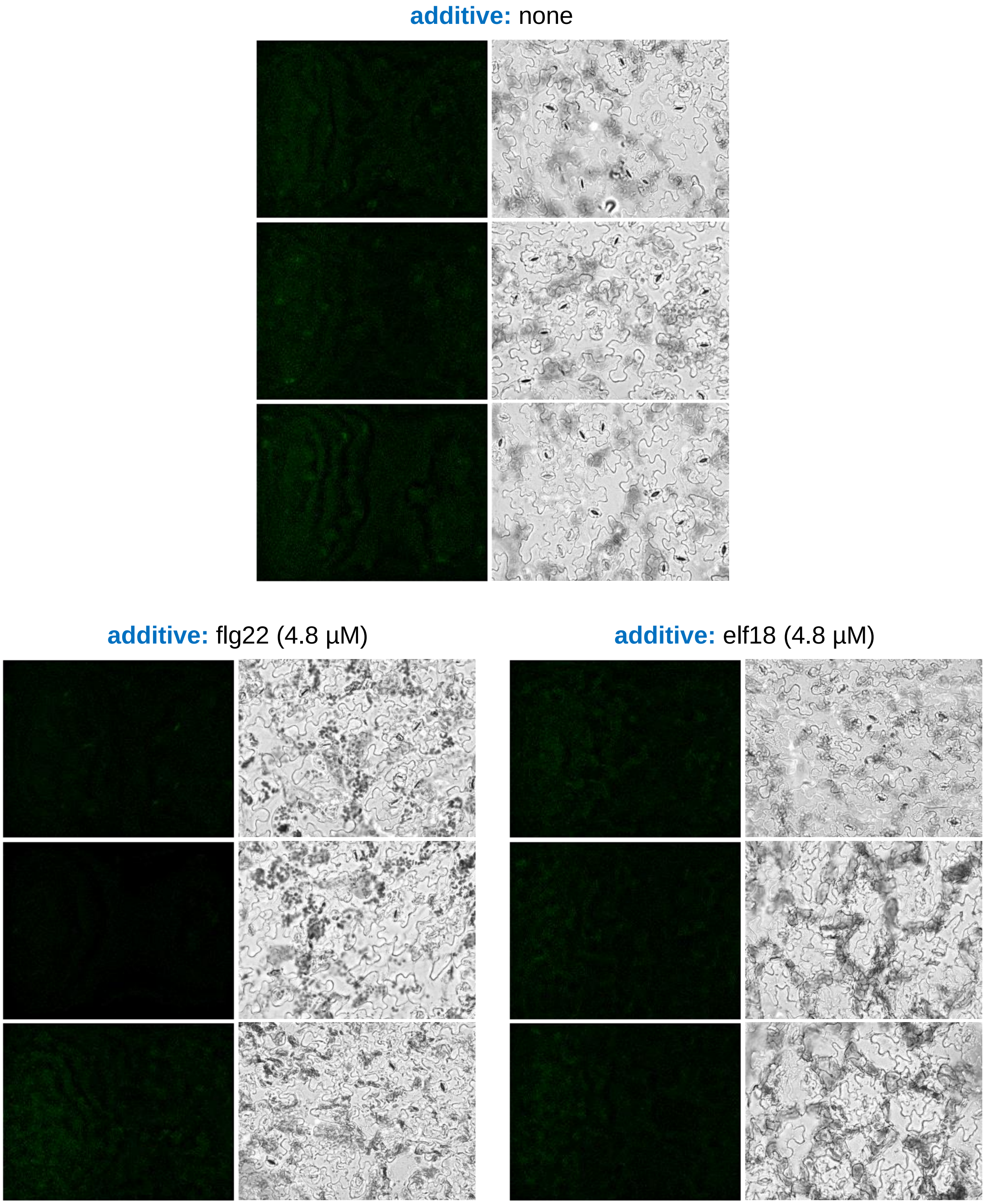

Supplementary Figure 57. Imaging (40× magnification) of epidermal peels treated without **AEP** probe for *A.thaliana fls2/efr/cerk1* tissues incubated in the presence and absence of PAMPs.

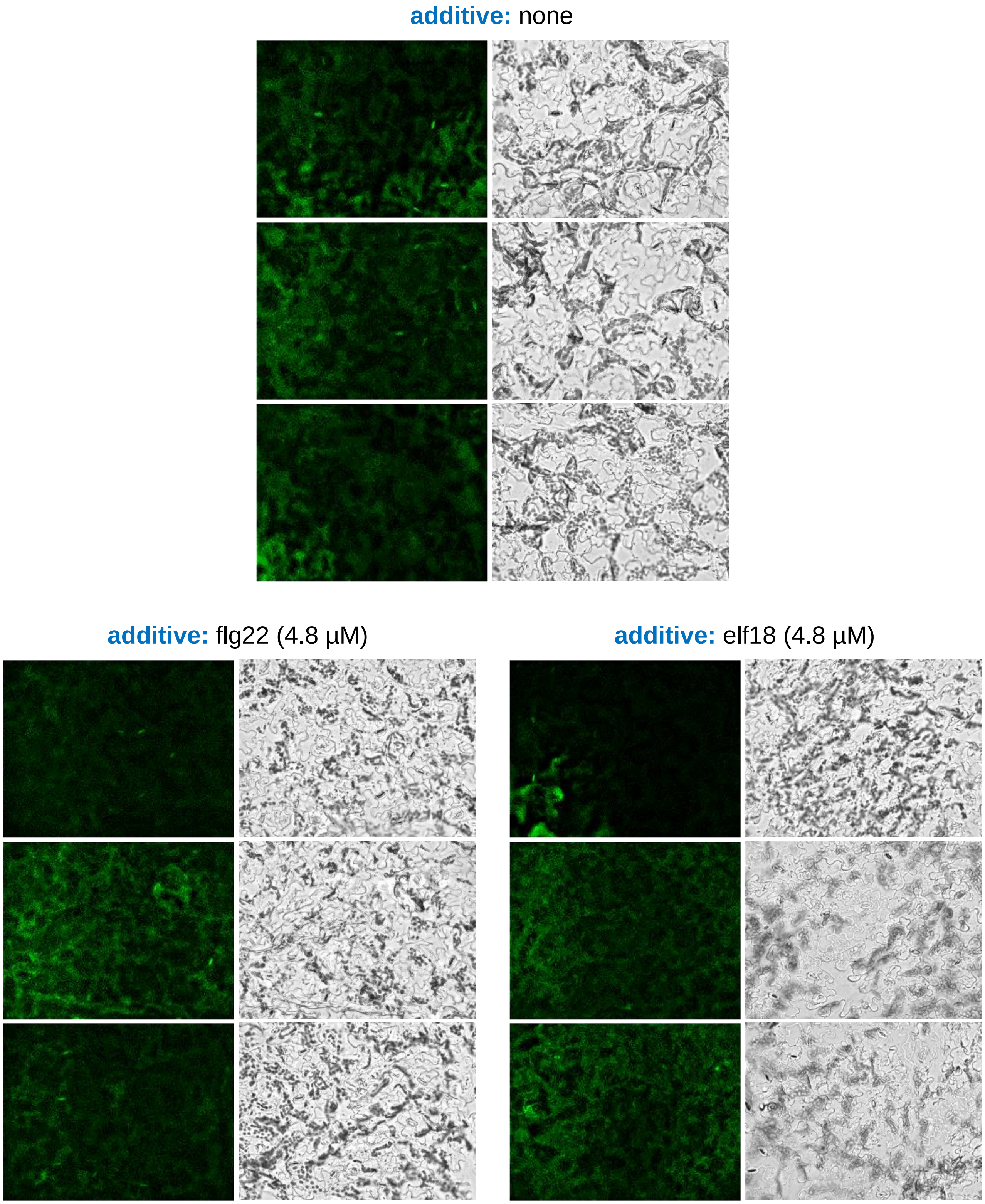

Supplementary Figure 58. Imaging (40× magnification) of epidermal peels treated with **AEP** probe for *A.thaliana fls2/efr/cerk1* tissues incubated in the presence and absence of PAMPs.

additive: none

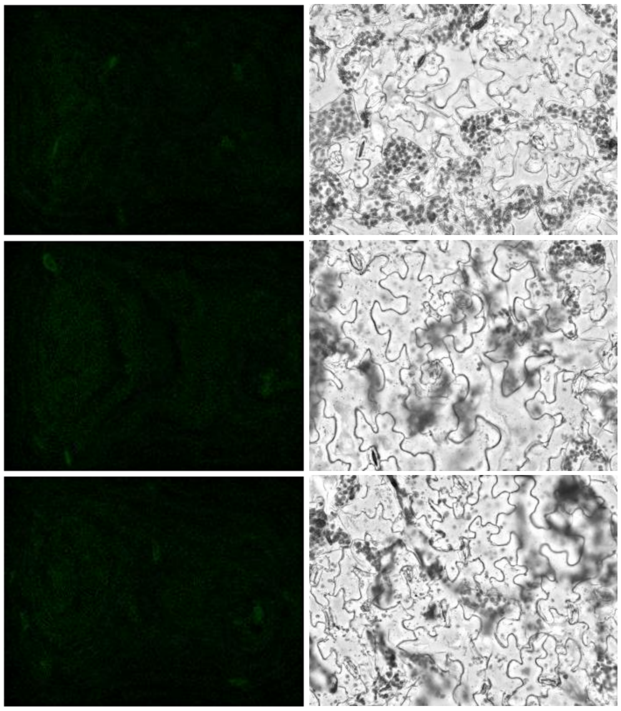

additive: flg22 (4.8 µM)

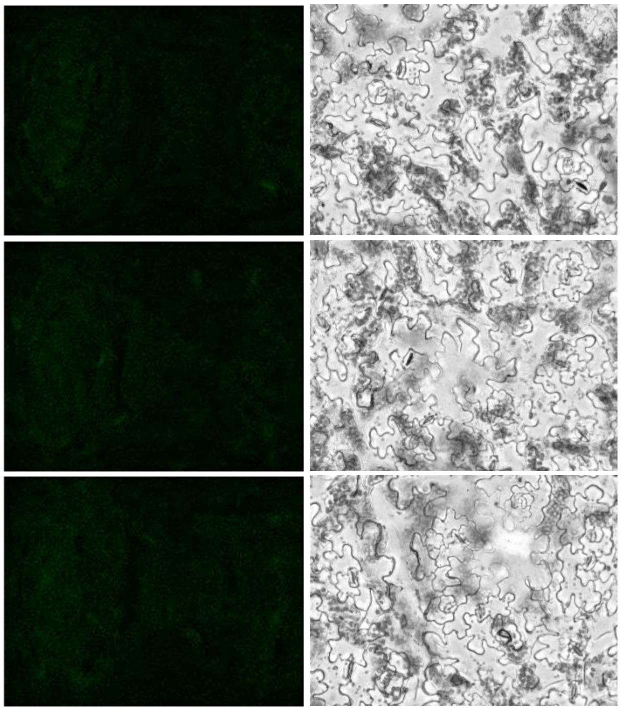

additive: elf18 (4.8 µM)

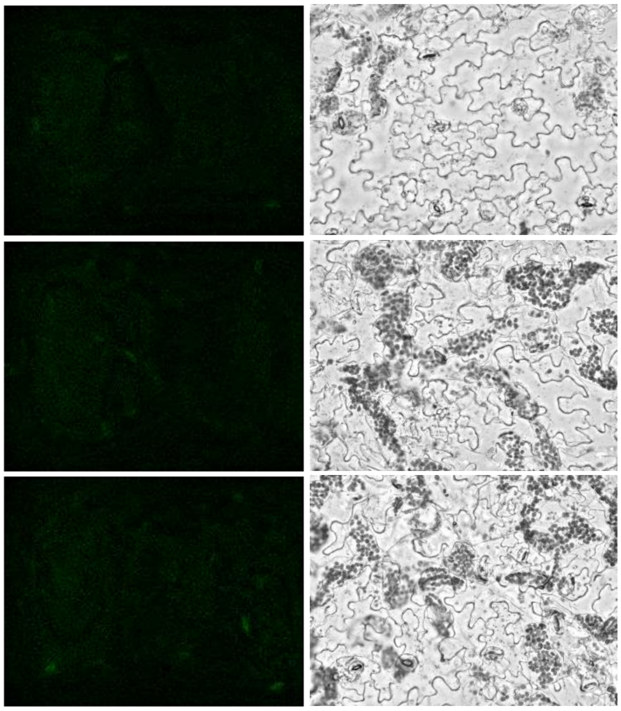

Supplementary Figure 59. Imaging (40× magnification) of epidermal peels treated without **AEP** probe for *A.thaliana acs1/2/6/4/5/9/7/11* tissues incubated in the presence and absence of PAMPs.

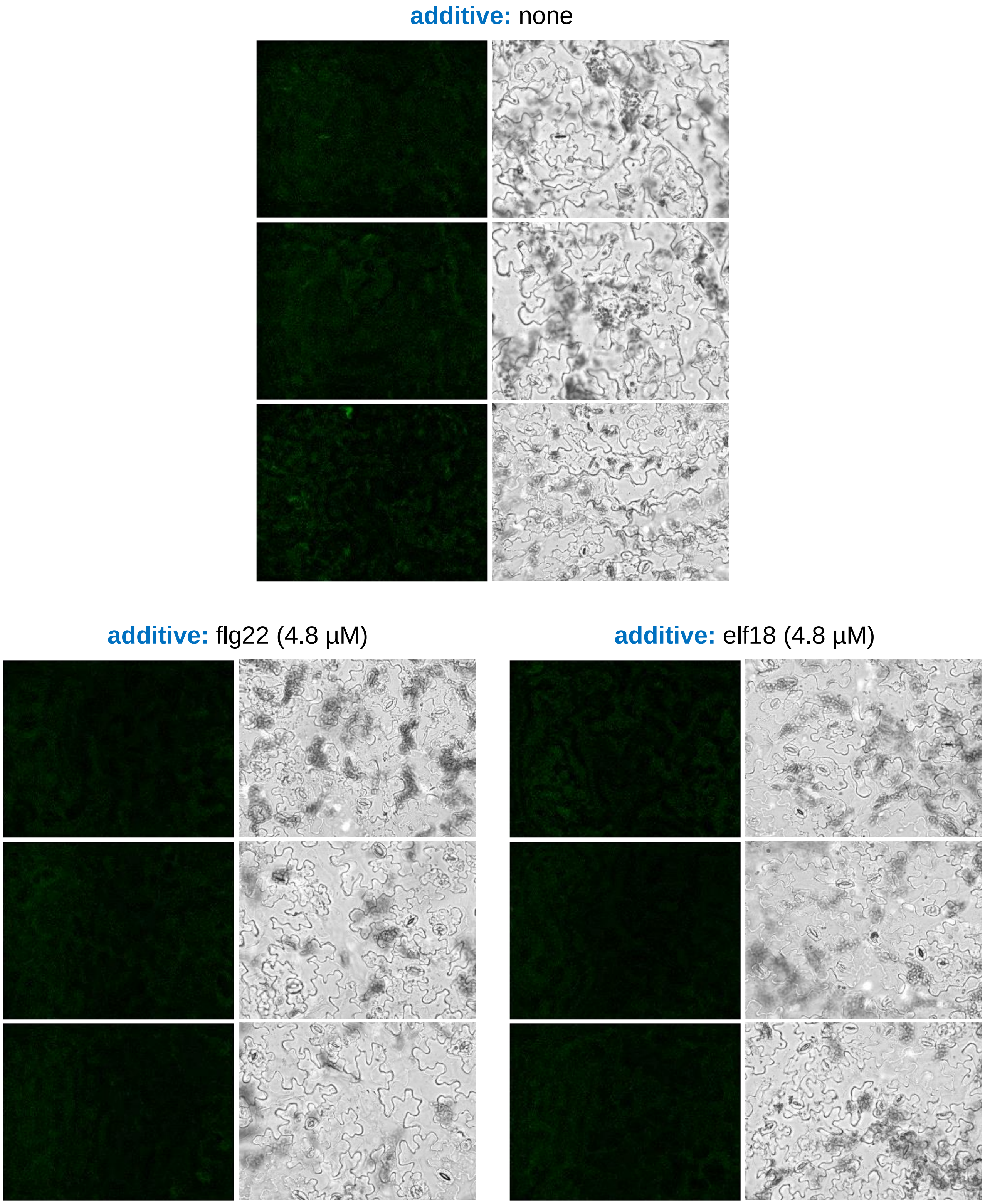

Supplementary Figure 60. Imaging (40× magnification) of epidermal peels treated with **AEP** probe for *A.thaliana acs1/2/6/4/5/9/7/11* tissues incubated in the presence and absence of PAMPs.

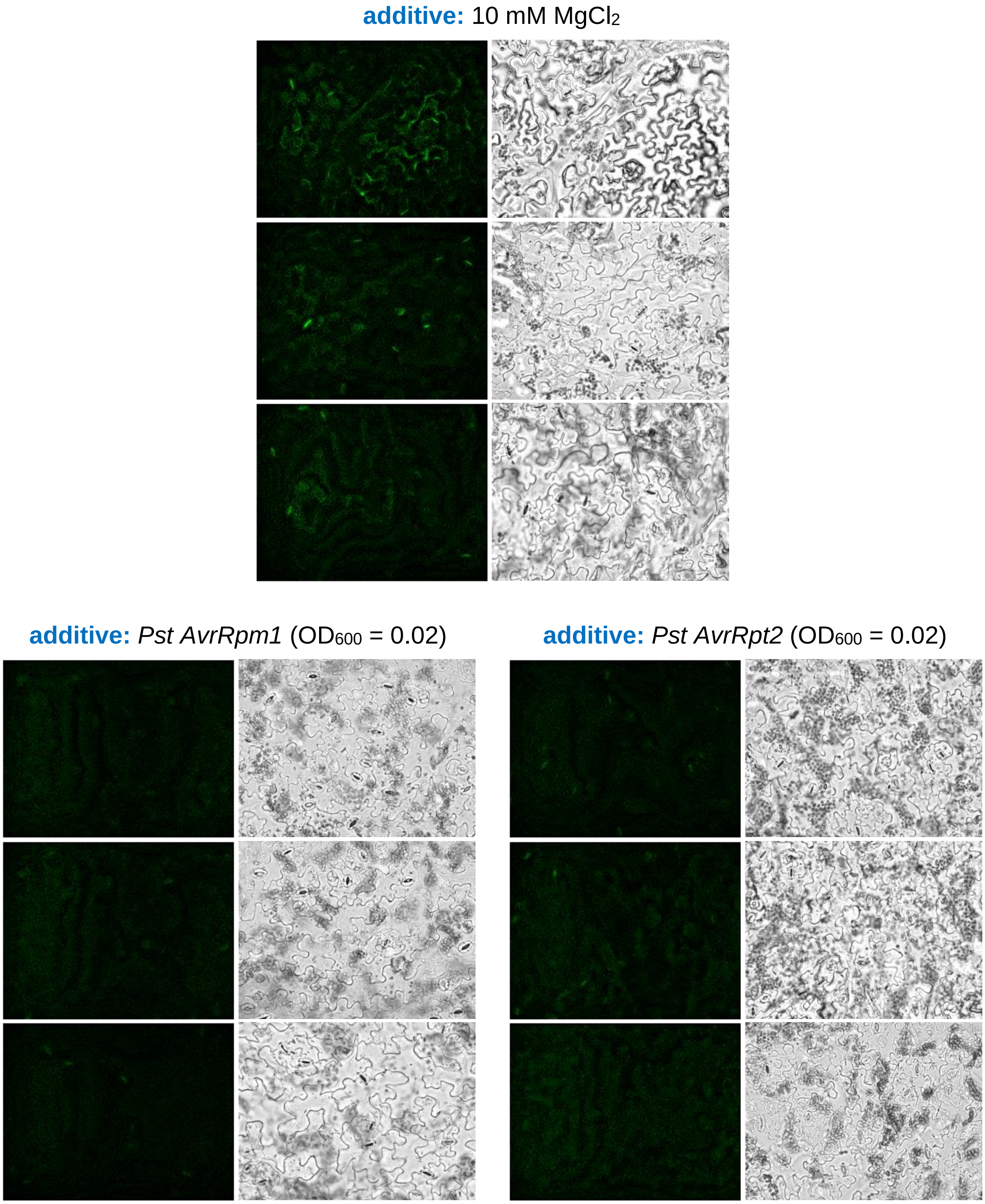

Supplementary Figure 61. Imaging (40× magnification) of epidermal peels treated without **AEP** probe for *A.thaliana* Col-0 tissues incubated in the presence and absence of Avr-producing *Pseudomonas* bacteria.

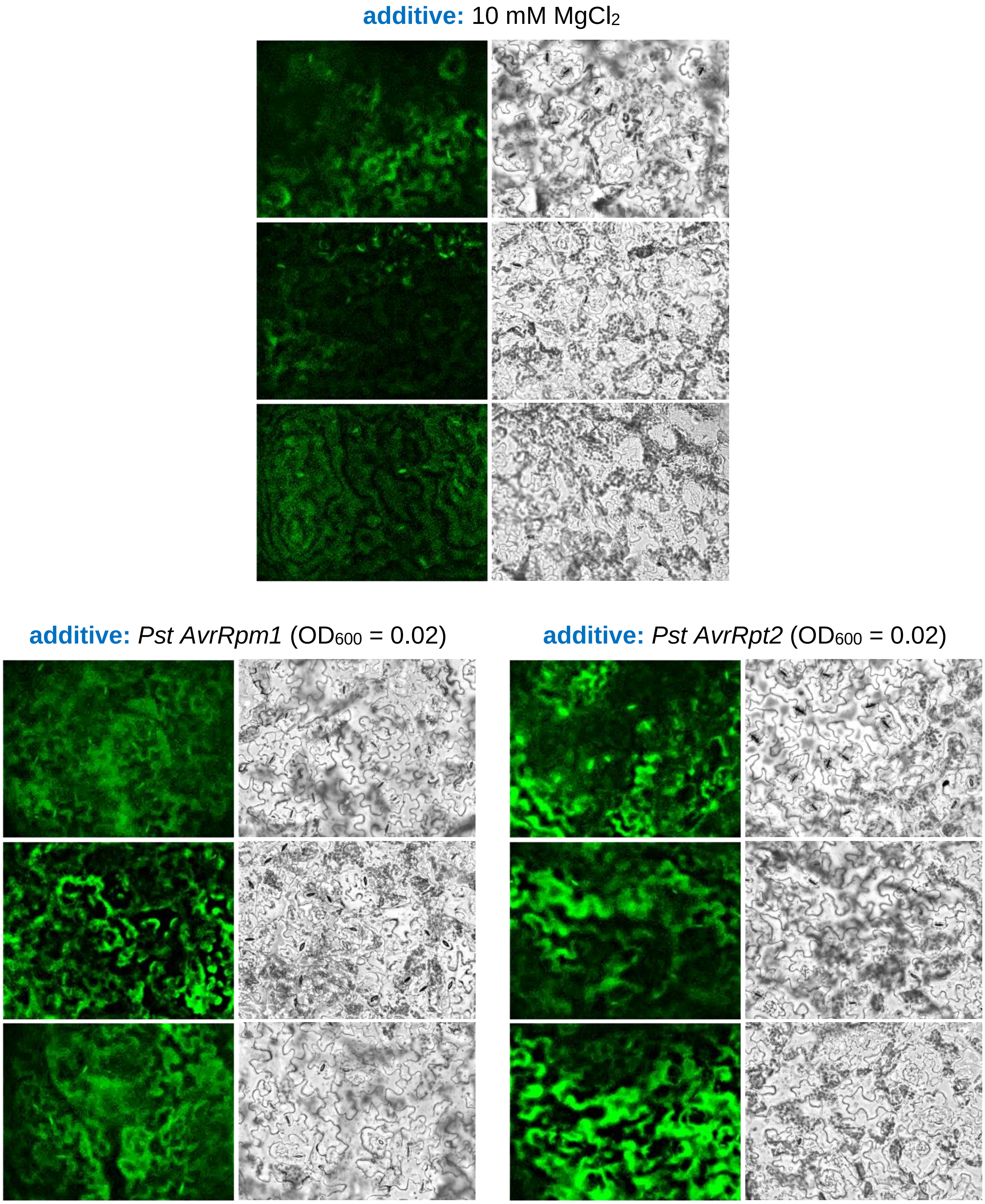

Supplementary Figure 62. Imaging (40× magnification) of epidermal peels treated with **AEP** probe for *A.thaliana* Col-0 tissues incubated in the presence and absence of Avr-producing *Pseudomonas* bacteria.

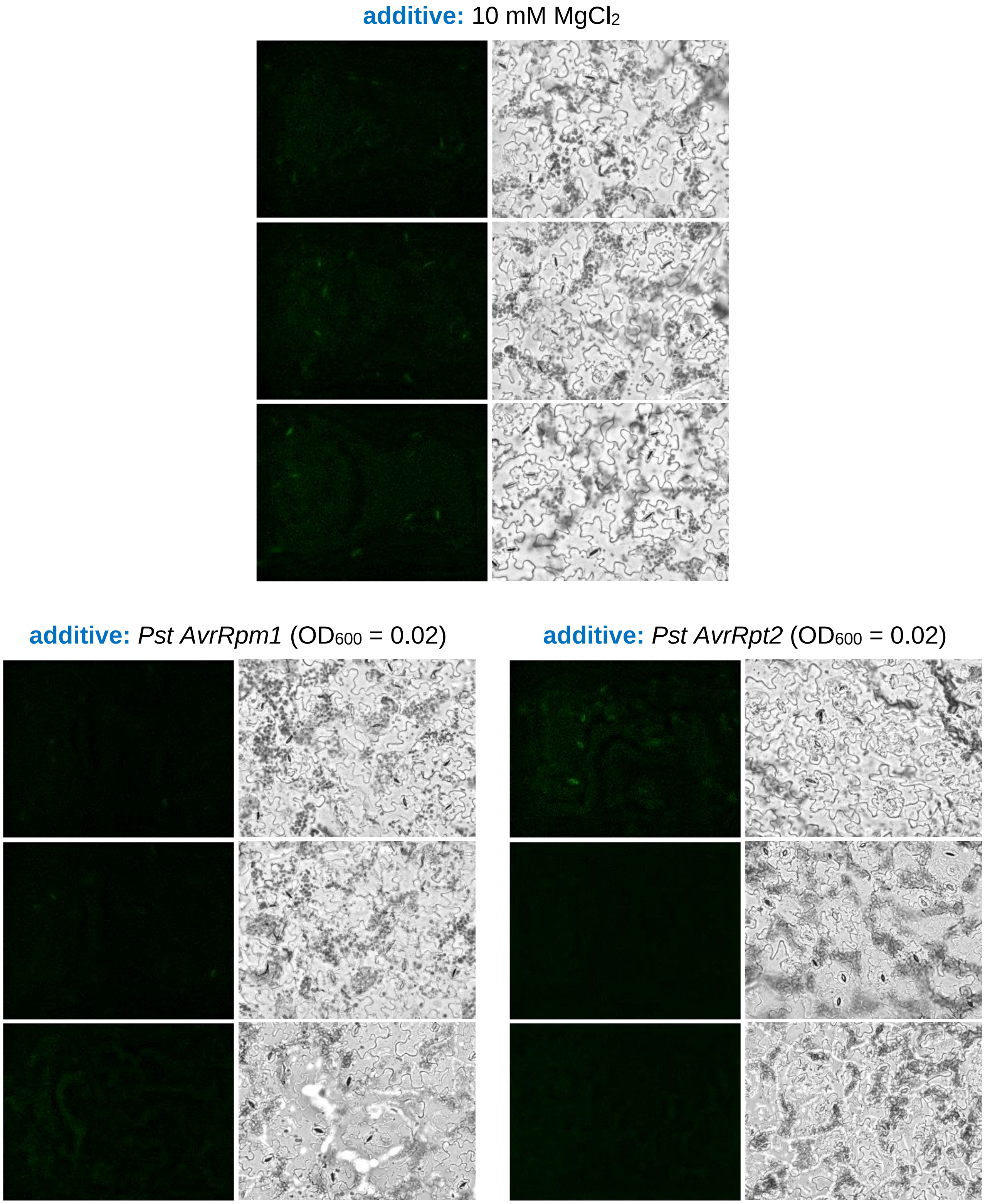

Supplementary Figure 63. Imaging (40× magnification) of epidermal peels treated without **AEP** probe for *A.thaliana rpm1rps2* tissues incubated in the presence and absence of Avr-producing *Pseudomonas* bacteria.

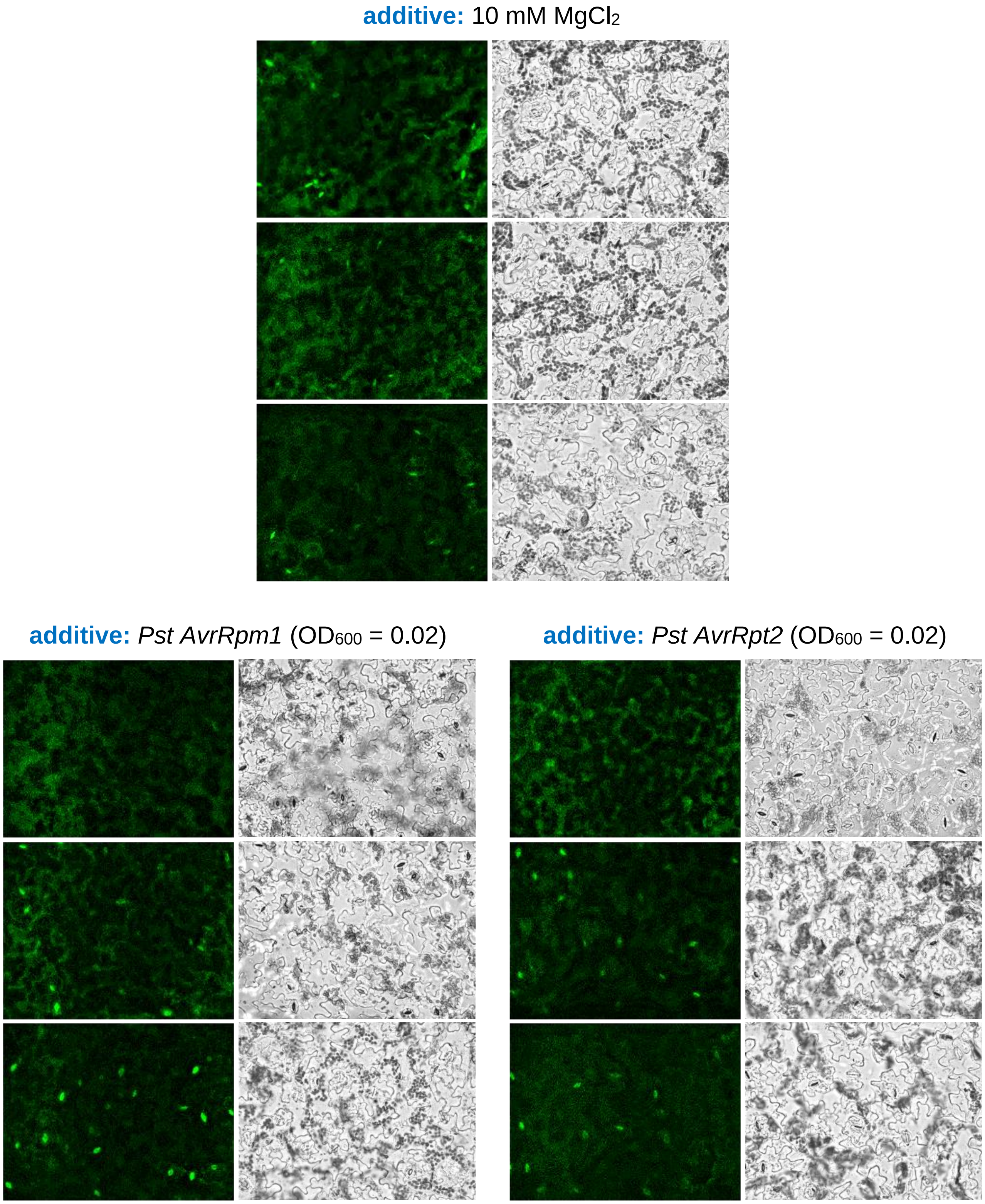

Supplementary Figure 64. Imaging (40× magnification) of epidermal peels treated with **AEP** probe for *A.thaliana rpm1rps2* tissues incubated in the presence and absence of Avr-producing *Pseudomonas* bacteria.

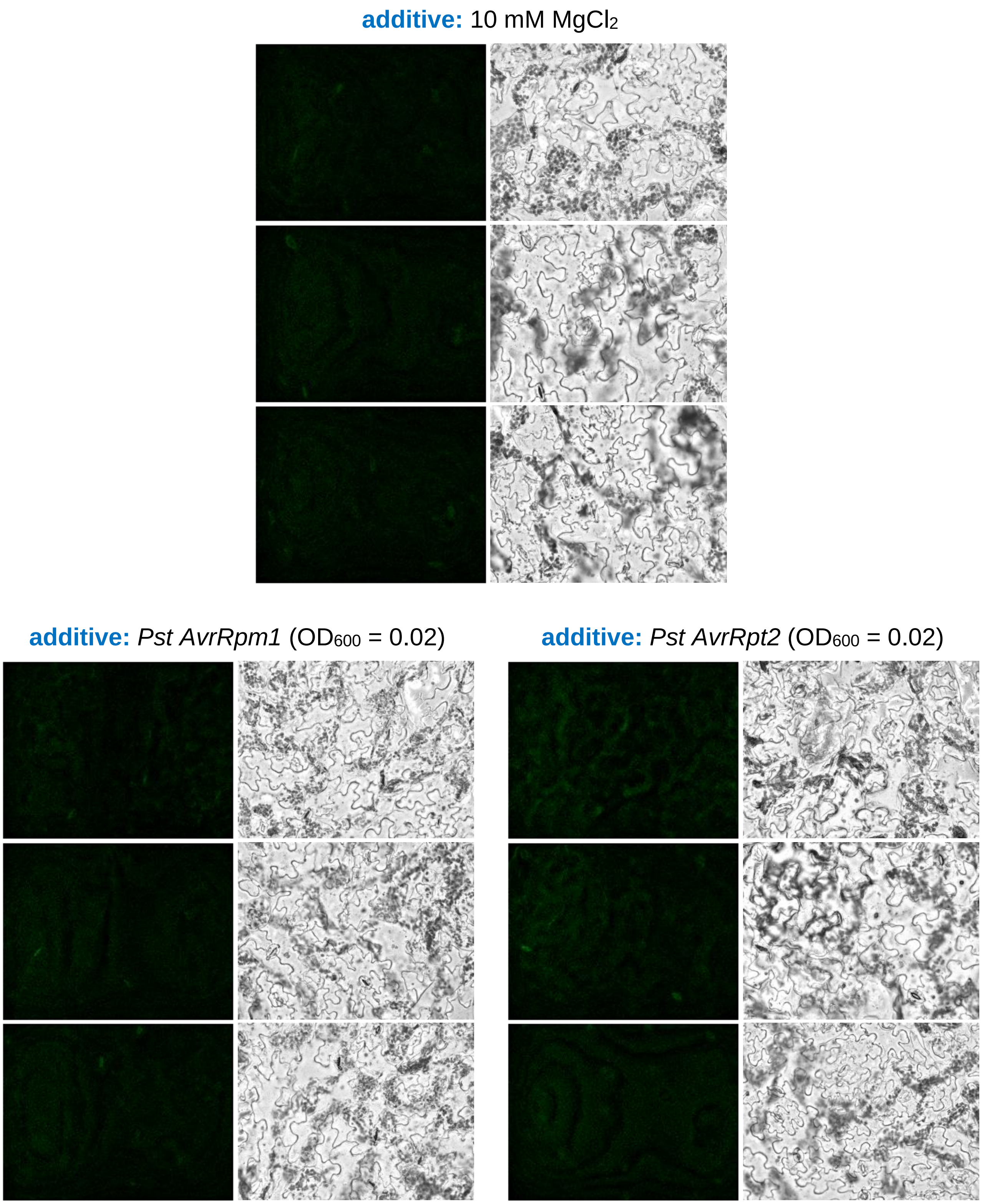

Supplementary Figure 65. Imaging (40× magnification) of epidermal peels treated without **AEP** probe for *A.thaliana acs1/2/6/4/5/9/7/11* tissues incubated in the presence and absence of Avr-producing *Pseudomonas*.

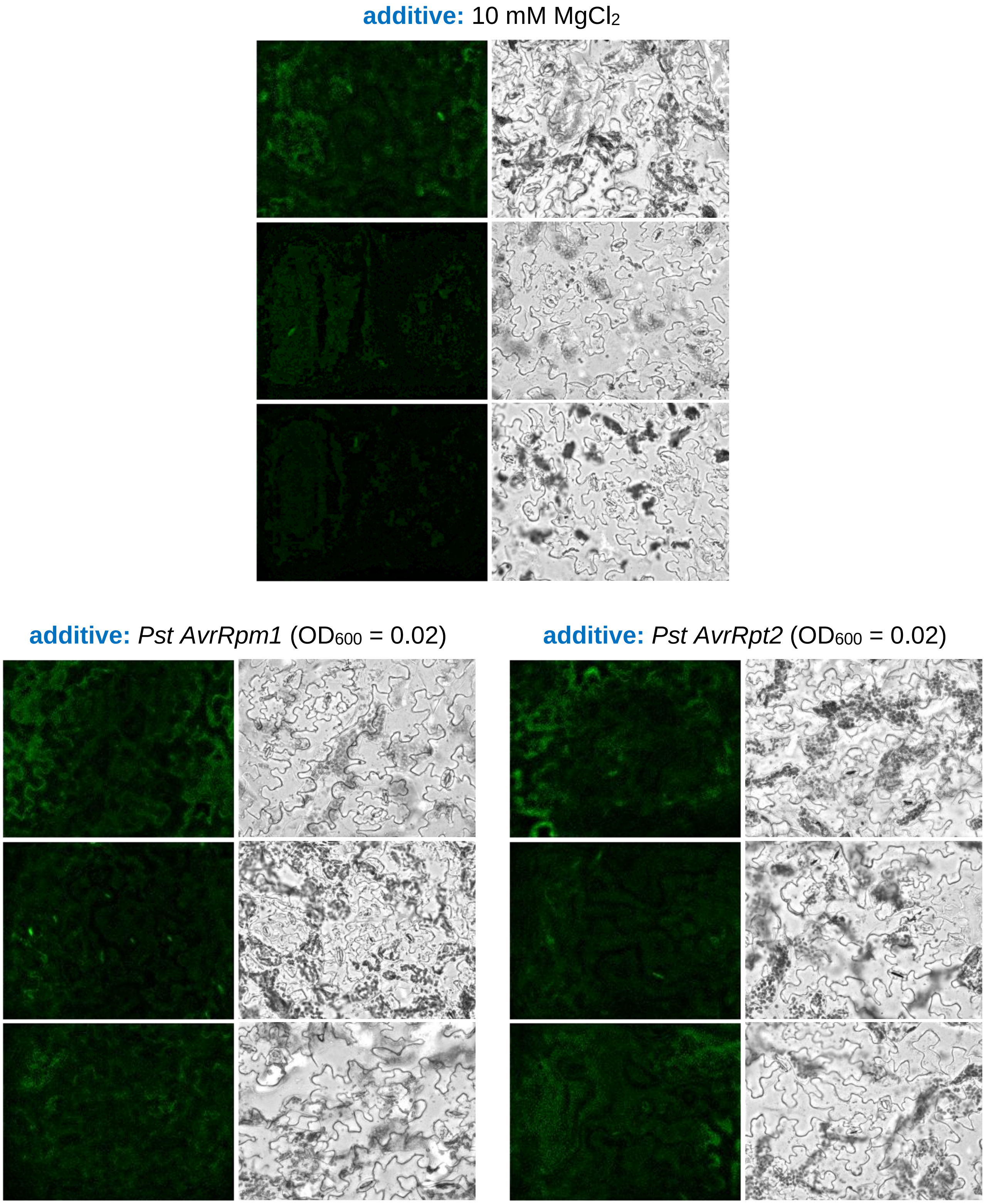

Supplementary Figure 66. Imaging (40× magnification) of epidermal peels treated with **AEP** probe for *A.thaliana acs1/2/6/4/5/9/7/11* tissues incubated in the presence and absence of Avr-producing *Pseudomonas*.

Supplementary Methods

Preparation of **II**

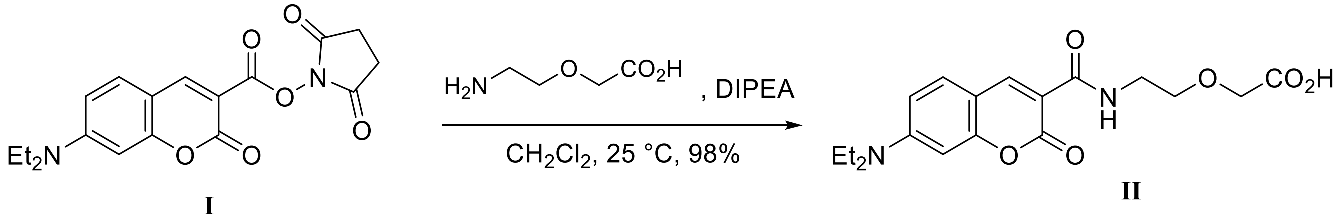

I **II**

To a solution of *N*-succinimidyl 7-(diethylamino)coumarin-3-carboxylate **I** (100 mg, 0.279 mmol) and 5-amino-3-oxapentanoic acid (36.6 mg, 0.307 mmol) in CH_2Cl_2 (3 mL) was added *N*,*N*-diisopropylethylamine (110 mg, 0.851 mmol) at 25 °C. After stirring for 16 h, the reaction was stopped by addition of 1 M aq. HCl. The product was then extracted with CH_2Cl_2 (×3), and the combined organic extracts were washed with brine, dried (Na_2SO_4), and concentrated *in vacuo*. The residue was purified by silica-gel flash column chromatography ($CHCl_3$/MeOH = 9/1 → 8/2 → $CHCl_3$/MeOH/AcOH = 79/20/1) to give the desired product (100 mg, 98.9%) as a yellow solid.

^{1}H-NMR (500 MHz, $CDCl_3$, δ) 1.22 (t, 6H, *J* = 6.9 Hz), 3.41 (q, 4H, *J* = 6.9 Hz), 3.64–3.72 (m, 4H), 4.10 (s, 2H), 6.44 (s, 1H, *J* = 2.3 Hz), 6.61 (d, 1H, *J* = 8.8 Hz), 7.41 (d, 1H, *J* = 8.8 Hz), 8.68 (s, 1H), 9.13 (s, 1H); ^{13}C-NMR (125 MHz, $CDCl_3$, δ) 12.4 (2C), 39.6, 45.1 (2C), 69.4, 70.7, 96.5, 108.4, 109.6, 110.0, 131.3, 148.5, 152.6, 157.6, 162.5, 164.1, 173.8;

HRMS (ESI) *m*/*z* 363.1555 (363.1551 calcd for $C_{18}H_{23}N_2O_6$, $[M+H]^+$).

Preparation of DEAC-Ru

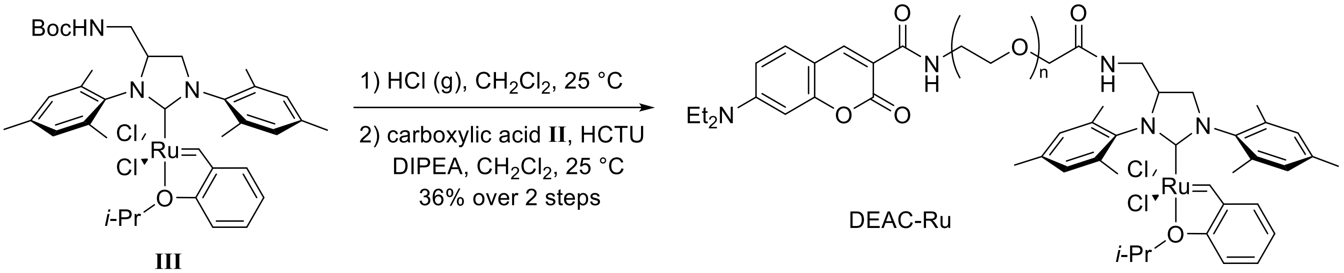

III

Prepared according to literature,[1-3] a solution of ruthenium complex **III** (80.1 mg, 0.106 mmol) in CH_2Cl_2 (3 mL) was bubbled with HCl gas at 25 °C. The gaseous HCl was generated by dropwise addition of conc. H_2SO_4 to NH_4Cl. After stirring for 45 min, CH_2Cl_2 (1 mL) was added to the reaction with a syringe and was continued to be stirred at the same temperature. After a further 15 min, the reaction was concentrated *in vacuo* to give the intermediate compound, which was used in the next reaction without further purification. HRMS (ESI) *m*/*z* 620.1979 (620.1981 calcd for $C_{31}H_{41}ClN_3ORu$, $[M–Cl]^+$).

In another flask, a solution of carboxylic acid **II** (42.4 mg, 0.117 mmol) and the coupling agent HCTU (57.6 mg, 0.139 mmol) in CH_2Cl_2 (1 mL) was stirred at 25 °C for 30 min. To this reaction was added the intermediate compound in CH_2Cl_2 (1 mL) followed by *N*,*N*-diisopropylethylamine (137 mg, 1.06 mmol) at the same temperature. After stirring for 6 h, the reaction was stopped by adding 1 M aq. HCl. The

product was extracted with CH_2Cl_2 (×3), and the combined organic extracts were washed with sat. aq. $NaHCO_3$ and brine, dried (Na_2SO_4), and concentrated *in vacuo*. The residue was purified by silica-gel flash column chromatography (cyclohexane/EtOAc/$CHCl_3$/MeOH = 40/40/15/5) to give the desired product (37.7 mg, 35.6%) as a green solid.

^{1}H-NMR (400 MHz, $CDCl_3$, δ) 1.24–1.27 (m, 12H), 2.37–2.51 (br m, 18H), 3.47 (q, 4H, J = 7.1 Hz), 3.53–3.71 (m, 6H), 3.79 (m, 1H), 3.93 (d, 1H, J = 15.3 Hz), 3.99 (d, 1H, J = 15.3 Hz), 4.05 (dd, 1H, J_1 = J_2 = 10.0 Hz), 4.33 (dd, 1H, J_1 = J_2 = 10.0 Hz), 4.75–4.84 (br m, 1H), 4.89 (sept, 1H, J = 6.1 Hz), 6.50 (d, 1H, J = 2.3 Hz), 6.66 (dd, 1H, J_1 = 2.3 Hz, J_2 = 9.1 Hz), 6.78 (d, 1H, J = 7.7 Hz), 6.85 (dd, 1H, J_1 = J_2 = 7.7 Hz), 6.91 (dd, 1H, J_1 = 1.7 Hz, J_2 = 7.7 Hz), 7.02 (s, overlapped, 2H), 7.04 (s, 1H), 7.07 (s, 1H), 7.42 (d, 1H, J = 9.1 Hz), 7.48 (ddd, 1H, J_1 = 1.7 Hz, J_2 = J_3 = 7.7 Hz), 8.67 (s, 1H), 9.09 (s, 1H), 16.50 (s. 1H);

HRMS (ESI) *m/z* 964.3355 (964.3359 calcd for $C_{50}H_{61}ClN_5O_6Ru$, $[M–Cl]^+$).

Preparation of DABCYL Quencher

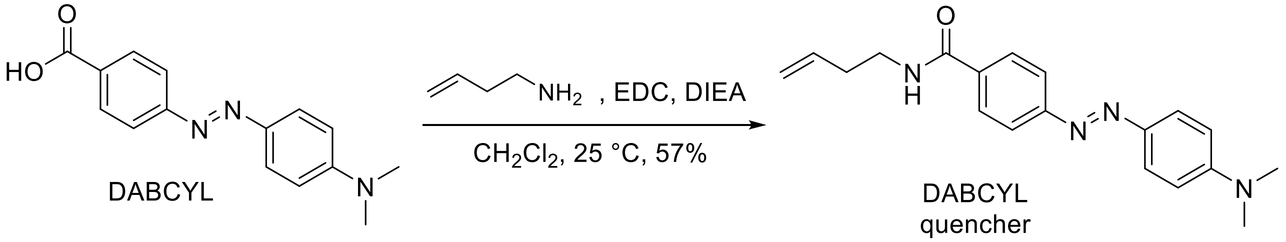

To a solution of DABCYL Acid (135 mg, 0.50 mmol), 3-buten-1-amine (81 mg, 0.75 mmol), and EDC (117 mg, 0.61 mmol) in anhydrous DCM (10 ml) was added DIEA (436 µl, 2.50 mmol) at 25 °C. After stirring for 16 h, the reaction was stopped by addition of aq. $NaHCO_3$. The product was then extracted with CH_2Cl_2 (×3), and the combined organic extracts were washed with brine, dried (Na_2SO_4), and concentrated *in vacuo*. The residue was purified by silica-gel flash column chromatography ($CHCl_3$/MeOH = 95/5) to give the desired product (91.3 mg, 57%) as an orange solid.

^{1}H-NMR (400 MHz, $CDCl_3$, δ) 2.41 (dt, 2H, J_1 = 7.2 Hz, J_2 = 7.2 Hz), 3.11 (s, 6H), 3.56 (dt, 2H, J_1 = 5.6 Hz, J_2 = 5.6 Hz), 5.13-5.20 (m, 2H), 5.82-5.89 (m, 1H), 6.22 (br s, 1H), 6.74-6.78 (m, 2H), 7.85-7.91 (m, 6H); ^{13}C-NMR (100 MHz, $CDCl_3$, δ) 167.0, 155.0, 152.8, 143.6, 135.3, 134.7, 127.7, 125.4, 122.2, 117.5, 111.5, 40.3, 38.9, 33.8;

HRMS (ESI) *m/z* 323.1858 (323.1866 calcd for $C_{19}H_{23}N_4O$, $[M+H]^+$).

Supplementary References

1. Lo, C. et al. Artificial metalloenzymes for olefin metathesis based on the biotin-(strept)avidin technology. *ChemComm* **47**, 12065-12067 (2011).
2. Kajetanowicz, A., Chatterjee, A., Reuter, R. & Ward, T. R. Biotinylated Metathesis Catalysts: Synthesis and Performance in Ring Closing Metathesis. *Catal. Lett.* **144**, 373-379 (2014).
3. Zhao, J., Kajetanowicz, A. & Ward, T. R. Carbonic anhydrase II as host protein for the creation of a biocompatible artificial metathesase. *Org. Biomol. Chem.* **13**, 5652-5655 (2015).
